# MicroRNA changes with macro potential contribute to secondary immunodeficiency in chronic lymphocytic leukemia during epstein barr virus reactivation

**DOI:** 10.1038/s41598-025-01572-4

**Published:** 2025-05-12

**Authors:** Paulina Mertowska, Sebastian Mertowski, Ewelina Grywalska

**Affiliations:** Department of Experimental Immunology, Medical University of Lublin, Chodźki 4a St., Lublin, 20-093 Poland

**Keywords:** Immunological disorders, Immunological deficiency syndromes

## Abstract

Chronic lymphocytic leukemia (CLL) is characterized by immune dysfunctions driven by miRNA deregulation and the activation of immune checkpoint pathways, which contribute to disease progression and secondary immunodeficiency (SID). This study examines the interplay between miRNA expression profiles, Epstein-Barr virus (EBV) reactivation, and immune checkpoint pathways in the context of small intestine disease (SID) development in chronic lymphocytic leukemia (CLL). Patients were stratified into groups based on the presence of SID and EBV reactivation. Comprehensive analyses included miRNA profiling, EBV infection markers, and the expression of PD-1, PD-L1, CTLA-4, CD200, and CD86 on CD4 + and CD8 + T cells, as well as CD19 + B cells. The results revealed significant suppression of tumor-suppressive miRNAs (e.g., miR-15a, miR-181a, and miR-29a) in the SID EBV + group, correlating with enhanced immunosuppression. The highest expression of exhaustion markers (PD-1, PD-L1, CTLA-4) and immunosuppressive molecules (CD200/CD200R) was observed in the SID EBV⁺ group compared to all other groups, including SID EBV⁻, which may reflect the enhanced mechanisms of immunosuppression and lymphocyte exhaustion accompanying EBV reactivation in the course of secondary immunodeficiencies. Correlation analyses underscored significant associations between miRNA levels, EBV reactivation markers, and immune checkpoint activation. These findings highlight the dual role of miRNA deregulation and immune checkpoint activation in the immunosuppressive microenvironment of CLL. The study underscores the diagnostic and therapeutic potential of miRNAs and immune checkpoints in managing SID and EBV-associated immune dysregulation in CLL.

**Supplementary Information:**

The online version contains supplementary material available at 10.1038/s41598-025-01572-4.

## Introduction

Secondary immunodeficiencies (SIDs) constitute a significant clinical challenge in chronic lymphocytic leukemia (CLL), one of the most frequently diagnosed lymphoproliferative neoplasms in adults^1–4^. It is estimated that immunological disorders, including hypogammaglobulinemia resulting from dysfunction and/or elimination of normal immunoglobulin-producing B cells, occur in up to 50–85% of patients with CLL during the disease^5,6^. The consequence of these abnormalities is a marked increase in susceptibility to recurrent infections, including opportunistic infections, which affect approximately 25–50% of patients^2^. These infections not only worsen the quality of life and reduce the systemic capacity of patients but also significantly contribute to increased mortality in this group^5,6^.

The pathophysiology of SID in CLL is highly complex and multifactorial, involving interactions between tumor cells and various components of the immune microenvironment^7,8^. The most significant changes include impaired activation and function of T lymphocytes, which assume the phenotype of exhausted cells (T cell exhaustion), characterized by a reduced ability to proliferate, secrete proinflammatory cytokines, and destroy tumor cells^9,10^. In addition, disturbances in the cytokine profile produced by CLL cells and their accompanying cells, including monocytes, macrophages, and dendritic cells, promote the creation of an immunosuppressive microenvironment, inhibiting the proper effector activity of T lymphocytes, NK cells, and other immune surveillance elements^11–14^.

The central role in modulating the function of immune system cells is played by immune checkpoints (IC) – receptor-ligand molecules that, under normal conditions, protect against excessive activation of the immune system and damage to the body’s tissues^15^. In CLL, the deregulation of key immune checkpoints (ICs), such as PD-1/PD-L1 (programmed cell death protein 1/programmed death-ligand 1), CTLA-4/CD86 (cytotoxic T-lymphocyte-associated protein 4/cluster of differentiation 86), and CD200R/CD200 (cluster of differentiation 200 receptor/ligand), is observed^8^. Overexpression of these molecules on cancer cells and immune cells, including T lymphocytes and antigen-presenting cells, results in the development of a state of T lymphocyte anergy, which leads to the silencing of the antitumor response. As a result, an immunological tolerance environment is created towards the cancer clone, which favors its survival, proliferation, and evasion of immune surveillance^16–19^.

In recent years, the growing importance of epigenetic mechanisms in regulating both neoplastic processes and immune responses has been attributed primarily to the role of microRNA (miRNA). These short, non-coding RNA molecules act as key regulators of gene expression, influencing the translation and stability of mRNA for many proteins involved in maintaining immune homeostasis, responding to stress, and controlling the dynamic balance between the quiescent state and the stimulated immune response^9,16–20^. In the pathogenesis of CLL, numerous miRNAs have been identified that are involved in key processes, including the proliferation, apoptosis, and differentiation of neoplastic cells, as well as the modulation of the immune environment and antitumor response^20,21^.

Despite the growing number of scientific reports on the role of miRNAs in CLL, a comprehensive comparative analysis considering the differences in the expression profiles of these molecules between patients with clinical features of SID and those without such disorders is still lacking [21/]. Previous studies have primarily focused on global changes in miRNA expression in patients with CLL^22–24^, which, although useful, does not allow for an unambiguous determination of the role of individual miRNAs in the development and severity of SID^25^. Meanwhile, there are indications that miRNA deregulation can reflect not only the severity of immunodeficiencies but also increased susceptibility to reactivation of latent viruses, such as Epstein-Barr virus (EBV)^26–28^.

In conditions of immunosuppression, Epstein-Barr virus (EBV) can transition from latency to an active lytic cycle, resulting in increased production of viral regulatory proteins, including latent membrane protein 1 (LMP1). LMP1, acting as an oncoprotein, significantly affects the functioning of the tumor microenvironment, including the modulation of immune checkpoint (ICP) expression and the miRNA profile^29,30^. Importantly, LMP1 functions as a functional homologue of the CD40 receptor, capable of activating intracellular signaling cascades through the recruitment of TRAF1 and TRAF2, which leads to the stimulation of NF-κB, JAK/STAT, and MAPK pathways in B cells, enhancing their survival and resistance to apoptosis^31^. Available experimental data confirm that LMP1 can increase PD-L1 expression on the surface of tumor cells and antigen-presenting cells, which consequently promotes the silencing of the immune response, enhances immunosuppression, and facilitates the progression of CLL^23,26–28,32–34^.

The observed relationships highlight the importance of a comprehensive and multidimensional analysis of the expression of key miRNAs in patients with overt symptoms of SID and those without such immune disorders. Moreover, it is necessary to consider in detail the influence of EBV – both in the latency state and during reactivation – on the variability of the miRNA profile and the deregulation of IPC pathways. Such an approach may lead to the identification of new biomarkers and potential therapeutic targets, allowing for better control of immunosuppression progression and improving the prognosis of patients with CLL.

The aim of this study was, therefore, to compare in detail the profile of selected miRNAs (miR15a-5p, miR16-5p, miR21-5p, miR28-5p, miR29a-5p, miR30c-5p, miR33a-5p, miR125b-5p, miR134-5p, miR142-5p, miR144-5p, miR150-5p, miR155-5p, miR181a-5p, miR221-5p, miR326-5p, miR486-5p, miR744-5p) in the serum of CLL patients with symptoms of secondary immunodeficiencies (SID) and CLL patients without such disorders about healthy volunteers. In this context, an analysis of differences in miRNA expression was performed, as well as the percentage of T and B cells expressing key immune checkpoints, including PD-1/PD-L1, CTLA-4/CD86, and CD200R/CD200. Additionally, the influence of EBV reactivation on the miRNA profile and the activity of regulatory pathways was taken into account, which allowed for expanding the knowledge of the complex network of relationships between the state of immunosuppression, epigenetic regulation of gene expression, and the immunomodulatory effects of viral infections.

## Results

### The role of MiRNAs and immune checkpoints in shaping the immunosuppressive microenvironment of CLL

The miRNAs selected for analysis were chosen based on literature reports and the results of our previous studies. The presented miRNAs play diverse yet crucial roles in regulating various cellular processes, including proliferation, apoptosis, differentiation, and the control of signaling pathways essential for neoplastic transformation. In the context of CLL and other hematological malignancies^21^, the importance of miRNAs is particularly significant because disorders in their expression profile can affect the survival of cancer cells, resistance to apoptosis, and the creation of a favorable microenvironment (Table 1).

**Table 1:** Overview of selected MicroRNAs (miRNAs) analyzed in the study, their known or proposed biological significance.

Type of miRNA	Biological significance	References
miR15a-5p	These miRNAs are located in the 13q14 region, which is frequently deleted in CLL. Their loss leads to the overexpression of BCL2, which promotes cancer cell survival. Studies have shown that miR-15a-5p and miR-16-5p function as tumor suppressors, and their decreased expression is associated with the progression of CLL.	36, 37
miR16-5p		
miR21-5p	A known oncomiR, overexpression of which has been observed in various cancers, including CLL. miR-21-5p plays a role in inhibiting apoptosis and promoting cancer cell survival by regulating tumor suppressor genes.	38, 39
miR28-5p	miR-28-5p is overexpressed in CLL cells and may play an oncogenic role by inhibiting the expression of the tumor suppressor gene NDRG2. Dysregulation of this pathway may promote the survival and proliferation of leukemic cells.	40, 41
miR29a-5p	Its deregulation has been described in CLL, where it affects the differentiation and function of B and T lymphocytes.	42, 43
miR30c-5p	Associated with various cancers, its role in CLL is not fully understood, but it may influence immunological mechanisms and disease progression	44
miR33a-5p	Regulator of lipid metabolism and cancer cell proliferation, may influence the tumor microenvironment in CLL	45
miR125b-5p	Modulates interferon-like response and T cell function, which may be important in the context of SID and EBV reactivation in patients with CLL	46, 47
miR134-5p	Plays a key role in various aspects of cancer biology, such as cell proliferation, apoptosis, invasion, metastasis and drug resistance	48
miR142-5p	Influences monocyte/macrophage activity and T cell functions, which can be important in the context of the immune response in CLL.	49
miR144-5p	miR-144-5p acts as a tumor suppressor in many types of cancer by inhibiting tumor cell proliferation, migration, and invasion and promoting apoptosis. It also influences the regulation of immune pathways and may modulate antiviral responses, making it a potential candidate for studies of secondary immunosuppression and EBV reactivation.	50
miR150-5p	Known for its deregulation in CLL, it affects the differentiation and functioning of B and T lymphocytes, which may be important in the pathogenesis of SID	51, 35
miR155-5p	OnkomiR, whose overexpression is associated with a more aggressive form of CLL and deregulation of the immune response.	52, 21
miR181a-5p	miR-181a-5p promotes leukemic cell proliferation via activation of the Wnt signaling pathway in acute lymphoblastic leukemia (ALL). As a Wnt inducer, it may also play a role in the progression of other hematological malignancies, including CLL, by deregulating cell growth control mechanisms.	53, 54
miR221-5p	miR-221-5p shows significantly higher expression in CLL patients with low risk of disease progression (stage 0), compared to high-risk patients. Studies indicate that miR-221, alongside miR-181a and miR-223, has significant diagnostic potential in distinguishing between different stages of CLL advancement.	55, 56
miR326-5p	It is a microRNA involved in the regulation of various biological processes, including tumor progression and immune response. In the context of childhood acute lymphoblastic leukemia (ALL), studies have shown that plasma miR-326 levels can serve as a prognostic biomarker. Elevated miR-326 expression in plasma exosomes was associated with primary treatment resistance, suggesting its potential use in the diagnosis and therapy of ALL. In other cancers, such as prostate cancer, miR-326 acts as a tumor suppressor, inhibiting tumor cell proliferation, migration, and invasion by regulating genes related to cell cycle and apoptosis.	57, 58
miR486-5p	miR-486-5p acts as a tumor suppressor, and its reduced expression has been shown in chronic myeloid leukemia (CML), where it correlates with poorer clinical response. Studies suggest that miR-486-5p inhibits leukemic cell proliferation and may be a useful prognostic biomarker and potential therapeutic target.	59, 60
miR744-5p	miR-744-5p exerts tumor suppressor activity in multiple myeloma by inhibiting tumor cell proliferation, epithelial-mesenchymal transformation (EMT), and glycolysis. This mechanism is associated with direct inhibition of SOX12 expression and attenuation of the Wnt/β-catenin pathway, suggesting a potential role of miR-744-5p as a regulator of aggressiveness and metabolism in hematological malignancies.	61

The serum miRNA profiling was performed using digital PCR (dPCR). Previous literature data on miRNAs in CLL focus mainly on techniques based on qPCR^15,61^ The use of dPCR represents a significant methodological improvement, as it enables the precise and absolute quantification of molecules, is less susceptible to PCR inhibitors, and eliminates the need for standard curves. Thanks to this, dPCR enables a more reliable assessment of differences in miRNA expression between the study groups, particularly in serum miRNA analyses.

**Fig. 1:**
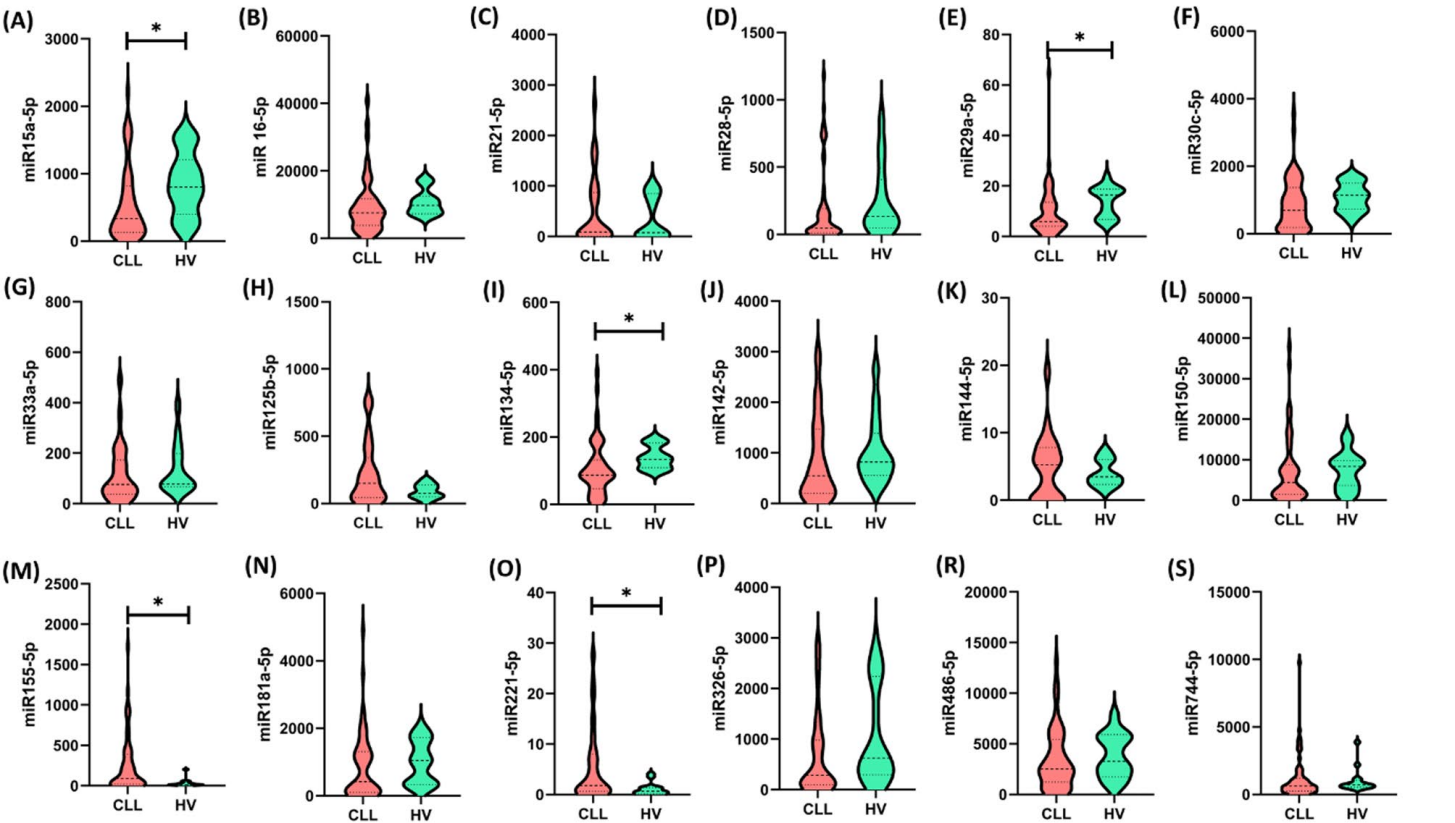
Serum levels of selected microRNAs (miRNAs) from patients with chronic lymphocytic leukemia (CLL, *n* = 60; red graphs) and healthy volunteers (HV, *n* = 15; green graphs), as determined by digital PCR (dPCR). miRNA concentration was measured by digital PCR using QIAcuity Nanoplate 8.5k plates, in which each sample was analyzed in 8500 individual nanopartitions, and the result was an average value calculated automatically by QIAcuity software based on fluorescent signals. Each panel (A–S) shows the distribution of expression values ​​of a specific miRNA (in copies/µL) in both study groups. Violin plots show both the median and the density of the data distribution in each group. Statistically significant differences (*p* < 0.05) were observed between the CLL and HV groups for the following miRNAs: miR-15a-5p (A), miR-29a-5p (E), miR-134-5p (I), miR-142-5p (J), miR-155-5p (M), and miR-221-5p (O). The indicated molecules showed significant increases or decreases in serum levels of CLL patients compared to healthy individuals, suggesting their potential biological and diagnostic significance. In the case of the remaining miRNAs, although visible differential trends were observed in some instances, the differences did not reach the threshold of statistical significance. Asterisks (*) indicate statistically significant differences between the CLL and HV groups (*p* < 0.05).

In the initial phase of our study, we compared the entire group of patients with chronic lymphocytic leukemia (CLL, *n* = 60) with a group of healthy volunteers (*n* = 15) who served as the control group. This approach allowed for the preliminary assessment of differences in the levels of individual miRNA molecules between patients and healthy controls. The results of these analyses are presented in Fig. 1 and Table S1 in the supplementary materials.

**Fig. 2:**
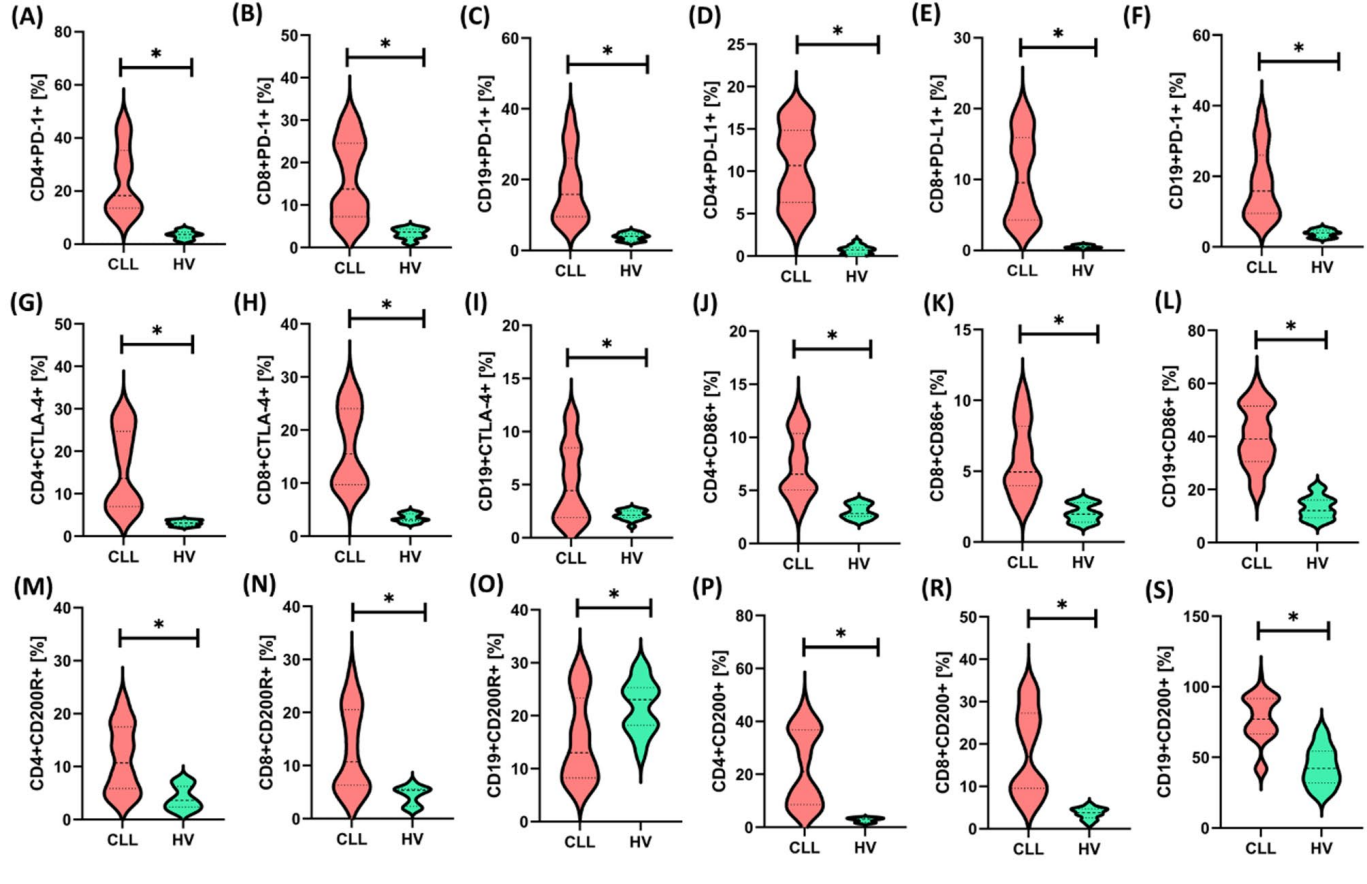
Percentage expression of selected immune checkpoints and their ligands on the surface of CD4⁺ T cells, CD8⁺ T cells, and CD19⁺ B cells, assessed in peripheral blood samples from patients with chronic lymphocytic leukemia (CLL, *n* = 60; red graphs) and healthy volunteers (HV, *n* = 15; green graphs). Analyses were performed using flow cytometry, and the presented percentage values ​​(e.g. CD4 + PD-1-%) refer to the percentage of cells positive for a given marker within a defined cell population – CD4+, CD8 + or CD19+, respectively – selected based on FSC/SSC gating and the expression of the corresponding surface markers. Each panel (A–S) presents the data distribution in the form of a violin plot, including the median (dashed line) and the density of the distribution of values ​​for a given cell subpopulation. The expression of inhibitory receptors such as PD-1 (A–C), PD-L1 (D–F), CTLA-4 (G–I), the co-stimulatory molecule CD86 (J–L), and the immunomodulatory molecule CD200 (M–S) was analyzed among both the T CD4⁺, T CD8⁺, and B CD19⁺ subpopulations. In all cases, a significantly higher percentage of cells expressing the analyzed molecules was observed in the CLL group compared to HV (*p* < 0.05), as indicated by asterisks (*). Increased expression of inhibitory receptors and suppressor molecules may reflect both the state of lymphocyte exhaustion (T-cell exhaustion) and the presence of an immunosuppressive microenvironment characteristic of CLL.

Among the analyzed miRNAs, we observed clear and statistically significant differences in their expression levels between the serum of CLL patients and healthy volunteers. In particular, miR-15a-5p, miR-29a-5p, and miR-134a-5p were significantly reduced in CLL patients compared to healthy volunteers, and miR-155-5p and miR-221-5p showed significantly increased levels in CLL patients. Some of them (e.g., miR-15a-5p, miR-29a-5p) have already been linked to processes related to the pathogenesis of hematological malignancies, including the regulation of cancer cell proliferation and apoptosis. Also, it is worth emphasizing that miR-155-5p and miR-221-5p, included in our analysis, are involved in the activation of signaling pathways important for the pathogenesis of CLL^9,10,12–14^. After identifying significant differences in the expression of selected miRNAs, we next focused on analyzing the frequency of immune checkpoints and their ligands on the surface of CD4 + T cells, CD8 + T cells, and CD19 + B cells to obtain a more comprehensive picture of the relationship between the miRNA profile and the immune status in patients with CLL. The obtained results are illustrated in Fig. 2 and Supplementary materials Table S3.

**Fig. 3:**
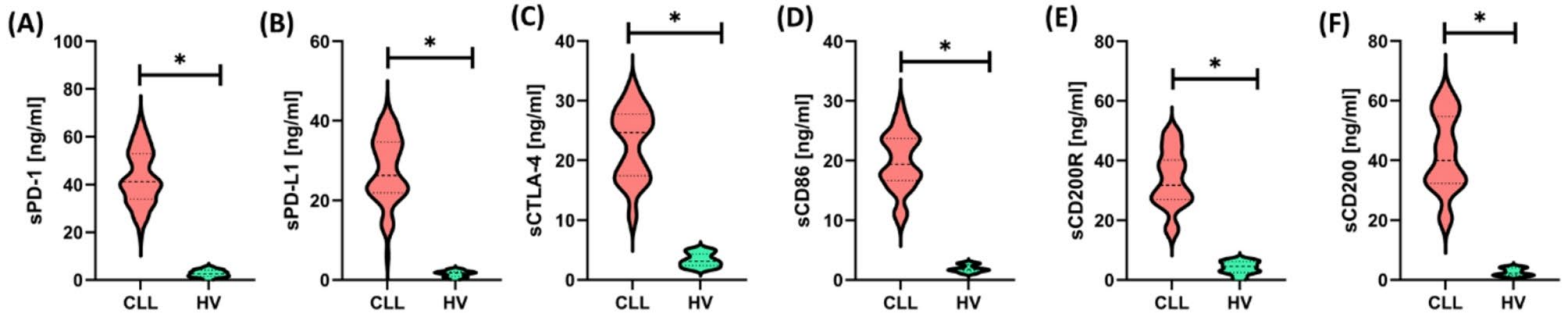
Concentration of soluble forms of selected immune checkpoint molecules and their ligands in serum of patients with chronic lymphocytic leukemia (CLL, *n* = 60; red graphs) compared to healthy volunteers (HV, *n* = 15; green graphs). The presented results are from an immunoenzymatic test used to determine the concentrations of soluble forms of immune checkpoint molecules in the serum of the studied individuals. Presented are sPD-1 (A), sPD-L1 (B), sCTLA-4 (C), sCD86 (D), sCD200R (E), and sCD200 (F), expressed in ng/mL. Violin plots show the data distribution, median (dashed line), and density of values ​​for each of the analyzed molecules in both study groups. In all cases, significantly higher concentrations of soluble molecules were observed in the serum of patients with CLL compared to healthy individuals (*p* < 0.05), as indicated by an asterisk (*). Elevated levels of sPD-1, sPD-L1, sCTLA-4, sCD86, sCD200R, and sCD200 may reflect enhanced immunosuppressive mechanisms that accompany disease progression and promote evasion of immune surveillance by cancer cells.

In the analyzed group of patients with CLL, a significantly lower percentage of T lymphocytes (CD3+), including both CD4 + and CD8 + subpopulations, was observed compared to healthy volunteers (HV), with a simultaneous significant increase in the percentage of B lymphocytes CD19+ (*p* < 0.000 for all parameters). The CD4+/CD8 + ratio was significantly lower in patients with CLL than in the control group (*p* = 0.040), which indicates immune imbalance typical for this disease. There was an apparent increase in the percentage of T cells (both CD4 + and CD8+) and CD19 + B cells expressing immune inhibitory molecules (e.g., PD-1, CTLA-4, CD200R) and immunomodulatory ligands (e.g., PD-L1, CD86, CD200) in patients with CLL compared to HV. Higher expression of these checkpoints and ligands may indicate a more immunosuppressive microenvironment in CLL, which favors the evasion of immune surveillance by tumor cells. Such phenotypic changes may have prognostic and therapeutic significance, suggesting the potential efficacy of therapies targeting checkpoint blockade to enhance the immune response against leukemic cells. To obtain a more comprehensive understanding of the immune status of CLL patients, after determining the frequency of immune checkpoints and their ligands on the surface of lymphocytes, we also analyzed the concentrations of these molecules directly in serum (Fig. 3 and Supplementary Materials Table S4).

Significantly higher levels of all studied molecules were observed in the serum of patients with CLL, which may indicate increased activity of immunosuppressive signaling pathways in the course of this disease. High levels of soluble forms of checkpoints and their ligands may promote the suppression of the immune response against cancer cells, facilitating their evasion of immune surveillance and contributing to disease progression. These results support the hypothesis that pathways that inhibit the immune response are crucial for the CLL microenvironment and represent a potential therapeutic target.

#### ***Relationships between miRNA expression***,*** immune checkpoints and their soluble forms***,*** EBV reactivation***,*** and secondary immunodeficiencies in patients with CLL***

In the next stage of the study, to obtain a more comprehensive understanding of the roles of selected miRNAs and immune checkpoints in the pathogenesis and progression of CLL, we decided to analyze patient profiles in greater detail, taking into account the presence of SID and EBV reactivation status. For this purpose, patients were divided into four equal subgroups (*n* = 15 each): those with symptoms of secondary immunodeficiency (SID), those without SID symptoms, those with Epstein–Barr virus (EBV) reactivation, and those without EBV reactivation (Supplementary Materials, Table S2). Such division enabled us not only to associate differences in miRNA expression and cell phenotype with specific clinical conditions but also to gain a better understanding of the potential interactions between viral factors and weakened immunity in the context of CLL. For this purpose, we analyzed the infectious status of patients, taking into account the need for antibiotic therapy within the 12 months preceding diagnosis and the serological profile, and assessed the number of EBV virus copies in individual patients (Supplementary Materials Table S5). The first analyzed aspect revealed that, within the range of 0 to 1 infections requiring antibiotic therapy, the CLL EBV − group predominated, with the highest number (10 patients), while only 2 patients were found in the CLL EBV + group. The SID EBV + and SID EBV − groups were not represented in this range. In the range from 2 to 4 infections in the last 12 months that required antibiotic therapy, the most patients were found in the CLL EBV + group (8 patients), while in the CLL EBV − group the number was 5 patients. In the range from 5 to 7 infections, the number of patients was equal in the SID EBV + and CLL EBV + groups (4 patients each), while the SID EBV − group was not represented. In the range of 8 to 10 infections, the highest number was noted in the SID EBV − group (14 patients), while the SID EBV + group had 6 patients in this category. In the range of more than 10 infections within 12 months requiring antibiotic therapy, the SID EBV + group dominated (10 patients), which may indicate a stronger EBV reactivation in combination with secondary immunodeficiencies. In the other groups, patients were not represented in this category. A detailed analysis of the type of infections requiring antibiotic therapy among the patients recruited to the study showed that the highest percentage of upper respiratory tract infections was noted in the SID EBV − group (80%), while in the SID EBV + group, it was 50%. In the CLL EBV + and CLL EBV − groups, the percentage of patients with this type of infection was significantly lower and amounted to 30% and 10%, respectively. In the case of lower respiratory tract infections, the SID EBV + group was dominant with a percentage of 70%, while in SID EBV − it was 40%. The CLL EBV + group was characterized by a lower proportion of patients (20%), and in CLL EBV − it was only 10%. Urinary tract infections occurred most frequently in the SID EBV + group (40%), while in the SID EBV − and CLL EBV + groups the percentage of patients was similar (20%). In the CLL EBV − group, these infections were noted in 10% of patients. The highest frequency of gastrointestinal infections was observed in the SID EBV + group (80%) and SID EBV− (60%), while in the CLL EBV + group, these infections occurred rarely (10%), and in CLL EBV−, they were not noted at all. Skin and soft tissue infections occurred mainly in the SID EBV+ (20%) and SID EBV− (10%) groups, while in the CLL EBV + and CLL EBV − groups, there was no significant share of patients with this type of infection. The obtained results indicate that patients with SID, especially with concomitant EBV reactivation, are most susceptible to infections, especially of the upper and lower respiratory tract and gastrointestinal tract. The SID EBV − group was also characterized by a significant frequency of infections, although slightly lower compared to SID EBV+. Patients with CLL EBV + and CLL EBV − showed a lower percentage of infections, which suggests a lesser degree of immune function impairment compared to patients with SID. Studies aimed at determining EBV reactivation in the studied patients not only allowed them to be assigned to specific groups but also showed a statistically significant increase in the number of EBV virus copies in patients with symptoms of SID compared to CLL patients without signs of SID.

**Fig. 4:**
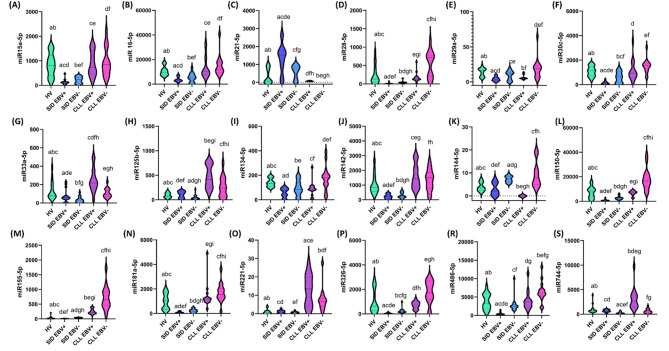
Expression levels of selected microRNAs (miRNAs) in serum of healthy volunteers (HV, green), patients with secondary immunodeficiencies with EBV reactivation (SID EBV+, blue) and without EBV reactivation (SID EBV-, navy blue), and patients with chronic lymphocytic leukemia with EBV reactivation (CLL EBV+, purple) and without it (CLL EBV-, pink). Each group consisted of 15 patients. miRNA concentration was measured by digital PCR using QIAcuity Nanoplate 8.5k plates, in which each sample was analyzed in 8500 individual nanopartitions, and the result was an average value calculated automatically by QIAcuity software based on fluorescent signals. Values ​​are presented in the form of violin plots, illustrating the data distribution, median, and density of values ​​for each group. Each panel (A–S) corresponds to the analysis of a specific miRNA. Letter designations (e.g., a, b, c, etc.) indicate statistically significant differences between the comparative groups (*p* < 0.05). Differential patterns of miRNA expression were observed between the analyzed groups, with some miRNAs exhibiting decreased or increased levels depending on the presence of EBV reactivation and the underlying disease entity (SID vs. CLL). These results highlight the potential use of selected miRNAs as biomarkers differentiating not only the patient’s immune status but also the reactivation of latent EBV infection.

To better understand the mechanisms underlying increased susceptibility to infections and immunosuppression in CLL patients, in the next analysis, we focused on the assessment of miRNA expression profiles and immune checkpoints and their ligands in individual patient groups (Supplementary materials Table S6-S8). In the case of miRNA analyses, we observed a clear trend of gradual decrease in the levels of miR15a-5p (Fig. 4A), miR16-5p (Fig. 4B), miR28-5p (Fig. 4D), miR142-5p (Fig. 4J), miR150-5p (Fig. 4L), miR155-5p (Fig. 4M), miR181a-5p (Fig. 4N), miR326-5p (Fig. 4P), and miR486-5p (Fig. 4R) depending on the EBV reactivation status and the presence of SID (Fig. 4). The lowest miRNA values ​​in the SID EBV + group emphasize the key role of EBV in the exacerbation of immunosuppression and weakening of defense mechanisms, which may promote disease progression and increased susceptibility to infections. Additionally, miR21-5p (Fig. 4C) deserves special attention, for which an opposite trend was observed, which means that for patients with SID EBV+, the values ​​were the highest, and in the case of patients with CLL EBV- no presence of the tested molecule was noted at all (Fig. 4). In the case of miR-29a-5p (Fig. 4E), a decrease in its levels was noted in patients with EBV reactivation compared to patients without EBV reactivation, regardless of the study group. In turn, the analysis of miR-33a-5p (Fig. 4G) and miR-125b-5p (Fig. 4H) showed the opposite effect - EBV reactivation led to an increase in the levels of these miRNAs (Fig. 4).

**Fig. 5:**
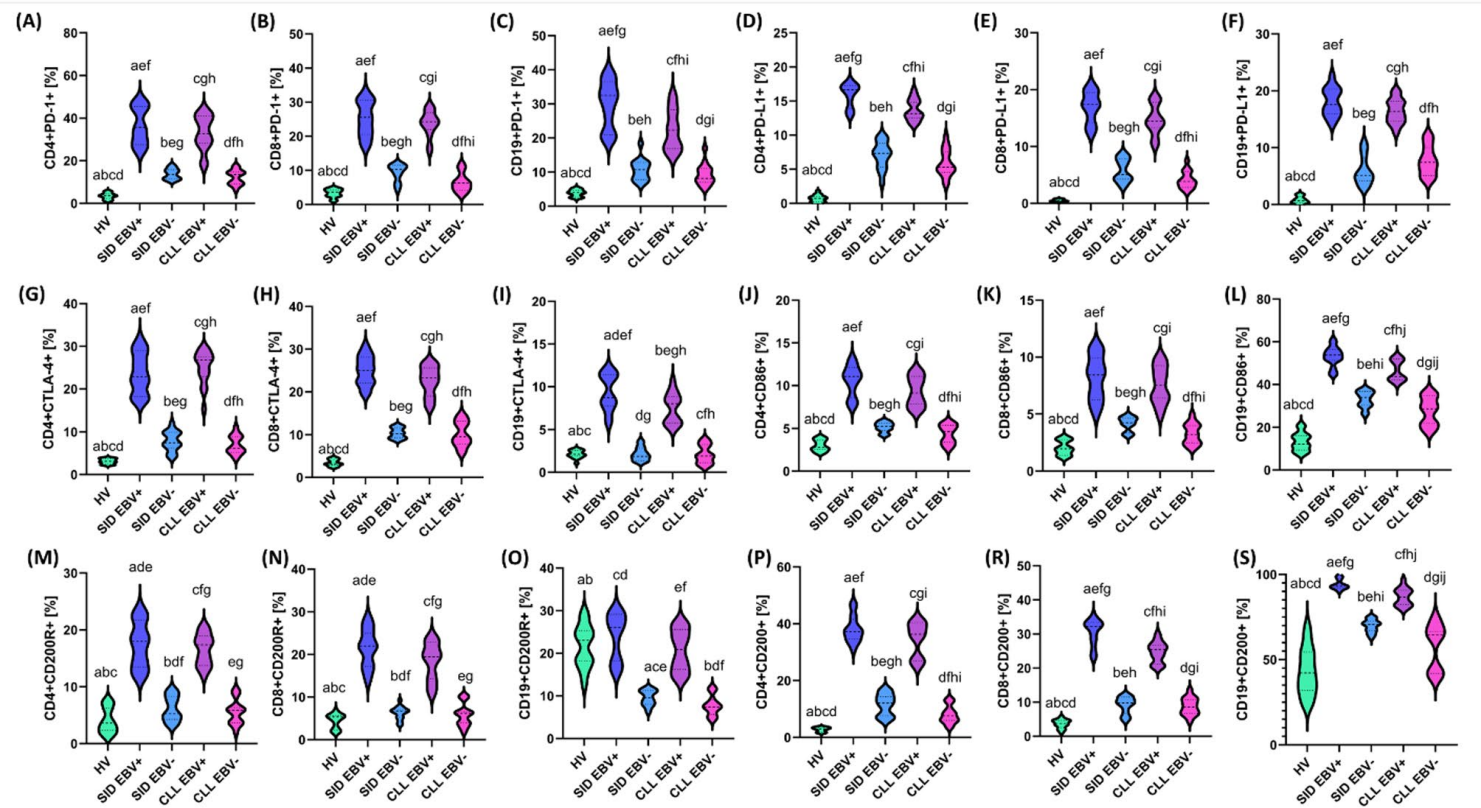
The percentages of CD4⁺, CD8⁺ and CD19⁺ B cells expressing selected immune checkpoint molecules and their ligands were assessed in different study groups: healthy volunteers (HV, green), patients with secondary immunodeficiencies with EBV reactivation (SID EBV⁺, blue) and without EBV reactivation (SID EBV⁻, navy blue), and patients with chronic lymphocytic leukemia with EBV reactivation (CLL EBV⁺, purple) and without (CLL EBV⁻, pink). Each group consisted of 15 patients. Analyses were performed using flow cytometry, and the presented percentage values ​​(e.g. CD4 + PD-1-%) refer to the percentage of cells positive for a given marker within a defined cell population – CD4+, CD8 + or CD19+, respectively – selected based on FSC/SSC gating and the expression of the corresponding surface markers. Each panel (A–S) illustrates the percentage distribution of cells expressing a given surface molecule, presented as violin plots with the median and density of the data distribution indicated. The analyzed molecules include inhibitory receptors (PD-1, CTLA-4), their ligands (PD-L1), co-stimulatory markers (CD86), and immunomodulatory markers (CD200, CD200R), which allows for a comprehensive characterization of the activation and immunosuppression status of the studied cell subpopulations. Letter designations (e.g., a, b, c, etc.) indicate statistically significant differences between groups (*p* < 0.05). The results reveal significant differences in the immune phenotype between the analyzed cohorts, especially in the expression of inhibitory and immunosuppressive molecules, which may be of significant importance in assessing lymphocyte exhaustion (T cell exhaustion) and immunoregulation during the course of CLL and in conditions of EBV reactivation.

The comparison of the analyzed groups showed significant differences in the composition of lymphocyte subpopulations. The percentage of T cells (CD3+), CD4 + and CD8 + was significantly reduced in the CLL EBV + and CLL EBV- groups compared to healthy volunteers (HV), while the highest percentage of CD19 + B lymphocytes was observed in patients with CLL, regardless of EBV status (*p* < 0.000). In the SID EBV + group, the percentage of T lymphocytes (CD3+) was significantly higher than in the CLL EBV- group (*p* = 0.045), and at the same time clearly lower than in healthy volunteers (*p* < 0.000) (Supplementary Materials Table S7). To assess the impact of immune dysregulation and EBV reactivation on immunosuppression in patients with CLL and SID, we analyzed the expression of immune checkpoints and their ligands on the surface of CD4 + T cells, CD8 + T cells, and CD19 + B cells, as well as the levels of their soluble forms in serum in the individual study groups (Fig. 5A-S). The percentage of PD-1 and PD-L1 on CD4+ (Fig. 5A and D), CD8+ (Fig. 5B and E) T cells, and CD19 + B (Fig. 5C and F) cells was highest in the SID EBV + and CLL EBV + groups, indicating the activation of immunosuppressive pathways in response to EBV reactivation. Lower levels were found in the CLL EBV − and SID EBV − groups, while the lowest values ​​were characteristic of HV. A similar pattern was observed for CTLA-4, the highest percentages of which were found on the surface of CD4+(Fig. 5G), CD8+ (Fig. 5H) T cells, and CD19 + B cells (Fig. 5I) in the SID EBV + and CLL EBV + groups, suggesting significant functional exhaustion of these cells as a result of chronic immune stimulation. Intermediate values ​​were obtained for SID EBV − and CLL EBV−, while the lowest levels were observed in the HV group, reflecting normal homeostasis in the immune system. The percentage of CD86, a costimulatory marker, was significantly increased in the SID EBV + and CLL EBV + groups, both on CD4+ (Fig. 5J) and CD8+ (Fig. 5K) T cells, as well as on CD19 + B (Fig. 5L) cells, which may indicate an enhanced activation of immune cells in response to EBV. The levels in the SID EBV − and CLL EBV − groups were lower, with the lowest values ​​observed in the HV group. The highest expression values of CD200 (Fig. 5M-O) and its receptor CD200R (Fig. 5P-S) were observed in the SID EBV + and CLL EBV + groups, highlighting the role of this pathway in the induction of immunosuppression and inhibition of its activity. The obtained results showed that the percentage of analyzed immune cell populations was the highest in the SID EBV + and CLL EBV + groups compared to the corresponding EBV-negative groups, which may indicate a significant impact of EBV virus reactivation on the immunophenotype of lymphocytes.

**Fig. 6:**
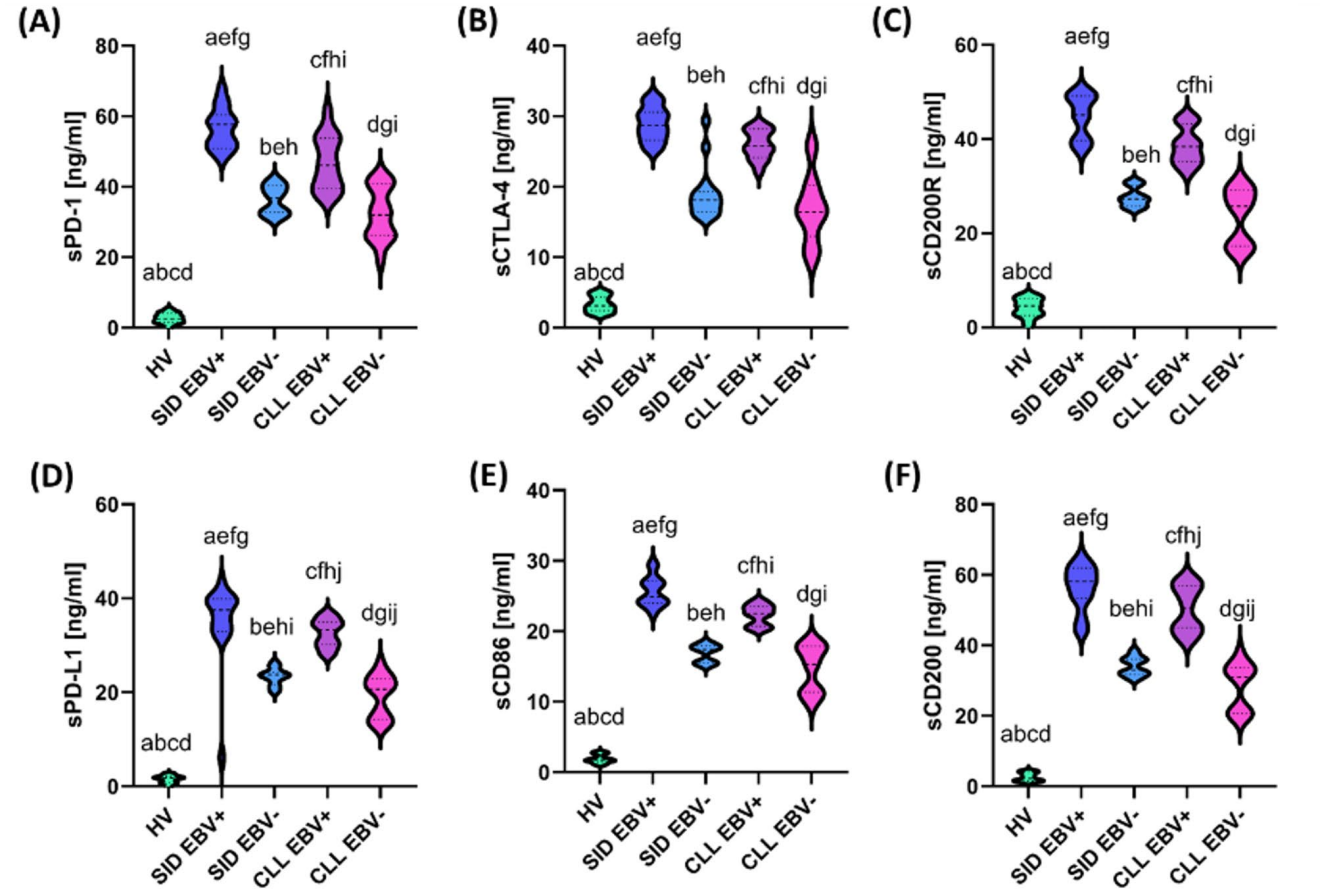
Serum levels of soluble forms of selected immune checkpoint molecules and their ligands in study participants, categorized by the presence of EBV reactivation and immunodeficiency. Shown are sPD-1 (A), sCTLA-4 (B), sCD200R (C), sPD-L1 (D), sCD86 (E), and sCD200 (F). Data are presented as violin plots, showing the distribution of values ​​in the following groups: healthy volunteers (HV, green), patients with secondary immunodeficiencies with EBV reactivation (SID EBV⁺, blue) and without it (SID EBV⁻, navy blue), and patients with chronic lymphocytic leukemia with EBV reactivation (CLL EBV⁺, purple) and without it (CLL EBV⁻, pink). Each group consisted of 15 patients. The presented results are from an immunoenzymatic test used to determine the concentrations of soluble forms of immune checkpoint molecules in the serum of the studied individuals. The graphs display the median and density of the concentration distribution, expressed in ng/mL. Letter designations (e.g., a, b, c, etc.) indicate statistically significant differences between the individual groups (*p* < 0.05). The applied analysis enables the identification of differences in the levels of soluble immunoregulatory molecules, dependent on the presence of EBV reactivation and the state of immunosuppression. These results emphasize the potential value of serum markers associated with immune checkpoints in assessing the immune status of patients with immunodeficiencies and/or chronic lymphocytic leukemia.

A similar trend was observed in the analyses of the concentrations of soluble forms of the studied immune checkpoints and their ligands, where the highest values ​​were noted in patients with EBV reactivation, especially in the SID group, compared to patients without EBV reactivation and HV (Fig. 6A-F).

**Fig. 7:**
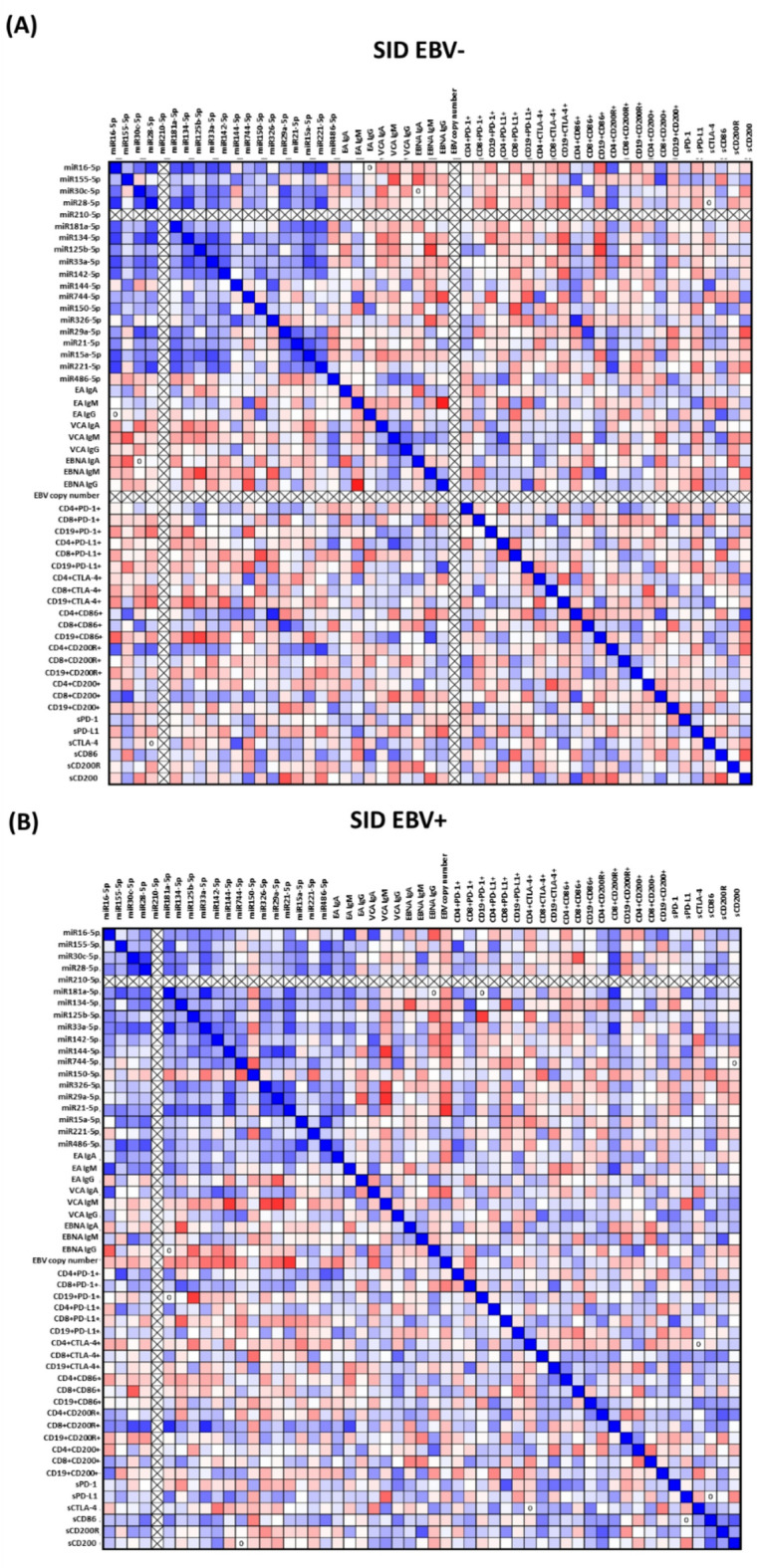
Graphical representation of Spearman rank correlation results for patients with chronic lymphocytic leukemia (CLL) and secondary immunodeficiency syndrome (SID), categorized into two groups: those with EBV reactivation (EBV SID⁺) and those without it (EBV SID⁻). Each of the analyzed groups consisted of 15 patients. Matrices show correlations between miRNA levels, EBV serological parameters (EA IgA, VCA IgG, EBNA, etc.), EBV copy number, percentage of cells expressing surface molecules (e.g. PD-1, PD-L1, CTLA-4, CD200/CD200R), and concentrations of their soluble forms in serum (sPD-1, sCD86, sCD200R, etc.). Positive correlations are marked in blue, indicating that an increase in the value of one variable is associated with an increase in the value of the other. Negative correlations are marked in red, indicating an inverse relationship between parameters. The more intense the color of a given field, the stronger the degree of correlation.

Correlation analysis between miRNA levels, EBV infection markers, and immunological parameters revealed significant relationships that allow for a better understanding of their interrelationships in the context of EBV reactivation and progression of immunosuppression. In SID patients with EBV reactivation, the strongest negative correlations were observed for miR-29a-5p and VCA IgM (*R* = −0.803, *p* < 0.001), as well as for miR-21-5p with EBV copy number (*R* = −0.786, *p* < 0.001), suggesting an inverse relationship between the levels of these miRNAs and EBV activation markers. Similar correlations were found for miR-144-5p with VCA IgM (*R* = −0.782, *p* < 0.001) and miR-125b-5p with CD19 + PD-1+ (*R* = −0.768, *p* = 0.001), which may indicate the role of miRNAs in limiting virus replication and in regulating the expression of immunosuppressive markers on the surface of B cells. It is worth noting the positive correlations between selected miRNAs and markers of immunosuppression, e.g., miR-155-5p and CD4 + PD-1+ (*R* = 0.711, *p* = 0.003) and miR-30c-5p with CD8 + CD200R+ (*R* = 0.714, *p* = 0.004). These results indicate the participation of these miRNAs in the activation of immunosuppressive pathways that lead to the functional exhaustion of immune cells. In turn, strong correlations between miRNAs, such as miR-181a-5p and miR-33a-5p (*R* = 0.900, *p* < 0.001), miR-15a-5p and miR-486-5p (*R* = 0.818, *p* < 0.001), and miR-28-5p and miR-486-5p (*R* = 0.686, *p* = 0.005), suggest interdependence in the regulation of their expression and potential participation in common mechanisms of immune or antiviral response (Supplementary materials Table S9; Fig. 7B).

Correlation analysis for SID EBV patients showed significant relationships between miRNA levels, EBV activation markers, and immunological parameters. Strong negative correlations were observed for miR-125b-5p with EBNA IgM (*R* = −0.746, *p* = 0.001) and miR-744-5p with EBNA IgG (*R* = −0.693, *p* = 0.004), suggesting that reduced levels of these miRNAs may be associated with the activation of antiviral pathways in the context of low-level EBV reactivation. A negative correlation between miR-150-5p and CD8 + PD-L1+ (*R* = −0.691, *p* = 0.004) indicates a possible involvement of this miRNA in the regulation of immunosuppression by inhibiting PD-L1 expression on CD8 + cytotoxic cells. Similar correlations were observed for miR-29a-5p with CD8+ (*R* = −0.690, *p* = 0.004) and miR-30c-5p with CD45 + marker (*R* = −0.675, *p* = 0.006), which may indicate limited activity of immune response in this group of patients. Significant positive correlations were found for miRNA levels, such as miR-221-5p with miR-28-5p (*R* = 0.753, *p* = 0.001), miR-16-5p with miR-28-5p (*R* = 0.814, *p* < 0.001), and miR-134-5p with miR-28-5p (*R* = 0.800, *p* < 0.001), suggesting interdependent regulation of these miRNAs in the context of immune mechanisms. The positive correlation of miR-326-5p with CD4 + CD86+ (*R* = 0.861, *p* < 0.001) and miR-29a-5p with CD4/CD8 + ratio (*R* = 0.735, *p* = 0.002) indicates the important role of these miRNAs in regulating the activation of CD4 + helper lymphocytes and maintaining the balance between T cell subpopulations. Moreover, positive correlations between miR-144-5p and sCTLA-4 levels (*R* = 0.668, *p* = 0.007) and between miR-155-5p and CD8 + CD200+ (*R* = 0.718, *p* = 0.003) emphasize their potential participation in the mechanisms of immunosuppression through the activation of checkpoints. Negative correlations of miRNAs with infection markers, such as VCA IgM and EBNA IgA, suggest a role of these miRNAs in inhibiting EBV replication and limiting its activity in the group of patients with EBV − SID (Supplementary materials Table S10; Fig. 7A).

**Fig. 8:**
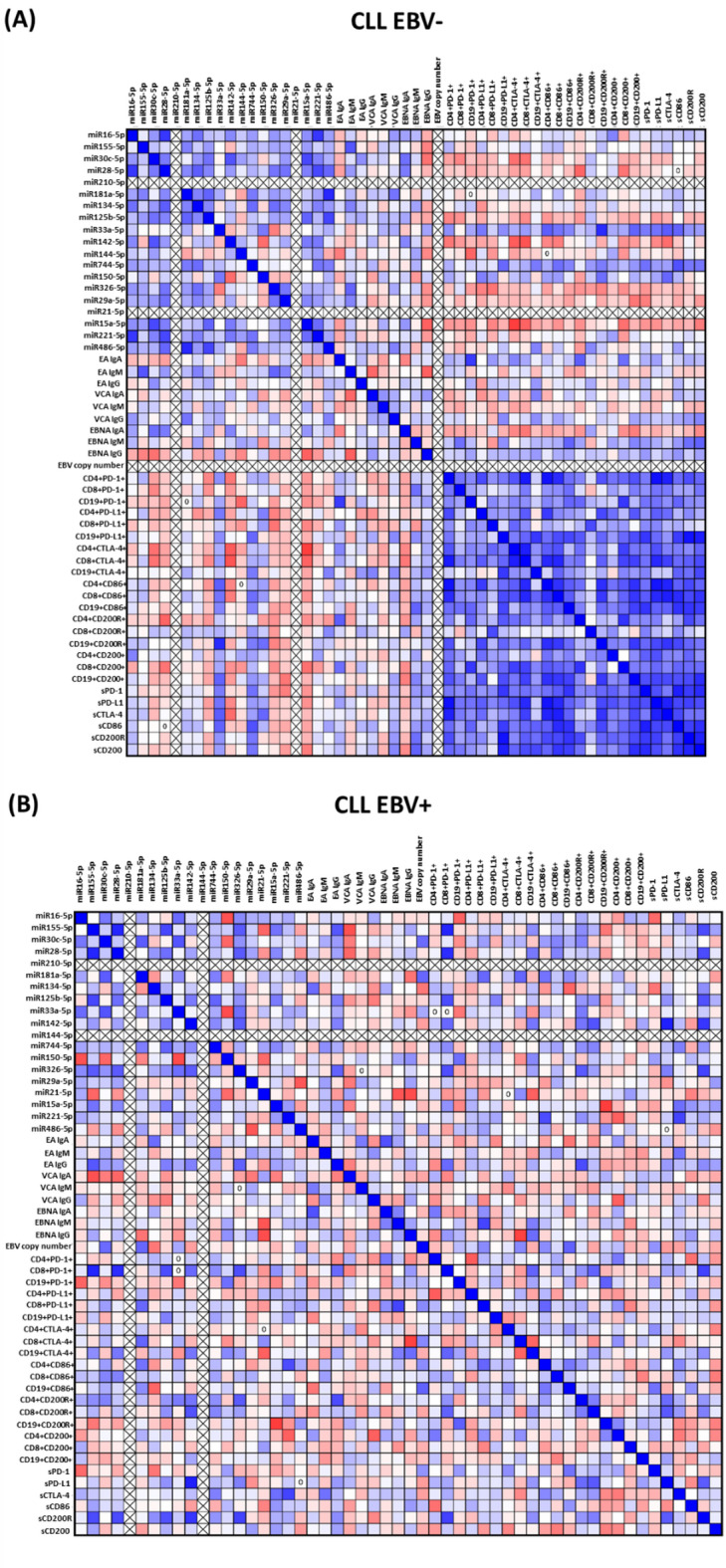
Graphical representation of Spearman rank correlations in patients with chronic lymphocytic leukemia (CLL) with Epstein–Barr virus (EBV) reactivation. Each of the analyzed groups consisted of 15 patients. The matrices show the correlations between miRNA levels, EBV serological parameters (EA IgA, VCA IgG, EBNA, etc.), EBV copy number, percentage of cells expressing surface molecules (e.g. PD-1, PD-L1, CTLA-4, CD200/CD200R), and concentrations of their soluble forms in serum (sPD-1, sCD86, sCD200R, etc.). Positive correlations are marked in blue, indicating that an increase in the value of one variable is associated with an increase in the value of the other. Negative correlations are marked in red, indicating an inverse relationship between parameters. The more intense the color of a given field, the stronger the degree of correlation.

Correlation analysis for EBV + CLL patients showed significant associations between miRNA levels, EBV reactivation markers, and immunological parameters. The strongest negative correlations were observed for miR-15a-5p with CD19 + CD200R+ (*R* = −0.764, *p* = 0.001), miR-21-5p with EBNA IgG (*R* = −0.700, *p* = 0.004) and miR-155-5p with VCA IgA (*R* = −0.686, *p* = 0.005). These results suggest that miRNAs may play a key role in limiting EBV virus replication and regulating immunosuppressive pathways activated on B cells. In turn, positive correlations reveal the association of miRNAs with immunosuppression markers. The strongest correlation was observed between miR-142-5p and sPD-L1 levels (*R* = 0.871, *p* < 0.001) and miR-28-5p with CD8 + PD-1+ (*R* = 0.825, *p* < 0.001), indicating the involvement of these miRNAs in the activation of pathways that inhibit the function of CD8 + effector cells as a result of chronic immune stimulation. MiR-155-5p correlated positively with CD8 + PD-1+ (*R* = 0.857, *p* < 0.001), suggesting its association with the functional exhaustion of T lymphocytes in response to EBV reactivation. Significant intra-miRNA correlations, such as those between miR-155-5p and miR-28-5p (*R* = 0.854, *p* < 0.001) and between miR-326-5p and miRNA-142-5p (*R* = 0.636, *p* = 0.011), indicate the interdependent regulation of these molecules in the context of immune activation and disease progression. It is also worth noting the positive correlations between miR-221-5p and sCTLA-4 levels (*R* = 0.732, *p* = 0.002) and miR-326-5p and sCD200R (*R* = 0.736, *p* = 0.002), which underscore the involvement of these miRNAs in modulating immunosuppressive checkpoints (Supplementary Materials Table S11; Fig. 8B).

In the EBV- CLL group, particularly strong negative correlations were observed for miR-15a-5p and CD4 + CTLA-4+ (*R* = −0.757, *p* = 0.001) and miR-142-5p and CD8 + CTLA-4+ (*R* = −0.671, *p* = 0.006), suggesting that reduced levels of these miRNAs are associated with higher activity of the CTLA-4 pathway, which plays a key role in the induction of immunosuppression and functional exhaustion of T lymphocytes. In the case of miR-28-5p and CD4 + CD200R+ (*R* = −0.607, *p* = 0.016), significant correlations highlight its role in regulating the immunosuppressive CD200/CD200R pathway, which may influence the suppression of the immune response. In turn, positive correlations revealed strong associations between miRNAs and immunosuppressive and co-regulatory markers, particularly for miR-28-5p and miR-221-5p (*R* = 0.721, *p* = 0.002), suggesting a synergistic role in modulating immune processes. An exceptionally strong correlation was also noted between miR-181a-5p and miR-486-5p (*R* = 0.836, *p* < 0.001), highlighting their joint effect on regulating immune mechanisms. Moreover, the correlation between miR-33a-5p and CD19 + CD200R + (*R* = 0.829, *p* < 0.001) may indicate the importance of this miRNA in the immunosuppressive activity of B cells (Supplementary Materials Table S12; Fig. 8A).

ROC analysis confirms the high diagnostic value of immune markers (PD-1, PD-L1, CTLA-4, CD200, CD86) and their soluble forms in distinguishing patients with CLL from healthy volunteers. Selected miRNAs, such as miR-155-5p, miR-134a-5p, miR-29a-5p, miR-15a-5p, and miR-221-5p, also show diagnostic potential, but their efficiency is lower compared to cell markers and soluble checkpoints (Supplementary Materials Table S13). ROC curve analysis for miRNAs and immune markers reveals significant diagnostic differences between the HV, SID EBV+/-, and CLL EBV+/- groups. miR-16-5p shows the highest diagnostic efficiency in HV vs. SID EBV+ (AUC = 0.96, *p* < 0.0001) and HV vs. SID EBV- (AUC = 0.8667, *p* = 0.0006), confirming its role as a biomarker of EBV infection in SID. Its significance in CLL is limited (AUC ≤ 0.6933). miR-155-5p achieved excellent performance in differentiating SID EBV + from SID EBV- and CLL EBV+/- (AUC = 1.0, *p* < 0.0001), suggesting its usefulness in advanced diagnostics. miR-28-5p and miR-181a-5p showed high AUC values ​​(≥ 0.9644) in the comparisons of HV vs. SID EBV + and SID EBV + vs. CLL EBV+, emphasizing their potential in distinguishing EBV and CLL infections. miR-134-5p (AUC = 0.9867 for HV vs. SID EBV+, *p* < 0.0001) and miR-125b-5p (AUC = 0.9822 for HV vs. CLL EBV+, *p* < 0.0001) also demonstrate high diagnostic sensitivity. miR-142-5p reaches AUC = 1.0 for SID EBV + vs. SID EBV- and CLL EBV+, emphasizing its diagnostic versatility. Immune markers, such as CD4 + PD-1+, CD8 + PD-L1+, sPD-1, sPD-L1, sCTLA-4, and sCD86, show an AUC of 1.0 in most comparisons, confirming their exceptional performance in differentiating groups and monitoring the immune response. In particular, PD-1 and CTLA-4-related cellular markers suggest a significant role for immunoregulatory pathways in the context of EBV and CLL (Supplementary Materials, Table S14).

## Discussion

Our studies have provided new insights into the role of miRNAs and immune checkpoints in the pathogenesis and progression of CLL, with particular emphasis on the impact of EBV reactivation and SID. Detailed analysis of patient subgroups, including SID and EBV reactivation, allowed us to link differences in miRNA expression, cell phenotype, and immune checkpoint activity to specific clinical conditions.

Research studies have shown significant upregulation of miR-15a-5p, miR-155-5p, and miR-134-5p in CLL samples compared with healthy controls [8,21,63,]. Overexpression of miR-155-5p is a key immunosuppressive factor because it suppresses T cell effector functions and promotes PD-L1 expression, leading to immune evasion by cancer cells^21,62,63^. These changes suggest a significant role of miR-155-5p deregulation in forming an immunosuppressive microenvironment and disease progression. On the other hand, the decreased expression of miR-181a and miR-181b in CLL compared to normal B lymphocytes^3,64^ indicates the loss of miRNAs with suppressor properties. These miRNAs target the PI3 K pathway, which is involved in apoptosis defects in CLL cells, and their downregulation may promote the survival and expansion of these cells^3^. Additionally, specific miRNA signatures, such as the decreased expression of miR-223, miR-29a, and miR-181, are associated with prognostic factors and high-risk cytogenetic subgroups, highlighting their clinical importance in risk stratification^30^. For example, upregulation of miR-155 is a marker of monoclonal B-cell lymphocytosis, which can progress to overt CLL^62^.

In our study’s context, the analysis showed that miRNA levels differ depending on the presence of secondary immunodeficiencies (SID) and EBV reactivation. It was observed that miR-15a, miR-16, miR-28, miR-142, miR-150, miR-155, miR-181a, miR-326, and miR-486 values ​​are the highest in patients with CLL EBV−, then decrease in the CLL EBV + group, are even lower in the SID EBV − group and reach the lowest values ​​in the SID EBV + group. The high levels of these miRNAs in the EBV − CLL group may be due to their role in the pathogenesis of chronic lymphocytic leukemia, where miRNAs such as miR-15a and miR-16 act as tumor suppressors by controlling the expression of anti-apoptotic proteins such as BCL2 and by inhibiting uncontrolled cell proliferation. Similarly, miR-155 and miR-150, which regulate B cell activation and differentiation, are overexpressed in CLL, promoting tumor cell survival and resistance to apoptosis. In the EBV + CLL group, the levels of miRNAs are reduced, which may be due to the reactivation of EBV, which encodes its miRNAs and interferes with regulatory, economic pathways. EBV virus can inhibit immune functions and deregulate suppressor miRNAs, promoting disease progression and immunosuppression. Further reduction in the levels of the analyzed miRNAs observed in the groups with secondary immunodeficiencies (SID EBV − and SID EBV+) is probably related to the deepening exhaustion of the immune system function. In the SID EBV − group, the decrease in miRNAs may be due to systemic immunosuppression and lymphocyte exhaustion, leading to reduced miRNA production and destabilization. In turn, the lowest miRNA levels in the SID EBV + group reflect the synergistic effect of secondary immunodeficiencies and EBV virus reactivation. EBV reactivation leads to profound deregulation of immune functions, which includes activation of immunosuppressive pathways, such as PD-1/PD-L1 or CTLA-4, and functional exhaustion of T and B lymphocytes. Moreover, EBV can inhibit the transcription of economic miRNA, enhancing immunosuppression and attenuating the antiviral response. Some of the analyzed miRNAs, such as miR-142 and miR-150, play a key role in the differentiation and activation of T lymphocytes and NK cells, which are essential for eliminating EBV-infected cells. Their decreased levels may indicate exhaustion of these cells and attenuation of the antiviral response. In turn, miR-155 and miR-181a are responsible for the inflammatory response and activation of B lymphocytes and macrophages, and their low levels in EBV + SID suggest profound immunosuppression and viral interference. Decreased levels of miR-326 and miR-486, which regulate innate and adaptive immunity, lead to further attenuation of the immune response.

Dysregulation of checkpoint pathways such as PD-1/PD-L1, CTLA-4/CD86, and CD200/CD200R play a key role in immunosuppression in CLL. Increased expression of PD-L1 on CLL B cells and PD-1 on CD4 + and CD8 + T cells results in attenuation of T cell activation, leading to T cell anergy and promoting the creation of an immunosuppressive microenvironment^65–67^. Furthermore, PD-L1 expression can be enhanced by EBV infection^66^. The CTLA-4/CD86 axis also plays an important role, as CTLA-4 competes with CD28 for binding to the CD86 ligand, impairing T-cell activation and proliferation^68^. In turn, overexpression of CD200R on B cells and CD200 on T cells indicates further mechanisms promoting immune tolerance and evasion of immune surveillance by tumor cells^69^.

Our study further showed that the highest levels of PD-1, PD-L1, and CTLA-4 on CD4+, CD8+, and CD19 + lymphocytes were found in the SID EBV + and CLL EBV + groups, indicating advanced immunosuppression resulting from EBV reactivation (Fig. 5). A similar pattern was observed for the markers CD200 and CD200R, which were most elevated in the SID EBV + group, emphasizing their role in suppressing the immune response. These results suggest that the activation of these pathways is particularly enhanced in the presence of EBV reactivation and secondary immunodeficiencies. The profile of miRNA deregulators and checkpoint molecules suggests significant clinical possibilities. MiRNAs, such as miR-155-5p, may act as prognostic biomarkers, and their modulation may provide the basis for new therapeutic strategies^62,65^. Similarly, targeting the PD-1/PD-L1, CTLA-4/CD86, and CD200/CD200R checkpoint pathways with immune inhibitors can potentially restore antitumor immunity^70,71^.

Our results also indicate the high diagnostic value of selected miRNAs (e.g., miR-155-5p, miR-29a) and immunosuppressive markers (PD-1, PD-L1, CTLA-4) in differentiating patients with CLL and SID and in assessing EBV reactivation (Supplementary Materials Table S13, S14). The high sensitivity and specificity of immune markers confirm their usefulness in diagnostics and monitoring therapeutic responses. In addition to the well-studied PD-1/PD-L1 and CTLA-4 pathways, other checkpoint molecules such as LAG-3 or CD73 also play a potential role and may constitute additional therapeutic targets^25^.

Although the presented studies show statistically significant results of analyses, our study also encountered several limitations. The limitations of the conducted study result mainly from its complex nature and methodological limitations. First, the number of patients in the groups, especially in the context of division into subgroups depending on the presence of SID and EBV reactivation, was relatively small, which may limit the possibility of generalizing the results to a broader population. Second, the analysis of miRNA and immune checkpoint expression was performed based on serum and peripheral blood samples, which may not fully reflect the changes occurring in the bone marrow microenvironment or in lymph nodes, which are key in the pathogenesis of CLL. Moreover, the analysis of the relationship between miRNA levels, EBV markers and immunosuppression parameters was correlational, which does not allow for a precise determination of cause-effect relationships.

A significant limitation of this study is the use of only the PBMC fraction to determine the number of EBV DNA copies. Although this choice was based on the study design, which also analyzed the immunophenotype of cells and expression of checkpoints, it is worth noting that the determination of EBV DNA in plasma is considered a more sensitive and preferred marker of active viremia. Parallel determination of the number of EBV copies in plasma could provide a more comprehensive picture of the virus’s activity in the organism and increase the interpretive value of the obtained results. The inclusion of both fractions (PBMC and plasma) is planned in the future stages of the project.

An additional limitation of the study is the lack of available data on the mutation status in the *TP53* gene and other important genetic markers (e.g., *del17p*,* NOTCH1 mutations*,* SF3B1*), which may affect both the degree of immunosuppression and susceptibility to EBV reactivation in patients with CLL. Their inclusion could facilitate a more in-depth interpretation of the obtained results and the identification of potential relationships between molecular determinants and immunological profiles.

Finally, a limitation of the study is its retrospective nature, which does not allow for a full consideration of dynamic changes related to disease progression and treatment response over time. Future studies would be encouraged to increase the sample size, include more representative tissue samples, and use a prospective approach to better understand the mechanisms underlying the observed changes.

## Materials

### Patients

A total of 75 participants were included in the study, divided into three groups: 30 patients with chronic lymphocytic leukemia (CLL) (15 patients with EBV reactivation, and 15 patient without EBV reactivation), 30 patients with CLL and secondary immunodeficiencies (SID) (15 patients with EBV reactivation, and 15 patient without EBV reactivation), and 15 healthy volunteers (HV) serving as the control group (all patient without EBV reactivation). All participants met the specified inclusion and exclusion criteria. Patients with CLL and SID were newly diagnosed. Participant selection was conducted by an experienced clinical immunologist using precise criteria. Inclusion criteria included age ≥ 18 years, life expectancy ≥ 12 months, no immunosuppressive therapy within three months before study entry, and written informed consent. Exclusion criteria included active viral, bacterial, or fungal infection; severe allergies; previous hematopoietic cell or organ transplantation; active malignancies or autoimmune diseases during treatment; pregnancy or lactation; participation in clinical trials for new drugs; presence of metastases in the central nervous system; and significant mental disorders. All patients included in the analysis were in Rai stage 1 and Binnet stage A, and biological material (serum and peripheral blood mononuclear cells) was collected after diagnosis by a clinical immunology specialist; however, we did not have data on IGHV mutation status.

SID in patients with CLL is defined as a state of impaired immune response resulting from the underlying disease and/or its treatment, leading to increased susceptibility to infections. The main criteria include reduced immunoglobulin levels (e.g. IgG < 5 g/L), impaired humoral response despite normal antibody levels, recurrent infections (especially respiratory infections), impaired post-vaccination response, and deficits in T-cell and antigen-presenting cell function. According to current guidelines, the clinical criterion of SID may also be the occurrence of ≥ 8 infectious episodes requiring antibiotic therapy in the 12 months preceding diagnosis^25^.

The research material included 10 mL of peripheral blood collected from the basilic vein into EDTA tubes (for immunophenotypic analysis) and 5 mL of blood into clot tubes (to obtain serum for miRNA assays and soluble forms of the studied molecules). All participants provided informed consent, willingly agreeing to take part in the study after receiving detailed information about its purpose and procedures, and all methods were carried out in full compliance with relevant guidelines and regulations to ensure adherence to ethical standards. The study was approved by Bioethics Committee of the Medical University of Lublin (KE-0254/247/2023).

## Immunophenotyping

To assess the expression of PD-1, PD-L1, CTLA4, CD80, CD200, CD200R on CD3+/CD4+, CD3+/CD8 + and CD3-/CD19 + lymphocytes. Peripheral blood samples from study participants were incubated with a panel of monoclonal anti-human antibodies, including CD45 FITC, CD3 BV510, CD4 BV650, CD8 BV605, CD19 BB700, PD-1 PE, PD-L1 BV421, CTLA-4 PE, CD80 PE-Cy7 and CD200 APC, CD200R BV421 (BD, Franklin Lakes, NJ, USA). To enhance the stability of the antibodies, assessed using a violet laser, BD Horizon™ Brilliant Stain Buffer (BD, Franklin Lakes, NJ, USA) was used. Erythrocytes were removed with lysis buffer (BD, Franklin Lakes, NJ, USA), and cells were washed with BD Pharmingen™ Stain Buffer (BSA) (BD, Franklin Lakes, NJ, USA). Analyses were performed on a CytoFLEX LX flow cytometer (Beckman Coulter, Indianapolis, IN, USA), equipped with daily quality control using CytoFLEX Daily QC Fluorosphere reagents to minimize instrument-related variability. Data were analyzed using Kaluza Analysis software version 2.1 (Beckman Coulter, Indianapolis, IN, USA).

### Serological profiling of Anti-EBV antibodies

To analyze the presence of anti-EBV antibodies, we performed a qualitative assessment targeting specific IgA, IgM, and IgG antibodies against Epstein–Barr virus (EBV) antigens, including viral capsid antigen (VCA), early antigen (EA), and Epstein–Barr nuclear antigen 1 (EBNA1). Commercial ELISA kits were employed to detect antibodies of these classes. The assays adhered strictly to the manufacturer’s protocols. The kits utilized were: EBV VCA IgA, EBV EA IgA, EBV EBNA1 IgA, EBV VCA IgG, EBV EA IgG, EBV EBNA1 IgG, EBV VCA IgM, EBV EA IgM, and EBV EBNA1 IgM (Demeditec Diagnostics GmbH, Kiel, Germany). Absorbance measurements were taken using a Victor™3 microplate reader (PerkinElmer, Waltham, MA, USA). Antibody titers were quantified in U/mL, following the calibration standards provided by the manufacturer. A titer exceeding 11 U/mL was classified as positive.

## Quantification of EBV genome copies in PBMC DNA

Quantification of EBV genome copies was performed using the ISEX EBV GeneProof PCR kit (Brno, Czech Republic). All samples were analyzed in duplicates, including a negative control containing DNA elution buffer. The amplification reaction was targeted to a conserved DNA sequence specific for the EBV EBNA1 gene using the 7300 Real-Time PCR system (Applied Biosystems, Foster City, CA, USA) according to the ISEX kit protocol. The final viral DNA concentration was expressed as copies per microgram of isolated DNA and normalized to the DNA isolation efficiency. The detection threshold was set at 10 EBV DNA copies/µl, and samples with lower copy levels were considered EBV negative.

## Assessment of soluble immune checkpoint and ligand levels

Serum concentrations of soluble immune checkpoints and their ligands were quantified using immunoenzymatic assays on collected serum samples. Commercial ELISA kits with the following sensitivities were used: human CD200 (20 pg/mL, Invitrogen, Waltham, MA, USA), CD200R (11.89 pg/mL, Abcam, Cambridge, UK), CTLA-4 (0.13 ng/mL, Invitrogen), CD86 (0.82 ng/mL, Invitrogen), PD-1 (1.14 pg/mL, Invitrogen), and PD-L1 (0.6 pg/mL, Invitrogen). The assays were performed using a Victor™3 reader, following the manufacturers’ instructions. Concentrations were reported as per kit specifications to ensure consistency and reliability.

### MiRNA isolation from patient plasma

MicroRNA isolation from serum or plasma samples was performed using the miRNeasy Serum/Plasma Advanced Kit (Qiagen) according to the manufacturer’s protocol. For isolation, 200 µl of samples were used, which were first lysed in the presence of 400 µl of QIAzol Lysis Reagent buffer by vortexing for 1 min. Samples were incubated at room temperature for 5 min, then 90 µl of chloroform was added, mixed vigorously for 15 s and left for 3 min at room temperature. After incubation, samples were centrifuged at 12,000 x g for 15 min at 4 °C to separate the phases. The aqueous phase containing RNA was carefully transferred to a new tube, to which 1.5 volumes of ethanol (96–100%) was added. The mixture was then transferred to an RNeasy Mini Spin Column, where the RNA was bound to the membrane. The column was washed twice with RWT and RPE buffers per the kit instructions to remove any remaining contaminants. The RNA was eluted from the membrane using 30 µl of RNase-free water by incubation for 1 min, followed by centrifugation. The resulting RNA was stored at −80 °C until further analysis.

### cDNA synthesis by reverse transcription

According to the manufacturer’s instructions, the isolated RNA was reverse transcribed into cDNA using the miRCURY LNA RT Kit (Qiagen). The procedure included the following steps: Preparation of the reaction mixture was started by mixing RNA in a volume of 10 µl with 2 µl of miRCURY RT Synth Primer Mix primers and 4 µl of miRCURY RT Reaction Buffer in a tube. Then, 4 µl of miRCURY RT Enzyme Mix was added to the tube. The total reaction volume of 20 µl was mixed thoroughly by gentle pipetting. The tubes were incubated in a thermocycler under the following conditions: 60 min at 42 °C for reverse transcription, after which the reaction was stopped by 5 min incubation at 95 °C. The resulting cDNA was diluted 1:10 with RNase-free water and stored at −80 °C until further analysis.

### Digital PCR (dPCR)

Digital PCR (dPCR) was used for quantitative analysis of cDNA using the QIAcuity EG PCR Kit (Qiagen) and EvaGreen dye. Preparation of the reaction mixture included mixing 4 µl of 3x EvaGreen PCR Master Mix, 1.2 µl of 10x primer mix (0.4 µM each primer), RNase-free water, and up to 5 µl of cDNA template, so that the total reaction volume was 12 µl per sample. hsa-miR-155-5p (YP02119311); hsa-miR-210-5p (YP02104321); hsa-miR-181a-5p (YP00206081); hsa-miR-134-5p (YP00205989); hsa-miR-125b-5p (YP00205713); hsa-miR16-5p (YP002055702);hsa-miR-33a-5p (YP00205690); hsa-miR-30c-5p (YP00204783); hsa-miR-142-5p (YP00204722); hsa-miR-144-59(YP00204670); hsa-miR-744-5p (YP00204663); hsa-miR-150-5p (YP00204660); hsa-miR-326 (YP00204512); hsa-miR-29a-5p (YP00204430); hsa-miR-28-5p (YP00204322); hsa-miR-21-5p (YP00204230); hsa-miR-15a-5p (YP00204066); hsa-miR-221-54p (YP00204032); hsa-miR486-5p (YP00204001) (Qiagen). Samples were transferred to a standard PCR plate, and then the contents of each well were transferred to the wells of a QIAcuity nanoplate. After sealing the nanoplate with a dedicated seal, amplification was performed in a thermal cycler under the following conditions: polymerase activation at 95 °C for 2 min, denaturation at 95 °C for 30 s, annealing and elongation at 60 °C for 1 min, repeated for 40 cycles. After amplification, the samples were cooled at 40 °C for 5 min. The nanoplate was analyzed on the QIAcuity platform, which enabled the detection of the fluorescent signal emitted by the EvaGreen dye binding to double-stranded DNA. Data analysis was performed using QIAcuity Suite software, which determined the number of copies of the target miRNA per microliter of the sample.

### Statistical analysis

Data were statistically analyzed using Statistica and GraphPad Prism. The normality of distribution was assessed using the Shapiro-Wilk test. In the case of data not meeting the assumptions of normality, nonparametric tests were used, such as the Mann-Whitney U test for comparisons of two groups or the Kruskal-Wallis test for a larger number of groups, with Bonferroni correction in multiple analyses. Correlation analyses were performed using the Spearman correlation coefficient. Results were considered statistically significant at *p* < 0.05.

## Conclusion

In summary, the study provided important data on the role of miRNAs and immune checkpoints in the pathogenesis and progression of CLL, especially in the context of SID and EBV reactivation. It was shown that deregulation of miRNAs, including miR-155-5p, miR-21 and miR-29a, plays a key role in the modulation of immunosuppression mechanisms, and its intensity is particularly visible in patients with EBV reactivation. Increased expression of immune checkpoints, such as PD-1/PD-L1, CTLA-4/CD86 or CD200/CD200R, has been associated with exacerbation of immunosuppression and disease progression, which underlines their importance as potential therapeutic targets. The study revealed complex relationships between miRNAs, EBV response, and immune system function, indicating their diagnostic and prognostic value. At the same time, it has been shown that patients with SID, especially those with concomitant EBV reactivation, are more susceptible to infections and immunosuppression, which may affect their prognosis and the course of CLL. These results emphasize the need for further studies that will allow for a better understanding of molecular mechanisms and assessment of the efficacy of therapeutic targeting of miRNAs and checkpoint pathways. In the future, the use of larger study cohorts, prospective design, and extension of analyses to tissue samples and dynamics of changes over time may provide additional data and contribute to the development of more effective diagnostic and therapeutic strategies in the treatment of CLL.

### Data Availability

Data is provided within the manuscript or supplementary information files.

## Electronic supplementary material

Below is the link to the electronic supplementary material.

Supplementary Material 1
