# Supplementary material for: MicroRNA changes with macro potential contribute to secondary immunodeficiency in chronic lymphocytic leukemia during epstein barr virus reactivation
Source: Sci Rep. 2025 May 12;15:16446. doi: 10.1038/s41598-025-01572-4 (PMC12069660; doi:10.1038/s41598-025-01572-4)
Supplement: Supplementary file 1 — Supplementary Material 1 [file 41598_2025_1572_MOESM1_ESM.docx]

**Supplementary Materials:**

**Tabel S1:** Statistical analyses of miRNA between CLL patients (without taking into account the presence of immunodeficiencies) and healthy volunteers

| **Parameters** | **All CLL Patients** | | **Healthy Volunteers (HV)** | | **p-Value** |
| --- | --- | --- | --- | --- | --- |
|  | **Median** | **Q1-Q3** | **Median** | **Q1-Q3** |  |
| miR16-5p | 7584.28 | 3920.79-11663.64 | 9780.64 | 7285.93-12520.23 | 0.075 |
| miR155-5p | 89.54 | 20.80-388.58 | 11.72 | 9.65-36.80 | 0.002* |
| miR30c-5p | 692.40 | 180.90-1369.00 | 1144.46 | 727.72-1497.81 | 0.096 |
| miR28-5p | 47.35 | 12.60-191.78 | 134.40 | 48.48-406.58 | 0.066 |
| miR210-5p | 0.00 | 0.00-0.00 | 0.00 | 0.00-0.00 | NA |
| miR181a-5p | 419.28 | 100.85-1300.92 | 1046.17 | 334.37-1721.76 | 0.102 |
| miR134-5p | 86.64 | 49.84-131.39 | 133.82 | 109.18-182.19 | 0.004* |
| miR125b-5p | 152.19 | 44.07-323.04 | 76.35 | 50.66-139.65 | 0.108 |
| miR33a-5p | 76.61 | 39.17-170.31 | 77.59 | 67.29-198.09 | 0.213 |
| miR142-5p | 548.60 | 205.04-1458.31 | 821.93 | 556.92-1387.33 | 0.105 |
| miR144-5p | 5.25 | 0.00-7.73 | 3.45 | 2.33-6.03 | 0.819 |
| miR744-5p | 622.36 | 252.30-1185.26 | 702.27 | 533.08-1054.46 | 0.450 |
| miR150-5p | 4389.57 | 1471.90-8658.86 | 8337.29 | 3665.89-9781.25 | 0.194 |
| miR326-5p | 283.47 | 96.49-981.40 | 626.40 | 293.39-2239.69 | 0.059 |
| miR29a-5p | 5.88 | 4.02-13.31 | 16.41 | 6.76-18.77 | 0.009* |
| miR21-5p | 89.76 | 0.44-856.17 | 75.90 | 0.00-849.55 | 0.568 |
| miR15a-5p | 333.99 | 128.16-789.83 | 800.11 | 397.80-1206.13 | 0.014* |
| miR221-5p | 1.79 | 0.63-7.99 | 0.66 | 0.16-1.37 | 0.016* |
| miR486-5p | 2532.61 | 1282.98-5403.56 | 3282.33 | 1749.17-5905.25 | 0.321 |

**Tabel S2:** Statistical analyses of EBV reactivation between CLL patients (without taking into account the occurrence of immunodeficiencies) and healthy volunteers

| **Parameters** | **All CLL Patients** | | **Healthy Volunteers (HV)** | | **p-Value** |
| --- | --- | --- | --- | --- | --- |
|  | **Median** | **Q1-Q3** | **Median** | **Q1-Q3** |  |
| EA IgA | 22.59 | 3.41-61.86 | 5.40 | 2.77-6.86 | 0.156 |
| EA IgM | 5.16 | 3.96-6.55 | 4.82 | 3.87-6.12 | 0.568 |
| EA IgG | 35.37 | 5.07-89.38 | 3.27 | 2.89-4.96 | 0.000* |
| VCA IgA | 11.08 | 6.34-23.11 | 4.86 | 3.94-5.46 | 0.000* |
| VCA IgM | 20.66 | 5.95-44.31 | 5.98 | 3.78-7.26 | 0.001* |
| VCA IgG | 190.00 | 131.89-221.70 | 118.14 | 87.47-126.19 | 0.000* |
| EBNA IgA | 9.48 | 4.99-16.32 | 2.96 | 2.22-4.83 | 0.000* |
| EBNA IgM | 6.73 | 5.88-7.84 | 4.58 | 3.81-6.22 | 0.000* |
| EBNA IgG | 136.74 | 57.78-249.15 | 58.00 | 52.62-70.67 | 0.009* |
| EBV copy number/µg DNA | 338.18 | 0.00-801.01 | 0.00 | 0.00-0.00 | 0.002* |

**Tabel S3:** Statistical analyses of peripheral blood immunophenotype with special emphasis on the percentage of selected immune checkpoints and their ligands between patients with CLL (without taking into account the occurrence of immunodeficiencies) and healthy volunteers

| **Parameters** | **All CLL Patients** | | **Healthy Volunteers (HV)** | | **p-Value** |
| --- | --- | --- | --- | --- | --- |
|  | **Median** | **Q1-Q3** | **Median** | **Q1-Q3** |  |
| CD45+ [%] | 90.94 | 88.54-95.35 | 93.63 | 91.22-95.04 | 0.052 |
| CD3+ [%] | 18.95 | 14.65-24.47 | 74.14 | 69.12-84.63 | 0.000* |
| T CD4+ lymphocytes[%] | 10.38 | 6.93-13.75 | 48.08 | 46.51-53.67 | 0.000* |
| T CD8+ lymphocytes [%] | 9.71 | 6.19-16.07 | 39.06 | 27.53-43.63 | 0.000* |
| B CD19+ lymphocyte s[%] | 65.57 | 56.86-73.38 | 12.79 | 8.22-13.88 | 0.000* |
| Ratio T CD4/ T CD8+ lymphocytes | 1.06 | 0.52-1.67 | 1.28 | 1.16-1.95 | 0.040* |
| CD4+PD-1+ [%] | 18.23 | 13.55-34.72 | 3.54 | 2.31-4.53 | 0.000* |
| CD8+PD-1+ [%] | 13.77 | 7.61-24.56 | 3.63 | 2.45-4.31 | 0.000* |
| CD19+PD-1+[%] | 15.85 | 9.67-25.84 | 3.96 | 2.65-4.70 | 0.000* |
| CD4+PD-L1+[%] | 10.69 | 6.48-14.83 | 0.73 | 0.27-1.00 | 0.000* |
| CD8+PD-L1+[%] | 9.53 | 4.31-15.79 | 0.51 | 0.37-0.70 | 0.000* |
| CD19+PD-L1+[%] | 12.46 | 6.32-17.07 | 0.67 | 0.28-1.40 | 0.000* |
| CD4+CTLA-4+[%] | 13.61 | 7.02-24.68 | 3.10 | 2.54-3.75 | 0.000* |
| CD8+CTLA-4+[%] | 15.51 | 9.88-23.86 | 3.20 | 2.93-4.26 | 0.000* |
| CD19+CTLA-4+[%] | 4.45 | 1.89-8.44 | 2.13 | 1.90-2.55 | 0.006* |
| CD4+CD86+[%] | 6.50 | 5.04-10.36 | 2.81 | 2.55-3.63 | 0.000* |
| CD8+CD86+[%] | 4.95 | 3.98-8.17 | 1.97 | 1.40-2.78 | 0.000* |
| CD19+CD86+[%] | 39.08 | 30.72-51.38 | 12.08 | 9.35-16.11 | 0.000* |
| CD4+CD200R+[%] | 10.67 | 5.86-17.44 | 3.61 | 2.36-6.24 | 0.000* |
| CD8+CD200R+[%] | 10.71 | 6.37-20.44 | 5.34 | 2.31-5.62 | 0.000* |
| CD19+CD200R+[%] | 13.03 | 8.30-23.27 | 23.06 | 18.23-25.27 | 0.005* |
| CD4+CD200+[%] | 21.04 | 8.67-36.68 | 3.03 | 1.85-3.48 | 0.000* |
| CD8+200+[%] | 16.96 | 9.52-27.11 | 3.79 | 2.55-4.68 | 0.000* |
| CD19+CD200+ [%] | 77.15 | 66.79-91.70 | 42.22 | 31.93-54.43 | 0.000* |

**Tabel S4:** Statistical analyses of serum concentrations of soluble forms of selected immune checkpoints and their ligands between patients with CLL (without taking into account the presence of immunodeficiencies) and healthy volunteers

| **Parameters** | **All CLL Patients** | | **Healthy Volunteers (HV)** | | **p-Value** |
| --- | --- | --- | --- | --- | --- |
|  | **Median** | **Q1-Q3** | **Median** | **Q1-Q3** |  |
| sPD-1 [ng/ml] | 41.29 | 34.00-52.91 | 2.55 | 1.55-4.10 | 0.000* |
| sPD-L1 [ng/ml] | 26.32 | 22.18-34.53 | 1.75 | 0.83-2.07 | 0.000* |
| sCTLA-4 [ng/ml] | 24.64 | 17.56-27.72 | 3.12 | 2.42-4.35 | 0.000* |
| sCD86 [ng/ml] | 19.43 | 16.87-23.69 | 1.75 | 1.53-2.59 | 0.000* |
| sCD200R [ng/ml] | 31.84 | 27.02-40.05 | 4.57 | 2.52-6.15 | 0.000* |
| sCD200 [ng/ml] | 40.00 | 32.36-54.71 | 2.17 | 1.50-4.08 | 0.000* |

**Tabel S5:** Statistical analyses of EBV reactivation between SID and CLL and healthy volunteers including the status of EBV reactivation

| **Parameters** | **SID EBV+**  **(group 3)** | **SID EBV-**  **(group 4)** | **CLL EBV+**  **(group 5)** | **CLL EBV-**  **(group 6)** | **HV**  **(group 2)** | **p-Value** | | | | | | | | | |
| --- | --- | --- | --- | --- | --- | --- | --- | --- | --- | --- | --- | --- | --- | --- | --- |
|  | Median (Q1-Q3) | Median (Q1-Q3) | Median (Q1-Q3) | Median (Q1-Q3) | Median (Q1-Q3) | 3 vs 4 | 3 vs. 5 | 3 vs. 6 | 3 vs. 2 | 4 vs. 5 | 4 vs. 6 | 4 vs. 2 | 5 vs. 6 | 5 vs. 2 | 6 vs. 2 |
| EA IgA | 57.72  (48.55-62.62) | 3.23  (1.73-4.04) | 64.41  (49.05-71.27) | 3.58  (2.17-4.71) | 5.40  (2.77-6.86) | 0.000* | 0.056 | 0.000* | 0.000* | 0.000* | 0.595 | 0.010* | 0.000* | 0.000* | 0.021* |
| EA IgM | 5.93  (3.68-8.55) | 5.05  (3.61-6.35) | 5.62  (4.02-7.41) | 5.05  (4.06-6.38) | 4.82  (3.87-6.12) | 0.098 | 0.367 | 0.217 | 0.250 | 0.389 | 0.683 | 0.744 | 0.595 | 0.461 | 0.806 |
| EA IgG | 88.61  (69.49-106.83) | 5.17  (4.33-6.65) | 90.15  (83.81-107.76) | 4.75  (4.17-6.55) | 3.27  (2.89-4.96) | 0.000* | 0.539 | 0.000* | 0.000* | 0.000* | 0.624 | 0.002* | 0.000* | 0.000* | 0.004* |
| VCA IgA | 23.75  (19.09-27.92) | 6.44  (4.46-7.34) | 22.73  (15.86-28.30) | 5.98  (4.03-6.76) | 4.86  (3.94-5.46) | 0.000* | 0.595 | 0.000* | 0.000* | 0.000* | 0.345 | 0.050 | 0.000* | 0.000* | 0.267 |
| VCA IgM | 46.10  (38.77-53.13) | 6.64  (5.86-7.33) | 44.12  (40.44-52.31) | 4.73  (4.34-6.97) | 5.98  (3.78-7.26) | 0.000* | 1.000 | 0.000* | 0.000* | 0.000* | 0.106 | 0.412 | 0.000* | 0.000* | 0.838 |
| VCA IgG | 234.23  (190.22-247.19) | 143.71  (105.17-173.19) | 217.61  (198.85-237.21) | 116.92  (105.17-165.82) | 118.14  (87.47-126.19) | 0.000* | 0.683 | 0.000* | 0.000* | 0.000* | 0.935 | 0.009* | 0.000* | 0.000* | 0.056 |
| EBNA IgA | 16.59  (14.56-18.04) | 5.39  (4.75-5.75) | 16.20  (14.48-16.79) | 4.75  (4.49-5.39) | 2.96  (2.22-4.83) | 0.000* | 0.367 | 0.000* | 0.000* | 0.000* | 0.126 | 0.000* | 0.000* | 0.000* | 0.004* |
| EBNA IgM | 8.11  (6.70-9.28) | 5.88  (5.55-6.69) | 6.86  (6.24-8.13) | 6.60  (5.83-7.36) | 4.58  (3.81-6.22) | 0.000* | 0.233 | 0.003* | 0.000* | 0.037* | 0.325 | 0.041* | 0.233 | 0.000* | 0.004* |
| EBNA IgG | 232.46  (208.28-260.78) | 55.79  (45.83-72.02) | 255.02  (224.67-268.24) | 64.50  (45.97-72.02) | 58.00  (52.62-70.67) | 0.000* | 0.305 | 0.000* | 0.000* | 0.000* | 0.775 | 0.389 | 0.000* | 0.000* | 0.713 |
| EBV copy number/µg DNA | 812.60  (791.26-860.28) | NA | 779.73  (729.95-802.73) | NA | NA | 0.000* | 0.045 | 0.000* | 0.000* | 0.000* | NA | NA | 0.000* | 0.000* | NA |

Legend: Group 2 – healthy volunteers (HV), Group 3 – patients with secondary immunodeficiency and EBV reactivation (SID EBV⁺), Group 4 – patients with secondary immunodeficiency without EBV reactivation (SID EBV⁻), Group 5 – patients with chronic lymphocytic leukemia and EBV reactivation (CLL EBV⁺), Group 6 – patients with chronic lymphocytic leukemia without EBV reactivation (CLL EBV⁻). Statistically significant results are marked with *.

**Tabel S6:** Statistical analyses of miRNA between SID and CLL and healthy volunteers including the status of EBV reactivation

| **Parameters** | **SID EBV+**  **(group 3)** | **SID EBV-**  **(group 4)** | **CLL EBV+**  **(group 5)** | **CLL EBV-**  **(group 6)** | **HV**  **(group 2)** | **p-Value** | | | | | | | | | |
| --- | --- | --- | --- | --- | --- | --- | --- | --- | --- | --- | --- | --- | --- | --- | --- |
|  | **Median (Q1-Q3)** | **Median (Q1-Q3)** | **Median (Q1-Q3)** | **Median (Q1-Q3)** | **Median (Q1-Q3)** | 3 vs 4 | 3 vs. 5 | 3 vs. 6 | 3 vs. 2 | 4 vs. 5 | 4 vs. 6 | 4 vs. 2 | 5 vs. 6 | 5 vs. 2 | 6 vs. 2 |
| miR16-5p | 3083.36  (2305.87-5884.33) | 5236.65  (1125.20-7709.16) | 10168.29  (7910.39-19334.60) | 11944.20  (9360.70-17446.05) | 9780.64  (7285.93-12520.23) | 0.233 | 0.000* | 0.000* | 0.000* | 0.000* | 0.000* | 0.000* | 0.233 | 0.775 | 0.074 |
| miR155-5p | 9.360  (5.69-11.66) | 38.35  (29.25-46.68) | 212.05  (183.12-309.00) | 651.43  (452.30-919.32) | 11.72  (9.65-36.80) | 0.000* | 0.000* | 0.000* | 0.050 | 0.000* | 0.000* | 0.026* | 0.000* | 0.000* | 0.000* |
| miR30c-5p | 116.27  (58.27-188.26) | 661.09  (134.70-1048.48) | 927.25  (612.49-1809.10) | 1562.41  (1126.53-1758.00) | 1144.46  (727.72-1497.81) | 0.007* | 0.000* | 0.000* | 0.000* | 0.074 | 0.000* | 0.013* | 0.067 | 0.683 | 0.041* |
| miR28-5p | 7.74  (6.32-13.36) | 36.90  (30.88-44.88) | 132.73  (113.30-189.76) | 721.83  (207.49-782.76) | 134.40  (48.48-406.58) | 0.007* | 0.000* | 0.000* | 0.000* | 0.000* | 0.000* | 0.000* | 0.002* | 1.000 | 0.019* |
| miR210-5p | 0.00  (0.00-0.00) | 0.00  (0.00-0.00) | 0.00  (0.00-0.00) | 0.00  (0.00-0.00) | 0.00  (0.00-0.00) | NA | | | | | | | | | |
| miR181a-5p | 69.30  (36.55-89.60) | 186.70  (112.09-296.13) | 1173.45  (988.00-1289.39) | 1558.63  (1312.44-2059.39) | 1046.17  (334.37-1721.76) | 0.005* | 0.000* | 0.000* | 0.000* | 0.000* | 0.000* | 0.000* | 0.021* | 0.305 | 0.016* |
| miR134-5p | 75.61  (38.76-95.67) | 84.34  (55.59-144.37) | 86.57  (77.58-105.35) | 179.70  (126.77-201.97) | 133.82  (109.18-182.19) | 0.137 | 0.106 | 0.000* | 0.000* | 0.935 | 0.021* | 0.033* | 0.006* | 0.000* | 0.217 |
| miR125b-5p | 117.69  (43.69-177.00) | 25.36  (23.85-80.13) | 416.21  (242.60-735.58) | 241.30  (62.06-431.72) | 76.35  (50.66-139.65) | 0.019* | 0.000* | 0.019* | 0.512 | 0.000* | 0.000* | 0.009* | 0.023* | 0.000* | 0.013* |
| miR33a-5p | 58.89  (42.04-75.24) | 31.28  (11.29-51.97) | 219.28  (165.32-334.80) | 90.82  (67.75-153.55) | 77.59  (67.29-198.09) | 0.106 | 0.000* | 0.033* | 0.010* | 0.000* | 0.000* | 0.000* | 0.000* | 0.010* | 0.870 |
| miR142-5p | 209.78  (79.35-341.80) | 209.80  (166.76-471.16) | 1438.94  (818.30-2228.18) | 1494.10  (796.79-1849.69) | 821.93  (556.92-1387.33) | 0.567 | 0.000* | 0.000* | 0.000* | 0.000* | 0.000* | 0.000* | 0.567 | 0.045* | 0.202 |
| miR144-5p | 2.21  (0.00-6.03) | 7.47  (6.02-8.51) | 0.00  (0.00-0.00) | 7.89  (5.08-11.59) | 3.45  (2.33-6.03) | 0.000* | 0.000* | 0.001* | 0.202 | 0.000* | 0.967 | 0.000* | 0.000* | 0.000* | 0.001* |
| miR744-5p | 633.30  (508.83-940.44) | 183.55  (143.62-429.38) | 2675.74  (1505.30-3920.32) | 532.61  (270.89-1016.20) | 702.27  (533.08-1054.46) | 0.002* | 0.000* | 0.624 | 0.595 | 0.000* | 0.002* | 0.000* | 0.000* | 0.000* | 0.098 |
| miR150-5p | 594.40  (292.07-948.20) | 2494.99  (1652.01-2883.73) | 7132.70  (6099.45-7997.60) | 16580.86  (12397.62-22349.10) | 8337.29  (3665.89-9781.25) | 0.000* | 0.000* | 0.000* | 0.000* | 0.000* | 0.000* | 0.006* | 0.000* | 0.539 | 0.000* |
| miR326-5p | 30.41  (20.69-82.12) | 167.20  (137.96-300.28) | 716.78  (404.45-980.57) | 1739.23  (1176.80-2654.43) | 626.40  (293.39-2239.69) | 0.000* | 0.000* | 0.000* | 0.000* | 0.000* | 0.000* | 0.000* | 0.001* | 0.775 | 0.074 |
| miR29a-5p | 3.69  (1.87-5.88) | 12.10  (0.95-14.01) | 5.01  (4.81-5.87) | 19.40  (7.37-22.94) | 16.41  (6.76-18.77) | 0.041* | 0.089 | 0.000* | 0.000* | 0.174 | 0.016* | 0.067 | 0.001* | 0.000* | 0.250 |
| miR21-5p | 1525.44  (783.65-1766.15) | 745.15  (214.22-879.01) | 63.68  (56.67-92.44) | 0.00  (0.00-0.00) | 75.90  (0.00-849.55) | 0.003* | 0.000* | 0.000* | 0.000* | 0.004* | 0.000* | 0.126 | 0.000* | 0.838 | 0.001* |
| miR15a-5p | 121.20  (71.68-137.30) | 232.57  (73.95-332.47) | 731.60  (463.80-1426.95) | 848.05  (493.40-1533.60) | 800.11  (397.80-1206.13) | 0.081 | 0.000* | 0.000* | 0.000* | 0.000* | 0.000* | 0.000* | 0.713 | 0.935 | 0.744 |
| miR221-5p | 0.91  (0.08-1.61) | 0.62  (0.00-0.88) | 13.52  (4.26-19.89) | 6.68  (3.99-8.28) | 0.66  (0.16-1.37) | 0.148 | 0.000* | 0.000* | 0.653 | 0.000* | 0.000* | 0.412 | 0.098 | 0.000* | 0.000* |
| miR486-5p | 313.38  (236.85-461.60) | 2444.78  (1924.90-2966.06) | 3707.07  (2424.25-4633.20) | 6051.83  (5316.52-6996.88) | 3282.33  (1749.17-5905.25) | 0.000* | 0.000* | 0.000* | 0.000* | 0.056 | 0.001* | 0.202 | 0.011* | 0.935 | 0.009* |

Legend: Group 2 – healthy volunteers (HV), Group 3 – patients with secondary immunodeficiency and EBV reactivation (SID EBV⁺), Group 4 – patients with secondary immunodeficiency without EBV reactivation (SID EBV⁻), Group 5 – patients with chronic lymphocytic leukemia and EBV reactivation (CLL EBV⁺), Group 6 – patients with chronic lymphocytic leukemia without EBV reactivation (CLL EBV⁻). Statistically significant results are marked with *.

**Tabel S7:** Statistical analyses of peripheral blood immunophenotype with special emphasis on the percentage of selected immune checkpoints and their ligands between SID and CLL and healthy volunteers including the status of EBV reactivation

| **Parameters** | **SID EBV+**  **(group 3)** | **SID EBV-**  **(group 4)** | **CLL EBV+**  **(group 5)** | **CLL EBV-**  **(group 6)** | **HV**  **(group 2)** | **p-Value** | | | | | | | | | |
| --- | --- | --- | --- | --- | --- | --- | --- | --- | --- | --- | --- | --- | --- | --- | --- |
|  | Median  (Q1-Q3) | Median  (Q1-Q3) | Median  (Q1-Q3) | Median  (Q1-Q3) | Median (Q1-Q3) | 3 vs 4 | 3 vs. 5 | 3 vs. 6 | 3 vs. 2 | 4 vs. 5 | 4 vs. 6 | 4 vs. 2 | 5 vs. 6 | 5 vs. 2 | 6 vs. 2 |
| CD45+ [%] | 93.91  (88.06-95.67) | 93.19  (90.47-96.41) | 90.42  (86.36-91.34) | 89.41  (87.29-92.22) | 93.63  (91.22-95.04) | 0.436 | 0.067 | 0.345 | 0.624 | 0.009* | 0.074 | 0.902 | 0.870 | 0.002* | 0.016* |
| CD3+ [%] | 27.15  (15.34-33.40) | 20.68  (17.07-21.82) | 18.72  (16.15-31.68) | 13.37  (10.82-21.11) | 74.14  (69.12-84.63) | 0.187 | 0.486 | 0.003* | 0.000* | 0.744 | 0.045* | 0.000* | 0.004* | 0.000* | 0.000* |
| T CD4+ lymphocytes[%] | 12.89  (7.61-19.16) | 10.41  (7.08-13.01) | 10.69  (6.77-17.00) | 8.40  (5.92-10.86) | 48.08  (46.51-53.67) | 0.217 | 0.744 | 0.037* | 0.000* | 0.367 | 0.267 | 0.000* | 0.045* | 0.000* | 0.000* |
| T CD8+ lymphocytes [%] | 12.07  (6.07-17.12) | 9.30  (6.24-13.94) | 10.18  (5.10-20.45) | 9.58  (6.11-12.71) | 39.06  (27.53-43.63) | 0.461 | 1.000 | 0.325 | 0.000* | 0.412 | 0.967 | 0.000* | 0.486 | 0.000* | 0.000* |
| B CD19+ lymphocytes [%] | 71.14  (56.39-77.75) | 59.08  (55.81-66.46) | 72.63  (68.59-75.15) | 63.48  (54.71-69.18) | 12.79  (8.22-13.88) | 0.202 | 0.653 | 0.202 | 0.000* | 0.015* | 0.713 | 0.000* | 0.011* | 0.000* | 0.000* |
| Ratio T CD4/ T CD8+ lymphocytes | 1.09  (0.48-2.19) | 1.13  (0.70-1.58) | 1.03  (0.42-2.48) | 1.03  (0.49-1.37) | 1.28  (1.16-1.95) | 0.967 | 1.000 | 0.436 | 0.267 | 0.744 | 0.305 | 0.116 | 0.412 | 0.412 | 0.004* |
| CD4+PD-1+ [%] | 35.73  (27.48-45.51) | 13.61  (11.46-15.78) | 32.61  (28.24-41.09) | 13.48  (9.15-14.75) | 3.54  (2.31-4.53) | 0.000* | 0.345 | 0.000* | 0.000* | 0.000* | 0.202 | 0.000* | 0.000* | 0.000* | 0.000* |
| CD8+PD-1+ [%] | 25.57  (20.60-30.54) | 10.27  (6.81-11.50) | 24.29  (22.02-26.96) | 6.30  (4.19-8.25) | 3.63  (2.45-4.31) | 0.000* | 0.367 | 0.000* | 0.000* | 0.000* | 0.013* | 0.000* | 0.000* | 0.000* | 0.000* |
| CD19+PD-1+[%] | 32.48  (20.89-36.47) | 10.68  (7.81-12.81) | 22.33  (16.86-28.25) | 8.15  (7.02-11.04) | 3.96  (2.65-4.70) | 0.000* | 0.004* | 0.000* | 0.000* | 0.000* | 0.217 | 0.000* | 0.000* | 0.000* | 0.000* |
| CD4+PD-L1+[%] | 16.64  (13.96-17.26) | 7.35  (5.33-8.82) | 13.15  (12.56-14.85) | 5.28  (4.52-7.56) | 0.73  (0.27-1.00) | 0.000* | 0.001* | 0.000* | 0.000* | 0.000* | 0.116 | 0.000* | 0.000* | 0.000* | 0.000* |
| CD8+PD-L1+[%] | 17.45  (14.20-19.52) | 5.06  (4.25-7.82) | 14.43  (13.29-17.77) | 3.86  (2.78-5.60) | 0.51  (0.37-0.70) | 0.000* | 0.081 | 0.000* | 0.000* | 0.000* | 0.011* | 0.000* | 0.000* | 0.000* | 0.000* |
| CD19+PD-L1+[%] | 17.60  (15.94-20.28) | 5.14  (4.17-8.29) | 16.39  (14.62-18.14) | 7.42  (5.10-9.99) | 0.67  (0.28-1.40) | 0.000* | 0.089 | 0.000* | 0.000* | 0.000* | 0.074 | 0.000* | 0.000* | 0.000* | 0.000* |
| CD4+CTLA-4+[%] | 22.81  (18.14-28.96) | 7.46  (6.26-9.94) | 26.86  (22.88-27.49) | 6.18  (5.25-8.91) | 3.10  (2.54-3.75) | 0.000* | 0.412 | 0.000* | 0.000* | 0.000* | 0.285 | 0.000* | 0.000* | 0.000* | 0.000* |
| CD8+CTLA-4+[%] | 25.00  (22.09-28.11) | 10.29  (9.30-11.60) | 23.22  (18.97-25.63) | 9.57  (7.75-13.17) | 3.20  (2.93-4.26) | 0.000* | 0.074 | 0.000* | 0.000* | 0.000* | 0.744 | 0.000* | 0.000* | 0.000* | 0.000* |
| CD19+CTLA-4+[%] | 8.73  (7.76-11.40) | 1.88  (1.35-2.85) | 8.01  (5.79-8.82) | 1.90  (1.13-3.24) | 2.13  (1.90-2.55) | 0.000* | 0.021* | 0.000* | 0.000* | 0.000* | 0.775 | 0.744 | 0.000* | 0.000* | 0.624 |
| CD4+CD86+[%] | 11.05  (8.59-12.11) | 5.28  (4.78-5.60) | 9.13  (7.81-11.08) | 4.66  (3.40-5.39) | 2.81  (2.55-3.63) | 0.000* | 0.089 | 0.000* | 0.000* | 0.000* | 0.045* | 0.000* | 0.000* | 0.000* | 0.001* |
| CD8+CD86+[%] | 8.43  (6.25-9.91) | 4.25  (3.36-4.64) | 7.50  (6.39-9.24) | 3.23  (2.49-3.99) | 1.97  (1.40-2.78) | 0.000* | 0.345 | 0.000* | 0.000* | 0.000* | 0.004* | 0.000* | 0.000* | 0.000* | 0.000* |
| CD19+CD86+[%] | 53.75  (51.21-56.29) | 33.97  (30.14-36.77) | 43.65  (42.21-51.91) | 28.48  (21.84-34.67) | 12.08  (9.35-16.11) | 0.000* | 0.000* | 0.000* | 0.000* | 0.000* | 0.021* | 0.000* | 0.000* | 0.000* | 0.000* |
| CD4+CD200R+[%] | 17.99  (13.49-21.74) | 5.25  (4.30-8.33) | 17.38  (13.73-18.94) | 5.86  (3.69-6.35) | 3.61  (2.36-6.24) | 0.000* | 0.624 | 0.000* | 0.000* | 0.000* | 0.539 | 0.011* | 0.000* | 0.000* | 0.106 |
| CD8+CD200R+[%] | 21.98  (17.18-24.99) | 6.71  (5.27-7.01) | 19.45  (14.33-22.80) | 6.12  (3.97-6.51) | 5.34  (2.31-5.62) | 0.000* | 0.089 | 0.000* | 0.000* | 0.000* | 0.250 | 0.007* | 0.000* | 0.000* | 0.148 |
| CD19+CD200R+[%] | 26.07  (18.29-29.16) | 9.58  (8.00-11.26) | 20.87  (16.21-25.62) | 7.40  (5.73-9.09) | 23.06  (18.23-25.27) | 0.000* | 0.061 | 0.000* | 0.217 | 0.000* | 0.041* | 0.000* | 0.000* | 0.567 | 0.000* |
| CD4+CD200+[%] | 37.24  (34.62-41.56) | 12.08  (7.55-14.37) | 36.32  (26.96-40.36) | 7.59  (6.01-10.14) | 3.03  (1.85-3.48) | 0.000* | 0.267 | 0.000* | 0.000* | 0.000* | 0.021* | 0.000* | 0.000* | 0.000* | 0.000* |
| CD8+200+[%] | 32.25  (27.80-33.34) | 9.75  (6.68-11.54) | 25.40  (21.20-26.78) | 8.57  (6.73-10.75) | 3.79  (2.55-4.68) | 0.000* | 0.000* | 0.000* | 0.000* | 0.000* | 0.713 | 0.000* | 0.000* | 0.000* | 0.000* |
| CD19+CD200+ [%] | 96.58  (92.41-98.06) | 70.65  (66.94-72.43) | 86.64  (82.40-90.67) | 64.43  (41.81-66.63) | 42.22  (31.93-54.43) | 0.000* | 0.000* | 0.000* | 0.000* | 0.000* | 0.005* | 0.000* | 0.000* | 0.000* | 0.013* |

Legend: Group 2 – healthy volunteers (HV), Group 3 – patients with secondary immunodeficiency and EBV reactivation (SID EBV⁺), Group 4 – patients with secondary immunodeficiency without EBV reactivation (SID EBV⁻), Group 5 – patients with chronic lymphocytic leukemia and EBV reactivation (CLL EBV⁺), Group 6 – patients with chronic lymphocytic leukemia without EBV reactivation (CLL EBV⁻). Statistically significant results are marked with *.

**Tabel S8:** Statistical analyses of serum concentrations of soluble forms of selected immune checkpoints and their ligands between SID and CLL and healthy volunteers including the status of EBV reactivation

| **Parameters** | **SID EBV+**  **(group 3)** | **SID EBV-**  **(group 4)** | **CLL EBV+**  **(group 5)** | **CLL EBV-**  **(group 6)** | **HV**  **(group 2)** | **p-Value** | | | | | | | | | |
| --- | --- | --- | --- | --- | --- | --- | --- | --- | --- | --- | --- | --- | --- | --- | --- |
|  | Median (Q1-Q3) | Median (Q1-Q3) | Median (Q1-Q3) | Median (Q1-Q3) | Median (Q1-Q3) | 3 vs 4 | 3 vs. 5 | 3 vs. 6 | 3 vs. 2 | 4 vs. 5 | 4 vs. 6 | 4 vs. 2 | 5 vs. 6 | 5 vs. 2 | 6 vs. 2 |
| sPD-1 [ng/ml] | 57.60  (50.81-60.36) | 36.86  (32.76-40.32) | 46.07  (39.59-53.76) | 31.98  (26.20-40.81) | 2.55  (1.55-4.10) | 0.000* | 0.001* | 0.000* | 0.000* | 0.000* | 0.233 | 0.000* | 0.000* | 0.000* | 0.000* |
| sPD-L1 [ng/ml] | 37.51  (32.95-39.89) | 23.71  (21.70-24.28) | 33.27  (30.16-34.93) | 20.69  (14.27-22.89) | 1.75  (0.83-2.07) | 0.000* | 0.011* | 0.000* | 0.000* | 0.000* | 0.007* | 0.000* | 0.000* | 0.000* | 0.000* |
| sCTLA-4 [ng/ml] | 28.73  (26.60-30.57) | 18.10  (16.46-19.27) | 25.76  (24.11-28.19) | 16.42  (12.95-20.18) | 3.12  (2.42-4.35) | 0.000* | 0.002* | 0.000* | 0.000* | 0.000* | 0.174 | 0.000* | 0.000* | 0.000* | 0.000* |
| sCD86 [ng/ml] | 24.85  (23.98-27.16) | 17.38  (15.53-17.93) | 22.49  (20.73-23.53) | 15.28  (11.34-17.87) | 1.75  (1.53-2.59) | 0.000* | 0.000* | 0.000* | 0.000* | 0.000* | 0.116 | 0.000* | 0.000* | 0.000* | 0.000* |
| sCD200R [ng/ml] | 45.12  (39.68-49.15) | 27.28  (25.71-30.12) | 38.44  (35.20-43.24) | 25.73  (17.29-29.10) | 4.57  (2.52-6.15) | 0.000* | 0.000* | 0.000* | 0.000* | 0.000* | 0.126 | 0.000* | 0.000* | 0.000* | 0.000* |
| sCD200 [ng/ml] | 58.09  (53.34-61.96) | 32.95  (31.69-36.10) | 50.52  (45.03-56.83) | 30.97  (20.72-33.74) | 2.17  (1.50-4.08) | 0.000* | 0.026* | 0.000* | 0.000* | 0.000* | 0.041* | 0.000* | 0.000* | 0.000* | 0.000* |

Legend: Group 2 – healthy volunteers (HV), Group 3 – patients with secondary immunodeficiency and EBV reactivation (SID EBV⁺), Group 4 – patients with secondary immunodeficiency without EBV reactivation (SID EBV⁻), Group 5 – patients with chronic lymphocytic leukemia and EBV reactivation (CLL EBV⁺), Group 6 – patients with chronic lymphocytic leukemia without EBV reactivation (CLL EBV⁻). Statistically significant results are marked with *.

**Table S9.** Spearman rank correlation for patients with SID EBV+

| Pair of variables | N | R | t(N-2) | p |
| --- | --- | --- | --- | --- |
| miR 29a-5p & VCA IgM | 15 | -0.803 | -4.850 | 0.000 |
| miR 21-5p & EBV copy number | 15 | -0.786 | -4.580 | 0.001 |
| miR 144-5p & VCA IgM | 15 | -0.782 | -4.524 | 0.001 |
| miR125b-5p & CD19+PD-1+ | 15 | -0.768 | -4.322 | 0.001 |
| miR 326-5p & VCA IgM | 15 | -0.711 | -3.643 | 0.003 |
| miR 744-5p & Upper respiratory tract infections | 15 | -0.710 | -3.630 | 0.003 |
| miR 134-5p & EBNA IgA | 15 | -0.657 | -3.143 | 0.008 |
| miR 30c-5p & CD8+CD86+ | 14 | -0.640 | -2.882 | 0.014 |
| miR 29a-5p & EA IgG | 15 | -0.634 | -2.960 | 0.011 |
| miR 16-5p & EBNA IgG | 15 | -0.632 | -2.941 | 0.011 |
| miR 144-5p & EBV copy number | 15 | -0.629 | -2.916 | 0.012 |
| miR 134-5p & CD8+PD-L1+ | 15 | -0.614 | -2.807 | 0.015 |
| miR125b-5p & EBNA IgG | 15 | -0.600 | -2.704 | 0.018 |
| miR 29a-5p & EBV copy number | 15 | -0.599 | -2.695 | 0.018 |
| miR 744-5p & miR 150-5p | 15 | -0.596 | -2.679 | 0.019 |
| miR 150-5p & miR 744-5p | 15 | -0.596 | -2.679 | 0.019 |
| miR 142-5p & EBV copy number | 15 | -0.557 | -2.419 | 0.031 |
| miR 150-5p & CD8+CTLA-4+ | 15 | -0.554 | -2.397 | 0.032 |
| miR 33a-5p & EBV copy number | 15 | -0.550 | -2.374 | 0.034 |
| miR 150-5p & miR 221-5p | 15 | -0.545 | -2.342 | 0.036 |
| miR 221-5p & miR 150-5p | 15 | -0.545 | -2.342 | 0.036 |
| miR 142-5p & Serum concentration sCTLA-4 | 15 | -0.536 | -2.287 | 0.040 |
| miR 28-5p & Inflammation of the skin and soft tissue | 15 | -0.526 | -2.229 | 0.044 |
| miR 142-5p & miR 744-5p | 15 | 0.514 | 2.162 | 0.050 |
| miR 33a-5p & miR 486-5p | 15 | 0.521 | 2.203 | 0.046 |
| miR 486-5p & Serum concentration sCD86 | 15 | 0.521 | 2.203 | 0.046 |
| miR 21-5p & CD19+ | 15 | 0.525 | 2.224 | 0.044 |
| miR 28-5p & miR 134-5p | 15 | 0.529 | 2.245 | 0.043 |
| miR 155-5p & miR 15a-5p | 15 | 0.536 | 2.287 | 0.040 |
| miR 181a-5p & miR 486-5p | 15 | 0.536 | 2.287 | 0.040 |
| miR 33a-5p & Serum concentration sPD-L1 | 15 | 0.536 | 2.287 | 0.040 |
| miR125b-5p & miR 144-5p | 15 | 0.537 | 2.295 | 0.039 |
| miR 33a-5p & EA IgM | 15 | 0.538 | 2.301 | 0.039 |
| miR 16-5p & CD19+CD200+ | 15 | 0.539 | 2.309 | 0.038 |
| miR 181a-5p & Serum concentration sPD-L1 | 15 | 0.539 | 2.309 | 0.038 |
| miR 486-5p & EA IgA | 15 | 0.543 | 2.331 | 0.037 |
| miR 155-5p & miR 33a-5p | 15 | 0.546 | 2.352 | 0.035 |
| miR 155-5p & CD4+PD-L1+ | 15 | 0.546 | 2.352 | 0.035 |
| miR 144-5p & Serum concentration sPD-L1 | 15 | 0.557 | 2.417 | 0.031 |
| miR 744-5p & VCA IgG | 15 | 0.557 | 2.419 | 0.031 |
| miR 28-5p & Serum concentration sCD86 | 15 | 0.561 | 2.442 | 0.030 |
| miR 181a-5p & EA IgM | 15 | 0.561 | 2.445 | 0.029 |
| miR 134-5p & miR 326-5p | 15 | 0.579 | 2.558 | 0.024 |
| miR125b-5p & miR 21-5p | 15 | 0.579 | 2.558 | 0.024 |
| miR 33a-5p & miR 142-5p | 15 | 0.579 | 2.558 | 0.024 |
| miR 21-5p & EA IgA | 15 | 0.582 | 2.581 | 0.023 |
| miR 28-5p & miR 15a-5p | 15 | 0.593 | 2.654 | 0.020 |
| miR 134-5p & miR 21-5p | 15 | 0.596 | 2.679 | 0.019 |
| miR 21-5p & miR 134-5p | 15 | 0.596 | 2.679 | 0.019 |
| miR 30c-5p & EBNA IgM | 14 | 0.600 | 2.598 | 0.023 |
| miR 144-5p & miR 326-5p | 15 | 0.602 | 2.717 | 0.018 |
| miR 181a-5p & miR 28-5p | 15 | 0.604 | 2.729 | 0.017 |
| miR 29a-5p & miR 21-5p | 15 | 0.613 | 2.798 | 0.015 |
| miR 28-5p & miR 33a-5p | 15 | 0.632 | 2.941 | 0.011 |
| miR 29a-5p & miR 326-5p | 15 | 0.638 | 2.988 | 0.010 |
| miR 142-5p & VCA IgA | 15 | 0.639 | 2.997 | 0.010 |
| miR 134-5p & CD8+PD-1+ | 15 | 0.650 | 3.084 | 0.009 |
| miR 486-5p & CD4+PD-1+ | 15 | 0.650 | 3.084 | 0.009 |
| miR 30c-5p & miR 33a-5p | 14 | 0.657 | 3.020 | 0.011 |
| miR 21-5p & miR 326-5p | 15 | 0.657 | 3.143 | 0.008 |
| miR 155-5p & miR 181a-5p | 15 | 0.679 | 3.331 | 0.005 |
| miR 28-5p & miR 486-5p | 15 | 0.686 | 3.397 | 0.005 |
| miR 181a-5p & miR 21-5p | 15 | 0.686 | 3.397 | 0.005 |
| miR 28-5p & CD8+CD200R+ | 15 | 0.689 | 3.430 | 0.004 |
| miR 144-5p & miR 21-5p | 15 | 0.703 | 3.561 | 0.003 |
| miR 16-5p & VCA IgA | 15 | 0.704 | 3.570 | 0.003 |
| miR 155-5p & CD4+PD-1+ | 15 | 0.711 | 3.643 | 0.003 |
| miR 30c-5p & CD8+CD200R+ | 14 | 0.714 | 3.536 | 0.004 |
| miR 155-5p & miR 486-5p | 15 | 0.721 | 3.756 | 0.002 |
| miR 33a-5p & miR 21-5p | 15 | 0.721 | 3.756 | 0.002 |
| miR 30c-5p & miR 28-5p | 14 | 0.758 | 4.029 | 0.002 |
| miR 16-5p & EA IgM | 15 | 0.760 | 4.211 | 0.001 |
| miR 144-5p & miR 29a-5p | 15 | 0.781 | 4.508 | 0.001 |
| miR 15a-5p & miR 486-5p | 15 | 0.818 | 5.125 | 0.000 |
| miR 181a-5p & CD8+CD200R+ | 15 | 0.825 | 5.264 | 0.000 |
| miR 33a-5p & CD8+CD200R+ | 15 | 0.829 | 5.336 | 0.000 |
| miR 181a-5p & miR 33a-5p | 15 | 0.900 | 7.445 | 0.000 |

**Table S10.** Spearman rank correlation for patients with SID EBV-

| Pair of variables | N | R | t(N-2) | p |
| --- | --- | --- | --- | --- |
| miR125b-5p & EBNA IgM | 15 | -0.746 | -4.044 | 0.001 |
| miR125b-5p & CD19+CD86+ | 15 | -0.739 | -3.958 | 0.002 |
| miR 744-5p & EBNA IgG | 15 | -0.693 | -3.464 | 0.004 |
| miR 150-5p & CD8+PD-L1+ | 15 | -0.691 | -3.442 | 0.004 |
| miR 29a-5p & CD8+ | 15 | -0.690 | -3.436 | 0.004 |
| miR 30c-5p & CD45+ | 15 | -0.675 | -3.299 | 0.006 |
| miR 29a-5p & Serum concentration sCD200 | 15 | -0.672 | -3.272 | 0.006 |
| miR 155-5p & VCA IgM | 15 | -0.668 | -3.235 | 0.007 |
| miR 134-5p & CD19+CD86+ | 15 | -0.657 | -3.143 | 0.008 |
| miR 21-5p & CD8+ | 15 | -0.650 | -3.084 | 0.009 |
| miR 134-5p & CD19+CTLA-4+ | 15 | -0.640 | -3.002 | 0.010 |
| miR 326-5p & EBNA IgM | 15 | -0.632 | -2.941 | 0.011 |
| miR 155-5p & EBNA IgA | 15 | -0.629 | -2.918 | 0.012 |
| miR 150-5p & Genitourinary tract infections | 15 | -0.628 | -2.909 | 0.012 |
| miR 744-5p & CD19+PD-1+ | 15 | -0.625 | -2.887 | 0.013 |
| miR 744-5p & CD19+PD-L1+ | 15 | -0.624 | -2.877 | 0.013 |
| miR 16-5p & CD19+CD86+ | 15 | -0.611 | -2.781 | 0.016 |
| miR 142-5p & CD19+CTLA-4+ | 15 | -0.590 | -2.633 | 0.021 |
| miR 144-5p & Upper respiratory tract infections | 15 | -0.589 | -2.629 | 0.021 |
| miR 28-5p & CD19+CTLA-4+ | 15 | -0.568 | -2.491 | 0.027 |
| miR 744-5p & CD45+ | 15 | -0.564 | -2.464 | 0.028 |
| miR 142-5p & Genitourinary tract infections | 15 | -0.564 | -2.461 | 0.029 |
| miR 30c-5p & VCA IgA | 15 | -0.550 | -2.374 | 0.034 |
| miR 134-5p & CD19+PD-1+ | 15 | -0.550 | -2.374 | 0.034 |
| miR 28-5p & CD19+PD-1+ | 15 | -0.543 | -2.331 | 0.037 |
| miR 15a-5p & CD19+CD86+ | 15 | -0.543 | -2.331 | 0.037 |
| miR 134-5p & VCA IgA | 15 | -0.539 | -2.309 | 0.038 |
| miR 30c-5p & CD8+ | 15 | -0.532 | -2.266 | 0.041 |
| miR 221-5p & CD8+ | 15 | -0.526 | -2.231 | 0.044 |
| miR 221-5p & Serum concentration sCD200 | 15 | -0.524 | -2.220 | 0.045 |
| miR 33a-5p & CD8+ | 15 | -0.521 | -2.203 | 0.046 |
| miR 29a-5p & CD19+CD200+ | 15 | -0.517 | -2.175 | 0.049 |
| miR 29a-5p & miR 221-5p | 15 | 0.516 | 2.171 | 0.049 |
| miR 155-5p & miR 144-5p | 15 | 0.518 | 2.183 | 0.048 |
| miR 30c-5p & miR 221-5p | 15 | 0.519 | 2.189 | 0.047 |
| miR 30c-5p & miR 142-5p | 15 | 0.521 | 2.203 | 0.046 |
| miR 134-5p & miR 15a-5p | 15 | 0.521 | 2.203 | 0.046 |
| miR 744-5p & miR 134-5p | 15 | 0.525 | 2.224 | 0.044 |
| miR 486-5p & CD19+CD200+ | 15 | 0.525 | 2.224 | 0.044 |
| miR125b-5p & miR 142-5p | 15 | 0.532 | 2.266 | 0.041 |
| miR 21-5p & Stosunek CD4/CD8+ | 15 | 0.532 | 2.266 | 0.041 |
| miR 150-5p & Serum concentration sCD86 | 15 | 0.532 | 2.266 | 0.041 |
| miR 15a-5p & CD4+CD200R+ | 15 | 0.542 | 2.323 | 0.037 |
| miR 134-5p & miR125b-5p | 15 | 0.543 | 2.331 | 0.037 |
| miR 28-5p & Stosunek CD4/CD8+ | 15 | 0.546 | 2.352 | 0.035 |
| miR 744-5p & Serum concentration sCD200 | 15 | 0.546 | 2.352 | 0.035 |
| miR 134-5p & miR 142-5p | 15 | 0.554 | 2.397 | 0.032 |
| miR 16-5p & Stosunek CD4/CD8+ | 15 | 0.554 | 2.397 | 0.032 |
| miR 144-5p & Gastrointestinal infections | 15 | 0.555 | 2.403 | 0.032 |
| miR125b-5p & miR 33a-5p | 15 | 0.561 | 2.442 | 0.030 |
| miR 142-5p & miR 150-5p | 15 | 0.564 | 2.464 | 0.028 |
| miR 30c-5p & CD4+CD200R+ | 15 | 0.567 | 2.479 | 0.028 |
| miR 16-5p & miR 30c-5p | 15 | 0.568 | 2.487 | 0.027 |
| miR 21-5p & miR 181a-5p | 15 | 0.568 | 2.487 | 0.027 |
| miR 486-5p & CD4+PD-L1+ | 15 | 0.571 | 2.511 | 0.026 |
| miR 150-5p & EA IgG | 15 | 0.575 | 2.534 | 0.025 |
| miR 142-5p & miR 221-5p | 15 | 0.577 | 2.545 | 0.024 |
| miR 30c-5p & CD8+CD86+ | 15 | 0.577 | 2.549 | 0.024 |
| miR 221-5p & Stosunek CD4/CD8+ | 15 | 0.584 | 2.593 | 0.022 |
| miR 486-5p & VCA IgM | 15 | 0.586 | 2.606 | 0.022 |
| miR 15a-5p & miR 221-5p | 15 | 0.587 | 2.617 | 0.021 |
| miR 33a-5p & miR 28-5p | 15 | 0.589 | 2.630 | 0.021 |
| miR 28-5p & miR 29a-5p | 15 | 0.592 | 2.646 | 0.020 |
| miR 181a-5p & Stosunek CD4/CD8+ | 15 | 0.596 | 2.679 | 0.019 |
| miR 150-5p & Gastrointestinal infections | 15 | 0.601 | 2.711 | 0.018 |
| miR 30c-5p & miR 33a-5p | 15 | 0.604 | 2.729 | 0.017 |
| miR 28-5p & CD4+CD200R+ | 15 | 0.604 | 2.733 | 0.017 |
| miR 30c-5p & Stosunek CD4/CD8+ | 15 | 0.611 | 2.781 | 0.016 |
| miR 744-5p & CD4+CTLA-4+ | 15 | 0.611 | 2.781 | 0.016 |
| miR 29a-5p & miR 21-5p | 15 | 0.611 | 2.785 | 0.015 |
| miR 15a-5p & miR125b-5p | 15 | 0.614 | 2.807 | 0.015 |
| miR 33a-5p & miR 221-5p | 15 | 0.618 | 2.835 | 0.014 |
| miR 155-5p & CD4+CD86+ | 15 | 0.629 | 2.914 | 0.012 |
| miR 744-5p & EA IgM | 15 | 0.634 | 2.960 | 0.011 |
| miR 16-5p & CD4+CD200R+ | 15 | 0.636 | 2.974 | 0.011 |
| miR 28-5p & miR 181a-5p | 15 | 0.639 | 2.997 | 0.010 |
| miR 142-5p & miR 33a-5p | 15 | 0.639 | 2.997 | 0.010 |
| miR 16-5p & miR 142-5p | 15 | 0.650 | 3.084 | 0.009 |
| miR 30c-5p & miR 134-5p | 15 | 0.657 | 3.143 | 0.008 |
| miR 28-5p & miR 30c-5p | 15 | 0.657 | 3.143 | 0.008 |
| miR 181a-5p & miR 15a-5p | 15 | 0.661 | 3.174 | 0.007 |
| miR 15a-5p & miR 21-5p | 15 | 0.661 | 3.174 | 0.007 |
| miR 16-5p & miR 181a-5p | 15 | 0.668 | 3.235 | 0.007 |
| miR 144-5p & Serum concentration sCTLA-4 | 15 | 0.668 | 3.235 | 0.007 |
| miR 181a-5p & miR 221-5p | 15 | 0.668 | 3.241 | 0.006 |
| miR 326-5p & miR 144-5p | 15 | 0.671 | 3.267 | 0.006 |
| miR 144-5p & CD45+ | 15 | 0.682 | 3.364 | 0.005 |
| miR 30c-5p & miR 29a-5p | 15 | 0.704 | 3.576 | 0.003 |
| miR 15a-5p & miR 16-5p | 15 | 0.707 | 3.606 | 0.003 |
| miR 16-5p & miR 33a-5p | 15 | 0.714 | 3.680 | 0.003 |
| miR 33a-5p & miR 16-5p | 15 | 0.714 | 3.680 | 0.003 |
| miR 155-5p & CD8+200+ | 15 | 0.718 | 3.718 | 0.003 |
| miR 29a-5p & Stosunek CD4/CD8+ | 15 | 0.735 | 3.903 | 0.002 |
| miR 134-5p & miR 33a-5p | 15 | 0.743 | 4.001 | 0.002 |
| miR 221-5p & CD4+CD200R+ | 15 | 0.748 | 4.069 | 0.001 |
| miR 16-5p & miR 221-5p | 15 | 0.753 | 4.128 | 0.001 |
| miR 28-5p & miR 221-5p | 15 | 0.753 | 4.128 | 0.001 |
| miR 16-5p & miR 134-5p | 15 | 0.754 | 4.133 | 0.001 |
| miR 29a-5p & CD8+CD86+ | 15 | 0.789 | 4.629 | 0.000 |
| miR 28-5p & miR 134-5p | 15 | 0.800 | 4.807 | 0.000 |
| miR 28-5p & miR 16-5p | 15 | 0.814 | 5.058 | 0.000 |
| miR 326-5p & CD4+CD86+ | 15 | 0.861 | 6.096 | 0.000 |

Table S11. Spearman rank correlation for patients with CLL EBV+

| Pair of variables | N | R | t(N-2) | p |
| --- | --- | --- | --- | --- |
| miR 15a-5p & CD19+CD200R+ | 15 | -0.764 | -4.273 | 0.001 |
| miR 33a-5p & miR 150-5p | 15 | -0.704 | -3.570 | 0.003 |
| miR 21-5p & EBNA IgG | 15 | -0.700 | -3.534 | 0.004 |
| miR 155-5p & VCA IgA | 15 | -0.686 | -3.397 | 0.005 |
| miR 16-5p & miR 150-5p | 15 | -0.682 | -3.364 | 0.005 |
| miR 21-5p & EBNA IgM | 15 | -0.668 | -3.235 | 0.007 |
| miR 29a-5p & miR 486-5p | 15 | -0.660 | -3.163 | 0.007 |
| miR 30c-5p & Lower respiratory tract infections | 15 | -0.656 | -3.132 | 0.008 |
| miR 21-5p & CD19+CTLA-4+ | 15 | -0.627 | -2.905 | 0.012 |
| miR 221-5p & CD4+CD200+ | 15 | -0.625 | -2.887 | 0.013 |
| miR 16-5p & CD19+PD-1+ | 15 | -0.611 | -2.781 | 0.016 |
| miR 134-5p & CD19+CD86+ | 15 | -0.604 | -2.729 | 0.017 |
| miR 155-5p & miR 21-5p | 15 | -0.600 | -2.704 | 0.018 |
| miR 150-5p & miR 30c-5p | 15 | -0.596 | -2.679 | 0.019 |
| miR 28-5p & VCA IgA | 15 | -0.596 | -2.679 | 0.019 |
| miR 150-5p & Genitourinary tract infections | 15 | -0.590 | -2.635 | 0.021 |
| miR 150-5p & Stosunek CD4/CD8+ | 15 | -0.586 | -2.606 | 0.022 |
| miR 16-5p & Serum concentration sPD-1 | 15 | -0.586 | -2.606 | 0.022 |
| miR 150-5p & miR 21-5p | 15 | -0.582 | -2.581 | 0.023 |
| miR 134-5p & CD45+ | 15 | -0.561 | -2.445 | 0.029 |
| miR 30c-5p & CD8+ | 15 | -0.550 | -2.374 | 0.034 |
| miR 181a-5p & EBNA IgG | 15 | -0.546 | -2.352 | 0.035 |
| miR 33a-5p & Lower respiratory tract infections | 15 | -0.540 | -2.314 | 0.038 |
| miR 15a-5p & Lower respiratory tract infections | 15 | -0.540 | -2.314 | 0.038 |
| miR 221-5p & Lower respiratory tract infections | 15 | -0.540 | -2.314 | 0.038 |
| miR 134-5p & Serum concentration sPD-1 | 15 | -0.539 | -2.309 | 0.038 |
| miR 33a-5p & CD19+PD-1+ | 15 | -0.536 | -2.287 | 0.040 |
| miR 134-5p & EBV copy number | 15 | -0.532 | -2.266 | 0.041 |
| miR 155-5p & CD19+CD200R+ | 15 | -0.532 | -2.266 | 0.041 |
| miR 29a-5p & VCA IgA | 15 | -0.524 | -2.216 | 0.045 |
| miR125b-5p & miR 15a-5p | 15 | 0.514 | 2.162 | 0.050 |
| miR 33a-5p & CD4+CD200R+ | 15 | 0.514 | 2.162 | 0.050 |
| miR125b-5p & miR 142-5p | 15 | 0.518 | 2.183 | 0.048 |
| miR 28-5p & CD19+CTLA-4+ | 15 | 0.529 | 2.248 | 0.043 |
| miR 16-5p & miR 30c-5p | 15 | 0.532 | 2.266 | 0.041 |
| miR 142-5p & CD19+CTLA-4+ | 15 | 0.536 | 2.290 | 0.039 |
| miR 142-5p & CD4+CD200R+ | 15 | 0.539 | 2.309 | 0.038 |
| miR 181a-5p & CD4+ | 15 | 0.543 | 2.334 | 0.036 |
| miR 155-5p & miR 15a-5p | 15 | 0.546 | 2.352 | 0.035 |
| miR 181a-5p & CD4+CD86+ | 15 | 0.546 | 2.352 | 0.035 |
| miR 30c-5p & Stosunek CD4/CD8+ | 15 | 0.550 | 2.374 | 0.034 |
| miR 150-5p & CD19+PD-1+ | 15 | 0.564 | 2.464 | 0.028 |
| miR 28-5p & miR125b-5p | 15 | 0.568 | 2.487 | 0.027 |
| miR 326-5p & miR 33a-5p | 15 | 0.568 | 2.487 | 0.027 |
| miR 15a-5p & Serum concentration sCTLA-4 | 15 | 0.568 | 2.487 | 0.027 |
| miR 134-5p & EA IgA | 15 | 0.575 | 2.534 | 0.025 |
| miR 16-5p & miR 33a-5p | 15 | 0.579 | 2.558 | 0.024 |
| miR 30c-5p & EBV copy number | 15 | 0.579 | 2.558 | 0.024 |
| miR 142-5p & CD8+PD-1+ | 15 | 0.586 | 2.606 | 0.022 |
| miR 181a-5p & CD8+PD-L1+ | 15 | 0.589 | 2.630 | 0.021 |
| miR 30c-5p & Genitourinary tract infections | 15 | 0.590 | 2.635 | 0.021 |
| miR 181a-5p & EBV copy number | 15 | 0.593 | 2.654 | 0.020 |
| miR 181a-5p & CD8+CTLA-4+ | 15 | 0.604 | 2.729 | 0.017 |
| miR125b-5p & miR 744-5p | 15 | 0.607 | 2.755 | 0.016 |
| miR 221-5p & CD19+ | 15 | 0.611 | 2.781 | 0.016 |
| miR 326-5p & EA IgG | 15 | 0.614 | 2.807 | 0.015 |
| miR 30c-5p & miR 181a-5p | 15 | 0.636 | 2.969 | 0.011 |
| miR 326-5p & miR 142-5p | 15 | 0.636 | 2.969 | 0.011 |
| miR 326-5p & CD4+CD200R+ | 15 | 0.643 | 3.026 | 0.010 |
| miR 155-5p & miR 326-5p | 15 | 0.646 | 3.055 | 0.009 |
| miR 28-5p & miR 326-5p | 15 | 0.646 | 3.055 | 0.009 |
| miR 155-5p & EA IgG | 15 | 0.657 | 3.143 | 0.008 |
| miR 30c-5p & miR 33a-5p | 15 | 0.664 | 3.204 | 0.007 |
| miR 150-5p & CD8+ | 15 | 0.671 | 3.267 | 0.006 |
| miR 142-5p & CD8+CD200R+ | 15 | 0.671 | 3.267 | 0.006 |
| miR125b-5p & Upper respiratory tract infections | 15 | 0.679 | 3.335 | 0.005 |
| miR125b-5p & Serum concentration s CR200R | 15 | 0.682 | 3.364 | 0.005 |
| miR 326-5p & CD8+PD-1+ | 15 | 0.693 | 3.464 | 0.004 |
| miR 221-5p & CD4+CD86+ | 15 | 0.696 | 3.499 | 0.004 |
| miR125b-5p & CD8+PD-1+ | 15 | 0.700 | 3.534 | 0.004 |
| miR 155-5p & Serum concentration s CR200R | 15 | 0.700 | 3.534 | 0.004 |
| miR 28-5p & Serum concentration s CR200R | 15 | 0.704 | 3.570 | 0.003 |
| miR 155-5p & miR125b-5p | 15 | 0.721 | 3.756 | 0.002 |
| miR 221-5p & Serum concentration sCTLA-4 | 15 | 0.732 | 3.875 | 0.002 |
| miR 326-5p & Serum concentration s CR200R | 15 | 0.736 | 3.917 | 0.002 |
| miR 142-5p & Serum concentration s CR200R | 15 | 0.786 | 4.580 | 0.001 |
| miR 28-5p & CD8+PD-1+ | 15 | 0.825 | 5.264 | 0.000 |
| miR 155-5p & miR 28-5p | 15 | 0.854 | 5.907 | 0.000 |
| miR 155-5p & CD8+PD-1+ | 15 | 0.857 | 6.000 | 0.000 |
| miR 142-5p & Serum concentration sPD-L1 | 15 | 0.871 | 6.405 | 0.000 |

**Table S12.** Spearman rank correlation for patients with CLL EBV-

| Pair of variables | N | R | t(N-2) | p |
| --- | --- | --- | --- | --- |
| miR 15a-5p & CD4+CTLA-4+ | 15 | -0.757 | -4.179 | 0.001 |
| miR 142-5p & CD8+CTLA-4+ | 15 | -0.671 | -3.267 | 0.006 |
| miR 15a-5p & CD45+ | 15 | -0.646 | -3.055 | 0.009 |
| miR 142-5p & CD4+CTLA-4+ | 15 | -0.643 | -3.026 | 0.010 |
| miR 30c-5p & CD45+ | 15 | -0.632 | -2.941 | 0.011 |
| miR 15a-5p & EBNA IgG | 15 | -0.621 | -2.860 | 0.013 |
| miR 28-5p & CD45+ | 15 | -0.618 | -2.833 | 0.014 |
| miR 15a-5p & CD8+CTLA-4+ | 15 | -0.614 | -2.807 | 0.015 |
| miR 15a-5p & CD8+ | 15 | -0.607 | -2.755 | 0.016 |
| miR 28-5p & CD4+CD200R+ | 15 | -0.607 | -2.755 | 0.016 |
| miR 144-5p & CD19+PD-1+ | 15 | -0.607 | -2.755 | 0.016 |
| miR 29a-5p & CD45+ | 15 | -0.596 | -2.679 | 0.019 |
| miR 28-5p & CD8+ | 15 | -0.582 | -2.581 | 0.023 |
| miR 142-5p & CD45+ | 15 | -0.582 | -2.581 | 0.023 |
| miR 142-5p & miR 150-5p | 15 | -0.575 | -2.534 | 0.025 |
| miR 142-5p & Serum concentration sCTLA-4 | 15 | -0.575 | -2.534 | 0.025 |
| miR 15a-5p & CD8+200+ | 15 | -0.564 | -2.464 | 0.028 |
| miR 30c-5p & CD4+CTLA-4+ | 15 | -0.561 | -2.442 | 0.030 |
| miR 30c-5p & EBNA IgG | 15 | -0.554 | -2.397 | 0.032 |
| miR 30c-5p & CD8+CTLA-4+ | 15 | -0.550 | -2.374 | 0.034 |
| miR 28-5p & CD8+200+ | 15 | -0.546 | -2.352 | 0.035 |
| miR 326-5p & CD8+PD-L1+ | 15 | -0.543 | -2.331 | 0.037 |
| miR 150-5p & CD8+ | 15 | -0.536 | -2.287 | 0.040 |
| miR 29a-5p & CD19+CD200+ | 15 | -0.536 | -2.287 | 0.040 |
| miR 142-5p & CD4+PD-1+ | 15 | -0.532 | -2.266 | 0.041 |
| miR 221-5p & CD8+ | 15 | -0.521 | -2.203 | 0.046 |
| miR 142-5p & miR 15a-5p | 15 | 0.514 | 2.162 | 0.050 |
| miR 28-5p & EBNA IgA | 15 | 0.514 | 2.162 | 0.050 |
| miR 33a-5p & Serum concentration sPD-1 | 15 | 0.514 | 2.162 | 0.050 |
| miR 181a-5p & miR 744-5p | 15 | 0.518 | 2.183 | 0.048 |
| miR 150-5p & miR 33a-5p | 15 | 0.518 | 2.183 | 0.048 |
| miR 30c-5p & miR 28-5p | 15 | 0.521 | 2.203 | 0.046 |
| miR 744-5p & CD19+CTLA-4+ | 15 | 0.521 | 2.203 | 0.046 |
| miR 142-5p & miR 221-5p | 15 | 0.525 | 2.224 | 0.044 |
| miR 33a-5p & CD4+PD-L1+ | 15 | 0.525 | 2.224 | 0.044 |
| miR 326-5p & miR 29a-5p | 15 | 0.532 | 2.266 | 0.041 |
| miR 744-5p & Serum concentration s CR200R | 15 | 0.532 | 2.266 | 0.041 |
| miR 744-5p & Serum concentration sCD200 | 15 | 0.536 | 2.287 | 0.040 |
| miR 28-5p & miR 326-5p | 15 | 0.543 | 2.331 | 0.037 |
| miR 221-5p & miR 744-5p | 15 | 0.543 | 2.331 | 0.037 |
| miR 181a-5p & VCA IgG | 15 | 0.543 | 2.331 | 0.037 |
| miR 33a-5p & CD8+CD86+ | 15 | 0.543 | 2.331 | 0.037 |
| miR 33a-5p & Serum concentration sPD-L1 | 15 | 0.546 | 2.352 | 0.035 |
| miR 30c-5p & miR125b-5p | 15 | 0.550 | 2.374 | 0.034 |
| miR 28-5p & miR 29a-5p | 15 | 0.550 | 2.374 | 0.034 |
| miR 744-5p & Serum concentration sPD-1 | 15 | 0.554 | 2.397 | 0.032 |
| miR 30c-5p & EBNA IgA | 15 | 0.557 | 2.419 | 0.031 |
| miR 16-5p & miR 134-5p | 15 | 0.561 | 2.442 | 0.030 |
| miR 744-5p & CD8+CD86+ | 15 | 0.564 | 2.464 | 0.028 |
| miR 134-5p & miR 326-5p | 15 | 0.568 | 2.487 | 0.027 |
| miR 150-5p & CD19+CD200R+ | 15 | 0.568 | 2.487 | 0.027 |
| miR 134-5p & miR 744-5p | 15 | 0.575 | 2.534 | 0.025 |
| miR 221-5p & miR 15a-5p | 15 | 0.579 | 2.558 | 0.024 |
| miR 744-5p & CD19+PD-L1+ | 15 | 0.589 | 2.630 | 0.021 |
| miR 150-5p & Serum concentration sCTLA-4 | 15 | 0.593 | 2.654 | 0.020 |
| miR 326-5p & EA IgM | 15 | 0.599 | 2.695 | 0.018 |
| miR 28-5p & miR 486-5p | 15 | 0.600 | 2.704 | 0.018 |
| miR125b-5p & miR 181a-5p | 15 | 0.611 | 2.781 | 0.016 |
| miR125b-5p & miR 15a-5p | 15 | 0.611 | 2.781 | 0.016 |
| miR 33a-5p & CD19+CD200+ | 15 | 0.611 | 2.781 | 0.016 |
| miR 33a-5p & Serum concentration sCD86 | 15 | 0.614 | 2.807 | 0.015 |
| miR 28-5p & miR 134-5p | 15 | 0.629 | 2.914 | 0.012 |
| miR 33a-5p & CD4+CD86+ | 15 | 0.629 | 2.914 | 0.012 |
| miR 33a-5p & Serum concentration sCTLA-4 | 15 | 0.636 | 2.969 | 0.011 |
| miR 155-5p & EA IgM | 15 | 0.660 | 3.163 | 0.007 |
| miR 30c-5p & miR 142-5p | 15 | 0.668 | 3.235 | 0.007 |
| miR 150-5p & Stosunek CD4/CD8+ | 15 | 0.668 | 3.235 | 0.007 |
| miR 744-5p & Serum concentration sCD86 | 15 | 0.682 | 3.364 | 0.005 |
| miR 30c-5p & miR 15a-5p | 15 | 0.714 | 3.680 | 0.003 |
| miR 221-5p & miR 28-5p | 15 | 0.721 | 3.756 | 0.002 |
| miR125b-5p & miR 486-5p | 15 | 0.729 | 3.835 | 0.002 |
| miR 28-5p & miR 16-5p | 15 | 0.782 | 4.526 | 0.001 |
| miR 221-5p & miR 16-5p | 15 | 0.782 | 4.526 | 0.001 |
| miR 33a-5p & CD19+CD200R+ | 15 | 0.829 | 5.336 | 0.000 |
| miR 181a-5p & miR 486-5p | 15 | 0.836 | 5.487 | 0.000 |

**Table S13.** ROC curve analysis for CLL and HV patients

| Paramteres | Area | Std. Error | 95% confidence interval | P value |
| --- | --- | --- | --- | --- |
| 16-5p | 0.65 | 0.06372 | 0,5251 to 0,7749 | 0.0738 |
| 155-5p | 0.7489 | 0.05974 | 0,6318 to 0,8660 | 0.003 |
| 30c-5p | 0.6407 | 0.06466 | 0,5139 to 0,7674 | 0.0941 |
| 28-5p | 0.6544 | 0.06569 | 0,5257 to 0,7832 | 0.0656 |
| 181a-5p | 0.6378 | 0.0653 | 0,5098 to 0,7658 | 0.1005 |
| 134a-5p | 0.7367 | 0.05459 | 0,6297 to 0,8437 | 0.0048 |
| 125b-5p | 0.6356 | 0.06226 | 0,5135 to 0,7576 | 0.1061 |
| 33a-5p | 0.6056 | 0.06907 | 0,4702 to 0,7409 | 0.2083 |
| 142-5p | 0.6367 | 0.06399 | 0,5113 to 0,7621 | 0.1033 |
| 144-5p | 0.52 | 0.06485 | 0,3929 to 0,6471 | 0.8116 |
| 744-5p | 0.5644 | 0.06809 | 0,4310 to 0,6979 | 0.4424 |
| 150-5p | 0.61 | 0.07342 | 0,4661 to 0,7539 | 0.1898 |
| 326-5p | 0.6589 | 0.06716 | 0,5273 to 0,7905 | 0.0582 |
| 29a-5p | 0.7178 | 0.06255 | 0,5952 to 0,8404 | 0.0094 |
| 21-5p | 0.5483 | 0.0804 | 0,3907 to 0,7059 | 0.5645 |
| 15a-5p | 0.7044 | 0.06524 | 0,5766 to 0,8323 | 0.0148 |
| 221-5p | 0.7006 | 0.0627 | 0,5777 to 0,8234 | 0.0168 |
| 486-5p | 0.5844 | 0.07336 | 0,4407 to 0,7282 | 0.3141 |
| CD4+PD-1+ [%] | 1 | 0 | 1,000 to 1,000 | <0,0001 |
| CD8 | 0.9617 | 0.01978 | 0,9229 to 1,000 | <0,0001 |
| CD19 | 0.9989 | 0.001871 | 0,9952 to 1,000 | <0,0001 |
| CD4+PD-L1+ [%] | 1 | 0 | 1,000 to 1,000 | <0,0001 |
| CD8 | 1 | 0 | 1,000 to 1,000 | <0,0001 |
| CD19 | 1 | 0 | 1,000 to 1,000 | <0,0001 |
| CD4+CTLA-4+ [%] | 0.9967 | 0.004099 | 0,9886 to 1,000 | <0,0001 |
| CD8+ | 1 | 0 | 1,000 to 1,000 | <0,0001 |
| CD19+ | 0.7272 | 0.05591 | 0,6176 to 0,8368 | 0.0068 |
| CD4+CD86+ [%] | 0.9589 | 0.0205 | 0,9187 to 0,9991 | <0,0001 |
| CD8+ | 0.9644 | 0.0189 | 0,9274 to 1,000 | <0,0001 |
| CD19+ | 0.9933 | 0.006279 | 0,9810 to 1,000 | <0,0001 |
| CD4+CD200R+ [%] | 0.8611 | 0.04674 | 0,7695 to 0,9527 | <0,0001 |
| CD8+ | 0.8611 | 0.04277 | 0,7773 to 0,9449 | <0,0001 |
| CD19+ | 0.7322 | 0.05635 | 0,6218 to 0,8427 | 0.0056 |
| CD4+CD200+ [%] | 1 | 0 | 1,000 to 1,000 | <0,0001 |
| CD8+ | 0.9867 | 0.009942 | 0,9672 to 1,000 | <0,0001 |
| CD19+ | 0.9333 | 0.02909 | 0,8763 to 0,9903 | <0,0001 |
| sPD-1 [ng/ml] | 1 | 0 | 1,000 to 1,000 | <0,0001 |
| sPD-L1 [ng/ml] | 1 | 0 | 1,000 to 1,000 | <0,0001 |
| sCTLA-4 [ng/ml] | 1 | 0 | 1,000 to 1,000 | <0,0001 |
| sCD86 [ng/ml] | 1 | 0 | 1,000 to 1,000 | <0,0001 |
| sCD200R [ng/ml] | 1 | 0 | 1,000 to 1,000 | <0,0001 |
| sCD200 [ng/ml] | 1 | 0 | 1,000 to 1,000 | <0,0001 |

**Table S14.**ROC curve analysis for CLL patients with SID and EBV reactivation

|  | miR16-5p | | | | | | | | | |
| --- | --- | --- | --- | --- | --- | --- | --- | --- | --- | --- |
|  | HV vs. SID EBV+ | HV vs. SID EBV- | HV vs. CLL EBV+ | HV vs. CLL EBV- | SID EBV+ vs. SID EBV- | SID EBV+ vs. CLL EBV+ | SID EBV+ vs. CLL EBV- | SID EBV- vs. CLL EBV+ | SID EBV- vs. CLL EBV- | CLL EBV+ vs. CLL EBV- |
| Area | 0.96 | 0.8667 | 0.5333 | 0.6933 | 0.6311 | 0.9644 | 0.9911 | 0.8622 | 0.9244 | 0.6311 |
| Std. Error | 0.0311 | 0.06787 | 0.1086 | 0.09739 | 0.1103 | 0.0284 | 0.01183 | 0.06796 | 0.04864 | 0.1079 |
| 95% confidence interval | 0,8990 to 1,000 | 0,7336 to 0,9997 | 0,3204 to 0,7462 | 0,5025 to 0,8842 | 0,4150 to 0,8472 | 0,9088 to 1,000 | 0,9679 to 1,000 | 0,7290 to 0,9954 | 0,8291 to 1,000 | 0,4196 to 0,8426 |
| P value | <0,0001 | 0.0006 | 0.7557 | 0.0712 | 0.2211 | <0,0001 | <0,0001 | 0.0007 | <0,0001 | 0.2211 |
|  |  |  |  |  |  |  |  |  |  |  |
|  | miR155-5p | | | | | | | | | |
|  | HV vs. SID EBV+ | HV vs. SID EBV- | HV vs. CLL EBV+ | HV vs. CLL EBV- | SID EBV+ vs. SID EBV- | SID EBV+ vs. CLL EBV+ | SID EBV+ vs. CLL EBV- | SID EBV- vs. CLL EBV+ | SID EBV- vs. CLL EBV- | CLL EBV+ vs. CLL EBV- |
| Area | 0.7111 | 0.7378 | 0.9733 | 0.9956 | 1 | 1 | 1 | 1 | 0.7378 | 0.9156 |
| Std. Error | 0.09698 | 0.1007 | 0.02831 | 0.007354 | 0 | 0 | 0 | 0 | 0.1007 | 0.06376 |
| 95% confidence interval | 0,5210 to 0,9012 | 0,5403 to 0,9352 | 0,9179 to 1,000 | 0,9811 to 1,000 | 1,000 to 1,000 | 1,000 to 1,000 | 1,000 to 1,000 | 1,000 to 1,000 | 0,5403 to 0,9352 | 0,7906 to 1,000 |
| P value | 0.0488 | 0.0265 | <0,0001 | <0,0001 | <0,0001 | <0,0001 | <0,0001 | <0,0001 | 0.0265 | 0.0001 |
|  |  |  |  |  |  |  |  |  |  |  |
|  | miR30c-5p | | | | | | | | | |
|  | HV vs. SID EBV+ | HV vs. SID EBV- | HV vs. CLL EBV+ | HV vs. CLL EBV- | SID EBV+ vs. SID EBV- | SID EBV+ vs. CLL EBV+ | SID EBV+ vs. CLL EBV- | SID EBV- vs. CLL EBV+ | SID EBV- vs. CLL EBV- | CLL EBV+ vs. CLL EBV- |
| Area | 0.9952 | 0.7644 | 0.5467 | 0.72 | 0.7905 | 0.9667 | 1 | 0.6933 | 0.9067 | 0.6978 |
| Std. Error | 0.007866 | 0.08618 | 0.1114 | 0.09557 | 0.09065 | 0.0345 | 0 | 0.09777 | 0.05287 | 0.1059 |
| 95% confidence interval | 0,9798 to 1,000 | 0,5955 to 0,9334 | 0,3284 to 0,7649 | 0,5327 to 0,9073 | 0,6128 to 0,9682 | 0,8990 to 1,000 | 1,000 to 1,000 | 0,5017 to 0,8850 | 0,8030 to 1,000 | 0,4902 to 0,9053 |
| P value | <0,0001 | 0.0136 | 0.6632 | 0.0401 | 0.0078 | <0,0001 | <0,0001 | 0.0712 | 0.0001 | 0.0649 |
|  |  |  |  |  |  |  |  |  |  |  |
|  | miR28-5p | | | | | | | | | |
|  | HV vs. SID EBV+ | HV vs. SID EBV- | HV vs. CLL EBV+ | HV vs. CLL EBV- | SID EBV+ vs. SID EBV- | SID EBV+ vs. CLL EBV+ | SID EBV+ vs. CLL EBV- | SID EBV- vs. CLL EBV+ | SID EBV- vs. CLL EBV- | CLL EBV+ vs. CLL EBV- |
| Area | 0.9778 | 0.8889 | 0.5022 | 0.7511 | 0.7867 | 0.9644 | 0.9911 | 0.9067 | 0.9644 | 0.8267 |
| Std. Error | 0.02425 | 0.0617 | 0.1118 | 0.09278 | 0.09458 | 0.03319 | 0.01183 | 0.06449 | 0.03088 | 0.08542 |
| 95% confidence interval | 0,9302 to 1,000 | 0,7680 to 1,000 | 0,2831 to 0,7213 | 0,5693 to 0,9330 | 0,6013 to 0,9720 | 0,8994 to 1,000 | 0,9679 to 1,000 | 0,7803 to 1,000 | 0,9039 to 1,000 | 0,6593 to 0,9941 |
| P value | <0,0001 | 0.0003 | 0.9835 | 0.0191 | 0.0075 | <0,0001 | <0,0001 | 0.0001 | <0,0001 | 0.0023 |
|  |  |  |  |  |  |  |  |  |  |  |
|  | miR181a-5p | | | | | | | | | |
|  | HV vs. SID EBV+ | HV vs. SID EBV- | HV vs. CLL EBV+ | HV vs. CLL EBV- | SID EBV+ vs. SID EBV- | SID EBV+ vs. CLL EBV+ | SID EBV+ vs. CLL EBV- | SID EBV- vs. CLL EBV+ | SID EBV- vs. CLL EBV- | CLL EBV+ vs. CLL EBV- |
| Area | 1 | 0.92 | 0.6133 | 0.7556 | 0.7956 | 1 | 0.9867 | 0.9911 | 0.9511 | 0.7467 |
| Std. Error | 0 | 0.04868 | 0.1086 | 0.09141 | 0.08663 | 0 | 0.01605 | 0.01183 | 0.0484 | 0.09757 |
| 95% confidence interval | 1,000 to 1,000 | 0,8246 to 1,000 | 0,4004 to 0,8262 | 0,5764 to 0,9347 | 0,6258 to 0,9653 | 1,000 to 1,000 | 0,9552 to 1,000 | 0,9679 to 1,000 | 0,8562 to 1,000 | 0,5554 to 0,9379 |
| P value | <0,0001 | <0,0001 | 0.2902 | 0.0171 | 0.0058 | <0,0001 | <0,0001 | <0,0001 | <0,0001 | 0.0213 |
|  |  |  |  |  |  |  |  |  |  |  |
|  | miR134-5p | | | | | | | | | |
|  | HV vs. SID EBV+ | HV vs. SID EBV- | HV vs. CLL EBV+ | HV vs. CLL EBV- | SID EBV+ vs. SID EBV- | SID EBV+ vs. CLL EBV+ | SID EBV+ vs. CLL EBV- | SID EBV- vs. CLL EBV+ | SID EBV- vs. CLL EBV- | CLL EBV+ vs. CLL EBV- |
| Area | 0.9867 | 0.7289 | 0.8667 | 0.6356 | 0.6622 | 0.6756 | 0.8578 | 0.5111 | 0.7467 | 0.7911 |
| Std. Error | 0.01605 | 0.1001 | 0.0731 | 0.1084 | 0.1016 | 0.09962 | 0.07834 | 0.1112 | 0.09072 | 0.0942 |
| 95% confidence interval | 0,9552 to 1,000 | 0,5327 to 0,9251 | 0,7234 to 1,000 | 0,4231 to 0,8480 | 0,4631 to 0,8613 | 0,4803 to 0,8708 | 0,7042 to 1,000 | 0,2931 to 0,7291 | 0,5689 to 0,9245 | 0,6065 to 0,9757 |
| P value | <0,0001 | 0.0327 | 0.0006 | 0.2058 | 0.13 | 0.1013 | 0.0008 | 0.9174 | 0.0213 | 0.0066 |
|  |  |  |  |  |  |  |  |  |  |  |
|  |  |  |  |  |  |  |  |  |  |  |
|  | miR125b-5p |  |  |  |  |  |  |  |  |  |
|  | HV vs. SID EBV+ | HV vs. SID EBV- | HV vs. CLL EBV+ | HV vs. CLL EBV- | SID EBV+ vs. SID EBV- | SID EBV+ vs. CLL EBV+ | SID EBV+ vs. CLL EBV- | SID EBV- vs. CLL EBV+ | SID EBV- vs. CLL EBV- | CLL EBV+ vs. CLL EBV- |
| Area | 0.5733 | 0.7778 | 0.9822 | 0.7644 | 0.7511 | 0.9689 | 0.7511 | 0.9956 | 0.9111 | 0.7422 |
| Std. Error | 0.1085 | 0.09092 | 0.02017 | 0.09357 | 0.09514 | 0.03234 | 0.09397 | 0.007354 | 0.05047 | 0.08982 |
| 95% confidence interval | 0,3607 to 0,7859 | 0,5996 to 0,9560 | 0,9427 to 1,000 | 0,5811 to 0,9478 | 0,5646 to 0,9376 | 0,9055 to 1,000 | 0,5669 to 0,9353 | 0,9811 to 1,000 | 0,8122 to 1,000 | 0,5662 to 0,9183 |
| P value | 0.4937 | 0.0095 | <0,0001 | 0.0136 | 0.0191 | <0,0001 | 0.0191 | <0,0001 | 0.0001 | 0.0238 |
|  |  |  |  |  |  |  |  |  |  |  |
|  |  |  |  |  |  |  |  |  |  |  |
|  | miR33a-5p |  |  |  |  |  |  |  |  |  |
|  | HV vs. SID EBV+ | HV vs. SID EBV- | HV vs. CLL EBV+ | HV vs. CLL EBV- | SID EBV+ vs. SID EBV- | SID EBV+ vs. CLL EBV+ | SID EBV+ vs. CLL EBV- | SID EBV- vs. CLL EBV+ | SID EBV- vs. CLL EBV- | CLL EBV+ vs. CLL EBV- |
| Area | 0.7733 | 0.9022 | 0.7733 | 0.52 | 0.6756 | 0.88 | 0.7289 | 0.9733 | 0.9156 | 0.8667 |
| Std. Error | 0.08981 | 0.05836 | 0.09184 | 0.1117 | 0.1073 | 0.06572 | 0.1001 | 0.02831 | 0.05126 | 0.0741 |
| 95% confidence interval | 0,5973 to 0,9493 | 0,7878 to 1,000 | 0,5933 to 0,9533 | 0,3010 to 0,7390 | 0,4653 to 0,8858 | 0,7512 to 1,000 | 0,5327 to 0,9251 | 0,9179 to 1,000 | 0,8151 to 1,000 | 0,7214 to 1,000 |
| P value | 0.0107 | 0.0002 | 0.0107 | 0.8519 | 0.1013 | 0.0004 | 0.0327 | <0,0001 | 0.0001 | 0.0006 |
|  |  |  |  |  |  |  |  |  |  |  |
|  | miR142-5p |  |  |  |  |  |  |  |  |  |
|  | HV vs. SID EBV+ | HV vs. SID EBV- | HV vs. CLL EBV+ | HV vs. CLL EBV- | SID EBV+ vs. SID EBV- | SID EBV+ vs. CLL EBV+ | SID EBV+ vs. CLL EBV- | SID EBV- vs. CLL EBV+ | SID EBV- vs. CLL EBV- | CLL EBV+ vs. CLL EBV- |
| Area | 0.9689 | 0.9333 | 0.7156 | 0.64 | 0.5644 | 1 | 0.9378 | 1 | 0.9333 | 0.5644 |
| Std. Error | 0.02602 | 0.04272 | 0.09397 | 0.1058 | 0.1083 | 0 | 0.04663 | 0 | 0.04877 | 0.1073 |
| 95% confidence interval | 0,9179 to 1,000 | 0,8496 to 1,000 | 0,5314 to 0,8997 | 0,4327 to 0,8473 | 0,3521 to 0,7768 | 1,000 to 1,000 | 0,8464 to 1,000 | 1,000 to 1,000 | 0,8378 to 1,000 | 0,3541 to 0,7748 |
| P value | <0,0001 | <0,0001 | 0.0443 | 0.1914 | 0.5476 | <0,0001 | <0,0001 | <0,0001 | <0,0001 | 0.5476 |
|  |  |  |  |  |  |  |  |  |  |  |
|  | miR144-5p |  |  |  |  |  |  |  |  |  |
|  | HV vs. SID EBV+ | HV vs. SID EBV- | HV vs. CLL EBV+ | HV vs. CLL EBV- | SID EBV+ vs. SID EBV- | SID EBV+ vs. CLL EBV+ | SID EBV+ vs. CLL EBV- | SID EBV- vs. CLL EBV+ | SID EBV- vs. CLL EBV- | CLL EBV+ vs. CLL EBV- |
| Area | 0.64 | 0.8911 | 1 | 0.8289 | 0.8933 | 0.8667 | 0.8311 | 1 | 0.5067 | 1 |
| Std. Error | 0.1074 | 0.05777 | 0 | 0.0739 | 0.05731 | 0.07285 | 0.07403 | 0 | 0.1184 | 0 |
| 95% confidence interval | 0,4296 to 0,8504 | 0,7779 to 1,000 | 1,000 to 1,000 | 0,6840 to 0,9737 | 0,7810 to 1,000 | 0,7239 to 1,000 | 0,6860 to 0,9762 | 1,000 to 1,000 | 0,2747 to 0,7386 | 1,000 to 1,000 |
| P value | 0.1914 | 0.0003 | <0,0001 | 0.0021 | 0.0002 | 0.0006 | 0.002 | <0,0001 | 0.9504 | <0,0001 |
|  |  |  |  |  |  |  |  |  |  |  |
|  | miR744-5p |  |  |  |  |  |  |  |  |  |
|  | HV vs. SID EBV+ | HV vs. SID EBV- | HV vs. CLL EBV+ | HV vs. CLL EBV- | SID EBV+ vs. SID EBV- | SID EBV+ vs. CLL EBV+ | SID EBV+ vs. CLL EBV- | SID EBV- vs. CLL EBV+ | SID EBV- vs. CLL EBV- | CLL EBV+ vs. CLL EBV- |
| Area | 0.56 | 0.9289 | 0.9111 | 0.68 | 0.8222 | 0.9733 | 0.5556 | 1 | 0.8178 | 0.9689 |
| Std. Error | 0.1075 | 0.04499 | 0.05796 | 0.1001 | 0.08478 | 0.02831 | 0.112 | 0 | 0.07678 | 0.02672 |
| 95% confidence interval | 0,3493 to 0,7707 | 0,8407 to 1,000 | 0,7975 to 1,000 | 0,4839 to 0,8761 | 0,6560 to 0,9884 | 0,9179 to 1,000 | 0,3361 to 0,7750 | 1,000 to 1,000 | 0,6673 to 0,9683 | 0,9165 to 1,000 |
| P value | 0.5755 | <0,0001 | 0.0001 | 0.093 | 0.0026 | <0,0001 | 0.6041 | <0,0001 | 0.003 | <0,0001 |
|  |  |  |  |  |  |  |  |  |  |  |
|  | miR150-5p |  |  |  |  |  |  |  |  |  |
|  | HV vs. SID EBV+ | HV vs. SID EBV- | HV vs. CLL EBV+ | HV vs. CLL EBV- | SID EBV+ vs. SID EBV- | SID EBV+ vs. CLL EBV+ | SID EBV+ vs. CLL EBV- | SID EBV- vs. CLL EBV+ | SID EBV- vs. CLL EBV- | CLL EBV+ vs. CLL EBV- |
| Area | 0.9644 | 0.7911 | 0.5689 | 0.8844 | 0.9333 | 0.9956 | 1 | 0.9422 | 1 | 0.92 |
| Std. Error | 0.03088 | 0.09361 | 0.117 | 0.06019 | 0.0538 | 0.007354 | 0 | 0.04176 | 0 | 0.0564 |
| 95% confidence interval | 0,9039 to 1,000 | 0,6076 to 0,9746 | 0,3396 to 0,7982 | 0,7665 to 1,000 | 0,8279 to 1,000 | 0,9811 to 1,000 | 1,000 to 1,000 | 0,8604 to 1,000 | 1,000 to 1,000 | 0,8095 to 1,000 |
| P value | <0,0001 | 0.0066 | 0.5203 | 0.0003 | <0,0001 | <0,0001 | <0,0001 | <0,0001 | <0,0001 | <0,0001 |
|  |  |  |  |  |  |  |  |  |  |  |
|  | miR326-5p |  |  |  |  |  |  |  |  |  |
|  | HV vs. SID EBV+ | HV vs. SID EBV- | HV vs. CLL EBV+ | HV vs. CLL EBV- | SID EBV+ vs. SID EBV- | SID EBV+ vs. CLL EBV+ | SID EBV+ vs. CLL EBV- | SID EBV- vs. CLL EBV+ | SID EBV- vs. CLL EBV- | CLL EBV+ vs. CLL EBV- |
| Area | 1 | 0.8622 | 0.5333 | 0.6933 | 0.9644 | 1 | 0.9689 | 0.92 | 0.8667 | 0.8444 |
| Std. Error | 0 | 0.06603 | 0.1139 | 0.1029 | 0.03637 | 0 | 0.02935 | 0.05406 | 0.085 | 0.08824 |
| 95% confidence interval | 1,000 to 1,000 | 0,7328 to 0,9916 | 0,3100 to 0,7566 | 0,4916 to 0,8950 | 0,8932 to 1,000 | 1,000 to 1,000 | 0,9114 to 1,000 | 0,8140 to 1,000 | 0,7001 to 1,000 | 0,6715 to 1,000 |
| P value | <0,0001 | 0.0007 | 0.7557 | 0.0712 | <0,0001 | <0,0001 | <0,0001 | <0,0001 | 0.0006 | 0.0013 |
|  |  |  |  |  |  |  |  |  |  |  |
|  | miR29a-5p |  |  |  |  |  |  |  |  |  |
|  | HV vs. SID EBV+ | HV vs. SID EBV- | HV vs. CLL EBV+ | HV vs. CLL EBV- | SID EBV+ vs. SID EBV- | SID EBV+ vs. CLL EBV+ | SID EBV+ vs. CLL EBV- | SID EBV- vs. CLL EBV+ | SID EBV- vs. CLL EBV- | CLL EBV+ vs. CLL EBV- |
| Area | 0.92 | 0.6978 | 0.88 | 0.6267 | 0.72 | 0.6844 | 0.9022 | 0.6489 | 0.7556 | 0.84 |
| Std. Error | 0.04714 | 0.0985 | 0.06137 | 0.1082 | 0.1045 | 0.1027 | 0.05511 | 0.1129 | 0.0908 | 0.08255 |
| 95% confidence interval | 0,8276 to 1,000 | 0,5047 to 0,8908 | 0,7597 to 1,000 | 0,4145 to 0,8388 | 0,5151 to 0,9249 | 0,4832 to 0,8857 | 0,7942 to 1,000 | 0,4277 to 0,8701 | 0,5776 to 0,9335 | 0,6782 to 1,000 |
| P value | <0,0001 | 0.0649 | 0.0004 | 0.2372 | 0.0401 | 0.0852 | 0.0002 | 0.1647 | 0.0171 | 0.0015 |
|  |  |  |  |  |  |  |  |  |  |  |
|  | miR21-5p |  |  |  |  |  |  |  |  |  |
|  | HV vs. SID EBV+ | HV vs. SID EBV- | HV vs. CLL EBV+ | HV vs. CLL EBV- | SID EBV+ vs. SID EBV- | SID EBV+ vs. CLL EBV+ | SID EBV+ vs. CLL EBV- | SID EBV- vs. CLL EBV+ | SID EBV- vs. CLL EBV- | CLL EBV+ vs. CLL EBV- |
| Area | 0.8844 | 0.6667 | 0.5244 | 0.8333 | 0.8089 | 0.9333 | 1 | 0.8 | 1 | 1 |
| Std. Error | 0.062 | 0.1018 | 0.1185 | 0.07987 | 0.08953 | 0.06441 | 0 | 0.1033 | 0 | 0 |
| 95% confidence interval | 0,7629 to 1,000 | 0,4672 to 0,8661 | 0,2922 to 0,7567 | 0,6768 to 0,9899 | 0,6334 to 0,9844 | 0,8071 to 1,000 | 1,000 to 1,000 | 0,5976 to 1,000 | 1,000 to 1,000 | 1,000 to 1,000 |
| P value | 0.0003 | 0.1198 | 0.8195 | 0.0019 | 0.0039 | <0,0001 | <0,0001 | 0.0051 | <0,0001 | <0,0001 |
|  |  |  |  |  |  |  |  |  |  |  |
|  | miR15a-5p |  |  |  |  |  |  |  |  |  |
|  | HV vs. SID EBV+ | HV vs. SID EBV- | HV vs. CLL EBV+ | HV vs. CLL EBV- | SID EBV+ vs. SID EBV- | SID EBV+ vs. CLL EBV+ | SID EBV+ vs. CLL EBV- | SID EBV- vs. CLL EBV+ | SID EBV- vs. CLL EBV- | CLL EBV+ vs. CLL EBV- |
| Area | 0.9689 | 0.8978 | 0.5111 | 0.5378 | 0.6889 | 0.9733 | 0.9867 | 0.9378 | 0.9422 | 0.5422 |
| Std. Error | 0.02602 | 0.05881 | 0.1094 | 0.1081 | 0.1031 | 0.02408 | 0.01605 | 0.04663 | 0.03949 | 0.1079 |
| 95% confidence interval | 0,9179 to 1,000 | 0,7825 to 1,000 | 0,2967 to 0,7255 | 0,3259 to 0,7496 | 0,4869 to 0,8909 | 0,9261 to 1,000 | 0,9552 to 1,000 | 0,8464 to 1,000 | 0,8648 to 1,000 | 0,3308 to 0,7537 |
| P value | <0,0001 | 0.0002 | 0.9174 | 0.7244 | 0.0779 | <0,0001 | <0,0001 | <0,0001 | <0,0001 | 0.6936 |
|  |  |  |  |  |  |  |  |  |  |  |
|  | miR221-5p |  |  |  |  |  |  |  |  |  |
|  | HV vs. SID EBV+ | HV vs. SID EBV- | HV vs. CLL EBV+ | HV vs. CLL EBV- | SID EBV+ vs. SID EBV- | SID EBV+ vs. CLL EBV+ | SID EBV+ vs. CLL EBV- | SID EBV- vs. CLL EBV+ | SID EBV- vs. CLL EBV- | CLL EBV+ vs. CLL EBV- |
| Area | 0.5489 | 0.5911 | 0.9267 | 0.9178 | 0.6578 | 0.94 | 0.9311 | 0.9422 | 0.9422 | 0.68 |
| Std. Error | 0.1085 | 0.1065 | 0.05951 | 0.06081 | 0.1041 | 0.05845 | 0.0591 | 0.05652 | 0.05652 | 0.1046 |
| 95% confidence interval | 0,3363 to 0,7615 | 0,3823 to 0,7999 | 0,8100 to 1,000 | 0,7986 to 1,000 | 0,4538 to 0,8618 | 0,8254 to 1,000 | 0,8153 to 1,000 | 0,8315 to 1,000 | 0,8315 to 1,000 | 0,4751 to 0,8849 |
| P value | 0.6482 | 0.3952 | <0,0001 | <0,0001 | 0.1409 | <0,0001 | <0,0001 | <0,0001 | <0,0001 | 0.093 |
|  |  |  |  |  |  |  |  |  |  |  |
|  | miR486-5p |  |  |  |  |  |  |  |  |  |
|  | HV vs. SID EBV+ | HV vs. SID EBV- | HV vs. CLL EBV+ | HV vs. CLL EBV- | SID EBV+ vs. SID EBV- | SID EBV+ vs. CLL EBV+ | SID EBV+ vs. CLL EBV- | SID EBV- vs. CLL EBV+ | SID EBV- vs. CLL EBV- | CLL EBV+ vs. CLL EBV- |
| Area | 0.9867 | 0.64 | 0.5111 | 0.7778 | 0.9956 | 0.9956 | 1 | 0.7067 | 0.8533 | 0.7689 |
| Std. Error | 0.01486 | 0.1077 | 0.11 | 0.08591 | 0.007354 | 0.007354 | 0 | 0.09952 | 0.07998 | 0.09508 |
| 95% confidence interval | 0,9575 to 1,000 | 0,4290 to 0,8510 | 0,2954 to 0,7268 | 0,6094 to 0,9462 | 0,9811 to 1,000 | 0,9811 to 1,000 | 1,000 to 1,000 | 0,5116 to 0,9017 | 0,6966 to 1,000 | 0,5825 to 0,9552 |
| P value | <0,0001 | 0.1914 | 0.9174 | 0.0095 | <0,0001 | <0,0001 | <0,0001 | 0.0538 | 0.001 | 0.0121 |
|  |  |  |  |  |  |  |  |  |  |  |
|  | CD4+PD-1+ [%] |  |  |  |  |  |  |  |  |  |
|  | HV vs. SID EBV+ | HV vs. SID EBV- | HV vs. CLL EBV+ | HV vs. CLL EBV- | SID EBV+ vs. SID EBV- | SID EBV+ vs. CLL EBV+ | SID EBV+ vs. CLL EBV- | SID EBV- vs. CLL EBV+ | SID EBV- vs. CLL EBV- | CLL EBV+ vs. CLL EBV- |
| Area | 1 | 1 | 1 | 1 | 1 | 1 | 1 | 1 | 0.64 | 1 |
| Std. Error | 0 | 0 | 0 | 0 | 0 | 0 | 0 | 0 | 0.1052 | 0 |
| 95% confidence interval | 1,000 to 1,000 | 1,000 to 1,000 | 1,000 to 1,000 | 1,000 to 1,000 | 1,000 to 1,000 | 1,000 to 1,000 | 1,000 to 1,000 | 1,000 to 1,000 | 0,4337 to 0,8463 | 1,000 to 1,000 |
| P value | <0,0001 | <0,0001 | <0,0001 | <0,0001 | <0,0001 | <0,0001 | <0,0001 | <0,0001 | 0.1914 | <0,0001 |
|  |  |  |  |  |  |  |  |  |  |  |
|  | CD8+PD-1+ [%] |  |  |  |  |  |  |  |  |  |
|  | HV vs. SID EBV+ | HV vs. SID EBV- | HV vs. CLL EBV+ | HV vs. CLL EBV- | SID EBV+ vs. SID EBV- | SID EBV+ vs. CLL EBV+ | SID EBV+ vs. CLL EBV- | SID EBV- vs. CLL EBV+ | SID EBV- vs. CLL EBV- | CLL EBV+ vs. CLL EBV- |
| Area | 1 | 1 | 1 | 1 | 1 | 0.6 | 1 | 1 | 0.7644 | 1 |
| Std. Error | 0 | 0 | 0 | 0 | 0 | 0.1083 | 0 | 0 | 0.09077 | 0 |
| 95% confidence interval | 1,000 to 1,000 | 1,000 to 1,000 | 1,000 to 1,000 | 1,000 to 1,000 | 1,000 to 1,000 | 0,3878 to 0,8122 | 1,000 to 1,000 | 1,000 to 1,000 | 0,5865 to 0,9423 | 1,000 to 1,000 |
| P value | <0,0001 | <0,0001 | <0,0001 | <0,0001 | <0,0001 | 0.3507 | <0,0001 | <0,0001 | 0.0136 | <0,0001 |
|  |  |  |  |  |  |  |  |  |  |  |
|  | CD19+PD-1+ [%] |  |  |  |  |  |  |  |  |  |
|  | HV vs. SID EBV+ | HV vs. SID EBV- | HV vs. CLL EBV+ | HV vs. CLL EBV- | SID EBV+ vs. SID EBV- | SID EBV+ vs. CLL EBV+ | SID EBV+ vs. CLL EBV- | SID EBV- vs. CLL EBV+ | SID EBV- vs. CLL EBV- | CLL EBV+ vs. CLL EBV- |
| Area | 1 | 1 | 1 | 1 | 1 | 0.8 | 1 | 0.9778 | 0.6356 | 0.9822 |
| Std. Error | 0 | 0 | 0 | 0 | 0 | 0.07993 | 0 | 0.02425 | 0.1028 | 0.02017 |
| 95% confidence interval | 1,000 to 1,000 | 1,000 to 1,000 | 1,000 to 1,000 | 1,000 to 1,000 | 1,000 to 1,000 | 0,6433 to 0,9567 | 1,000 to 1,000 | 0,9302 to 1,000 | 0,4341 to 0,8371 | 0,9427 to 1,000 |
| P value | <0,0001 | <0,0001 | <0,0001 | <0,0001 | <0,0001 | 0.0051 | <0,0001 | <0,0001 | 0.2058 | <0,0001 |
|  |  |  |  |  |  |  |  |  |  |  |
|  | CD4+PD-L1+ [%] |  |  |  |  |  |  |  |  |  |
|  | HV vs. SID EBV+ | HV vs. SID EBV- | HV vs. CLL EBV+ | HV vs. CLL EBV- | SID EBV+ vs. SID EBV- | SID EBV+ vs. CLL EBV+ | SID EBV+ vs. CLL EBV- | SID EBV- vs. CLL EBV+ | SID EBV- vs. CLL EBV- | CLL EBV+ vs. CLL EBV- |
| Area | 1 | 1 | 1 | 1 | 1 | 0.8356 | 1 | 1 | 0.6711 | 1 |
| Std. Error | 0 | 0 | 0 | 0 | 0 | 0.07395 | 0 | 0 | 0.1022 | 0 |
| 95% confidence interval | 1,000 to 1,000 | 1,000 to 1,000 | 1,000 to 1,000 | 1,000 to 1,000 | 1,000 to 1,000 | 0,6906 to 0,9805 | 1,000 to 1,000 | 1,000 to 1,000 | 0,4707 to 0,8715 | 1,000 to 1,000 |
| P value | <0,0001 | <0,0001 | <0,0001 | <0,0001 | <0,0001 | 0.0017 | <0,0001 | <0,0001 | 0.1103 | <0,0001 |
|  |  |  |  |  |  |  |  |  |  |  |
|  | CD8+PD-L1+ [%] |  |  |  |  |  |  |  |  |  |
|  | HV vs. SID EBV+ | HV vs. SID EBV- | HV vs. CLL EBV+ | HV vs. CLL EBV- | SID EBV+ vs. SID EBV- | SID EBV+ vs. CLL EBV+ | SID EBV+ vs. CLL EBV- | SID EBV- vs. CLL EBV+ | SID EBV- vs. CLL EBV- | CLL EBV+ vs. CLL EBV- |
| Area | 1 | 1 | 1 | 1 | 1 | 0.6889 | 1 | 1 | 0.7689 | 1 |
| Std. Error | 0 | 0 | 0 | 0 | 0 | 0.09907 | 0 | 0 | 0.08697 | 0 |
| 95% confidence interval | 1,000 to 1,000 | 1,000 to 1,000 | 1,000 to 1,000 | 1,000 to 1,000 | 1,000 to 1,000 | 0,4947 to 0,8831 | 1,000 to 1,000 | 1,000 to 1,000 | 0,5984 to 0,9394 | 1,000 to 1,000 |
| P value | <0,0001 | <0,0001 | <0,0001 | <0,0001 | <0,0001 | 0.0779 | <0,0001 | <0,0001 | 0.0121 | <0,0001 |
|  |  |  |  |  |  |  |  |  |  |  |
|  | CD19+PD-L1+ [%] |  |  |  |  |  |  |  |  |  |
|  | HV vs. SID EBV+ | HV vs. SID EBV- | HV vs. CLL EBV+ | HV vs. CLL EBV- | SID EBV+ vs. SID EBV- | SID EBV+ vs. CLL EBV+ | SID EBV+ vs. CLL EBV- | SID EBV- vs. CLL EBV+ | SID EBV- vs. CLL EBV- | CLL EBV+ vs. CLL EBV- |
| Area | 1 | 1 | 1 | 1 | 1 | 0.6844 | 1 | 1 | 0.6933 | 1 |
| Std. Error | 0 | 0 | 0 | 0 | 0 | 0.09753 | 0 | 0 | 0.09777 | 0 |
| 95% confidence interval | 1,000 to 1,000 | 1,000 to 1,000 | 1,000 to 1,000 | 1,000 to 1,000 | 1,000 to 1,000 | 0,4933 to 0,8756 | 1,000 to 1,000 | 1,000 to 1,000 | 0,5017 to 0,8850 | 1,000 to 1,000 |
| P value | <0,0001 | <0,0001 | <0,0001 | <0,0001 | <0,0001 | 0.0852 | <0,0001 | <0,0001 | 0.0712 | <0,0001 |
|  |  |  |  |  |  |  |  |  |  |  |
|  | CD4+CTLA-4+ [%] |  |  |  |  |  |  |  |  |  |
|  | HV vs. SID EBV+ | HV vs. SID EBV- | HV vs. CLL EBV+ | HV vs. CLL EBV- | SID EBV+ vs. SID EBV- | SID EBV+ vs. CLL EBV+ | SID EBV+ vs. CLL EBV- | SID EBV- vs. CLL EBV+ | SID EBV- vs. CLL EBV- | CLL EBV+ vs. CLL EBV- |
| Area | 1 | 1 | 1 | 1 | 1 | 0.5911 | 1 | 1 | 0.6178 | 1 |
| Std. Error | 0 | 0 | 0 | 0 | 0 | 0.1091 | 0 | 0 | 0.1059 | 0 |
| 95% confidence interval | 1,000 to 1,000 | 1,000 to 1,000 | 1,000 to 1,000 | 1,000 to 1,000 | 1,000 to 1,000 | 0,3773 to 0,8050 | 1,000 to 1,000 | 1,000 to 1,000 | 0,4101 to 0,8254 | 1,000 to 1,000 |
| P value | <0,0001 | <0,0001 | <0,0001 | <0,0001 | <0,0001 | 0.3952 | <0,0001 | <0,0001 | 0.2717 | <0,0001 |
|  |  |  |  |  |  |  |  |  |  |  |
|  | CD8+CTLA-4+ [%] |  |  |  |  |  |  |  |  |  |
|  | HV vs. SID EBV+ | HV vs. SID EBV- | HV vs. CLL EBV+ | HV vs. CLL EBV- | SID EBV+ vs. SID EBV- | SID EBV+ vs. CLL EBV+ | SID EBV+ vs. CLL EBV- | SID EBV- vs. CLL EBV+ | SID EBV- vs. CLL EBV- | CLL EBV+ vs. CLL EBV- |
| Area | 1 | 1 | 1 | 1 | 1 | 0.6933 | 1 | 1 | 0.5378 | 1 |
| Std. Error | 0 | 0 | 0 | 0 | 0 | 0.09682 | 0 | 0 | 0.119 | 0 |
| 95% confidence interval | 1,000 to 1,000 | 1,000 to 1,000 | 1,000 to 1,000 | 1,000 to 1,000 | 1,000 to 1,000 | 0,5036 to 0,8831 | 1,000 to 1,000 | 1,000 to 1,000 | 0,3046 to 0,7710 | 1,000 to 1,000 |
| P value | <0,0001 | <0,0001 | <0,0001 | <0,0001 | <0,0001 | 0.0712 | <0,0001 | <0,0001 | 0.7244 | <0,0001 |
|  |  |  |  |  |  |  |  |  |  |  |
|  | CD19+CTLA-4+ [%] | |  |  |  |  |  |  |  |  |
|  | HV vs. SID EBV+ | HV vs. SID EBV- | HV vs. CLL EBV+ | HV vs. CLL EBV- | SID EBV+ vs. SID EBV- | SID EBV+ vs. CLL EBV+ | SID EBV+ vs. CLL EBV- | SID EBV- vs. CLL EBV+ | SID EBV- vs. CLL EBV- | CLL EBV+ vs. CLL EBV- |
| Area | 1 | 0.5378 | 1 | 0.5533 | 1 | 0.7467 | 1 | 1 | 0.5333 | 1 |
| Std. Error | 0 | 0.1124 | 0 | 0.1149 | 0 | 0.08949 | 0 | 0 | 0.1116 | 0 |
| 95% confidence interval | 1,000 to 1,000 | 0,3174 to 0,7582 | 1,000 to 1,000 | 0,3282 to 0,7784 | 1,000 to 1,000 | 0,5713 to 0,9221 | 1,000 to 1,000 | 1,000 to 1,000 | 0,3145 to 0,7521 | 1,000 to 1,000 |
| P value | <0,0001 | 0.7244 | <0,0001 | 0.6187 | <0,0001 | 0.0213 | <0,0001 | <0,0001 | 0.7557 | <0,0001 |
|  |  |  |  |  |  |  |  |  |  |  |
|  | CD4+CD86+ [%] |  |  |  |  |  |  |  |  |  |
|  | HV vs. SID EBV+ | HV vs. SID EBV- | HV vs. CLL EBV+ | HV vs. CLL EBV- | SID EBV+ vs. SID EBV- | SID EBV+ vs. CLL EBV+ | SID EBV+ vs. CLL EBV- | SID EBV- vs. CLL EBV+ | SID EBV- vs. CLL EBV- | CLL EBV+ vs. CLL EBV- |
| Area | 1 | 1 | 1 | 1 | 1 | 0.6844 | 1 | 1 | 0.7133 | 1 |
| Std. Error | 0 | 0 | 0 | 0 | 0 | 0.09772 | 0 | 0 | 0.09584 | 0 |
| 95% confidence interval | 1,000 to 1,000 | 1,000 to 1,000 | 1,000 to 1,000 | 1,000 to 1,000 | 1,000 to 1,000 | 0,4929 to 0,8760 | 1,000 to 1,000 | 1,000 to 1,000 | 0,5255 to 0,9012 | 1,000 to 1,000 |
| P value | <0,0001 | <0,0001 | <0,0001 | <0,0001 | <0,0001 | 0.0852 | <0,0001 | <0,0001 | 0.0465 | <0,0001 |
|  |  |  |  |  |  |  |  |  |  |  |
|  | CD8+CD86+ [%] |  |  |  |  |  |  |  |  |  |
|  | HV vs. SID EBV+ | HV vs. SID EBV- | HV vs. CLL EBV+ | HV vs. CLL EBV- | SID EBV+ vs. SID EBV- | SID EBV+ vs. CLL EBV+ | SID EBV+ vs. CLL EBV- | SID EBV- vs. CLL EBV+ | SID EBV- vs. CLL EBV- | CLL EBV+ vs. CLL EBV- |
| Area | 1 | 1 | 1 | 1 | 1 | 0.6044 | 1 | 0.9956 | 0.8 | 0.9956 |
| Std. Error | 0 | 0 | 0 | 0 | 0 | 0.1053 | 0 | 0.007354 | 0.08398 | 0.007354 |
| 95% confidence interval | 1,000 to 1,000 | 1,000 to 1,000 | 1,000 to 1,000 | 1,000 to 1,000 | 1,000 to 1,000 | 0,3980 to 0,8108 | 1,000 to 1,000 | 0,9811 to 1,000 | 0,6354 to 0,9646 | 0,9811 to 1,000 |
| P value | <0,0001 | <0,0001 | <0,0001 | <0,0001 | <0,0001 | 0.3297 | <0,0001 | <0,0001 | 0.0051 | <0,0001 |
|  |  |  |  |  |  |  |  |  |  |  |
|  |  |  |  |  |  |  |  |  |  |  |
|  | CD19+CD86+ [%] |  |  |  |  |  |  |  |  |  |
|  | HV vs. SID EBV+ | HV vs. SID EBV- | HV vs. CLL EBV+ | HV vs. CLL EBV- | SID EBV+ vs. SID EBV- | SID EBV+ vs. CLL EBV+ | SID EBV+ vs. CLL EBV- | SID EBV- vs. CLL EBV+ | SID EBV- vs. CLL EBV- | CLL EBV+ vs. CLL EBV- |
| Area | 1 | 1 | 1 | 1 | 1 | 0.8578 | 1 | 1 | 1 | 1 |
| Std. Error | 0 | 0 | 0 | 0 | 0 | 0.06608 | 0 | 0 | 0 | 0 |
| 95% confidence interval | 1,000 to 1,000 | 1,000 to 1,000 | 1,000 to 1,000 | 1,000 to 1,000 | 1,000 to 1,000 | 0,7283 to 0,9873 | 1,000 to 1,000 | 1,000 to 1,000 | 1,000 to 1,000 | 1,000 to 1,000 |
| P value | <0,0001 | <0,0001 | <0,0001 | <0,0001 | <0,0001 | 0.0008 | <0,0001 | <0,0001 | <0,0001 | <0,0001 |
|  |  |  |  |  |  |  |  |  |  |  |
|  | CD4+CD200R+ [%] | |  |  |  |  |  |  |  |  |
|  | HV vs. SID EBV+ | HV vs. SID EBV- | HV vs. CLL EBV+ | HV vs. CLL EBV- | SID EBV+ vs. SID EBV- | SID EBV+ vs. CLL EBV+ | SID EBV+ vs. CLL EBV- | SID EBV- vs. CLL EBV+ | SID EBV- vs. CLL EBV- | CLL EBV+ vs. CLL EBV- |
| Area | 1 | 1 | 1 | 0.6756 | 1 | 0.5556 | 1 | 1 | 0.5689 | 1 |
| Std. Error | 0 | 0 | 0 | 0.1015 | 0 | 0.1097 | 0 | 0 | 0.1077 | 0 |
| 95% confidence interval | 1,000 to 1,000 | 1,000 to 1,000 | 1,000 to 1,000 | 0,4767 to 0,8744 | 1,000 to 1,000 | 0,3406 to 0,7705 | 1,000 to 1,000 | 1,000 to 1,000 | 0,3579 to 0,7799 | 1,000 to 1,000 |
| P value | <0,0001 | <0,0001 | <0,0001 | 0.1013 | <0,0001 | 0.6041 | <0,0001 | <0,0001 | 0.5203 | <0,0001 |
|  |  |  |  |  |  |  |  |  |  |  |
|  | CD8+CD200R+ [%] | |  |  |  |  |  |  |  |  |
|  | HV vs. SID EBV+ | HV vs. SID EBV- | HV vs. CLL EBV+ | HV vs. CLL EBV- | SID EBV+ vs. SID EBV- | SID EBV+ vs. CLL EBV+ | SID EBV+ vs. CLL EBV- | SID EBV- vs. CLL EBV+ | SID EBV- vs. CLL EBV- | CLL EBV+ vs. CLL EBV- |
| Area | 1 | 1 | 1 | 0.6578 | 1 | 0.6844 | 1 | 1 | 0.6244 | 1 |
| Std. Error | 0 | 0 | 0 | 0.1041 | 0 | 0.09866 | 0 | 0 | 0.1075 | 0 |
| 95% confidence interval | 1,000 to 1,000 | 1,000 to 1,000 | 1,000 to 1,000 | 0,4538 to 0,8618 | 1,000 to 1,000 | 0,4911 to 0,8778 | 1,000 to 1,000 | 1,000 to 1,000 | 0,4137 to 0,8352 | 1,000 to 1,000 |
| P value | <0,0001 | <0,0001 | <0,0001 | 0.1409 | <0,0001 | 0.0852 | <0,0001 | <0,0001 | 0.2455 | <0,0001 |
|  |  |  |  |  |  |  |  |  |  |  |
|  |  |  |  |  |  |  |  |  |  |  |
|  | CD19+CD200R+ [%] | |  |  |  |  |  |  |  |  |
|  | HV vs. SID EBV+ | HV vs. SID EBV- | HV vs. CLL EBV+ | HV vs. CLL EBV- | SID EBV+ vs. SID EBV- | SID EBV+ vs. CLL EBV+ | SID EBV+ vs. CLL EBV- | SID EBV- vs. CLL EBV+ | SID EBV- vs. CLL EBV- | CLL EBV+ vs. CLL EBV- |
| Area | 0.6356 | 1 | 1 | 0.5644 | 1 | 0.7022 | 1 | 1 | 0.72 | 1 |
| Std. Error | 0.1049 | 0 | 0 | 0.1071 | 0 | 0.096 | 0 | 0 | 0.09823 | 0 |
| 95% confidence interval | 0,4299 to 0,8412 | 1,000 to 1,000 | 1,000 to 1,000 | 0,3544 to 0,7745 | 1,000 to 1,000 | 0,5141 to 0,8904 | 1,000 to 1,000 | 1,000 to 1,000 | 0,5275 to 0,9125 | 1,000 to 1,000 |
| P value | 0.2058 | <0,0001 | <0,0001 | 0.5476 | <0,0001 | 0.0591 | <0,0001 | <0,0001 | 0.0401 | <0,0001 |
|  |  |  |  |  |  |  |  |  |  |  |
|  |  |  |  |  |  |  |  |  |  |  |
|  | CD4+CD200+ [%] |  |  |  |  |  |  |  |  |  |
|  | HV vs. SID EBV+ | HV vs. SID EBV- | HV vs. CLL EBV+ | HV vs. CLL EBV- | SID EBV+ vs. SID EBV- | SID EBV+ vs. CLL EBV+ | SID EBV+ vs. CLL EBV- | SID EBV- vs. CLL EBV+ | SID EBV- vs. CLL EBV- | CLL EBV+ vs. CLL EBV- |
| Area | 1 | 1 | 1 | 1 | 1 | 0.6222 | 1 | 1 | 0.7444 | 1 |
| Std. Error | 0 | 0 | 0 | 0 | 0 | 0.1049 | 0 | 0 | 0.08993 | 0 |
| 95% confidence interval | 1,000 to 1,000 | 1,000 to 1,000 | 1,000 to 1,000 | 1,000 to 1,000 | 1,000 to 1,000 | 0,4165 to 0,8279 | 1,000 to 1,000 | 1,000 to 1,000 | 0,5682 to 0,9207 | 1,000 to 1,000 |
| P value | <0,0001 | <0,0001 | <0,0001 | <0,0001 | <0,0001 | 0.254 | <0,0001 | <0,0001 | 0.0225 | <0,0001 |
|  |  |  |  |  |  |  |  |  |  |  |
|  |  |  |  |  |  |  |  |  |  |  |
|  | CD8+CD200+ [%] |  |  |  |  |  |  |  |  |  |
|  | HV vs. SID EBV+ | HV vs. SID EBV- | HV vs. CLL EBV+ | HV vs. CLL EBV- | SID EBV+ vs. SID EBV- | SID EBV+ vs. CLL EBV+ | SID EBV+ vs. CLL EBV- | SID EBV- vs. CLL EBV+ | SID EBV- vs. CLL EBV- | CLL EBV+ vs. CLL EBV- |
| Area | 1 | 1 | 1 | 1 | 1 | 0.8578 | 1 | 1 | 0.5422 | 1 |
| Std. Error | 0 | 0 | 0 | 0 | 0 | 0.07323 | 0 | 0 | 0.1087 | 0 |
| 95% confidence interval | 1,000 to 1,000 | 1,000 to 1,000 | 1,000 to 1,000 | 1,000 to 1,000 | 1,000 to 1,000 | 0,7142 to 1,000 | 1,000 to 1,000 | 1,000 to 1,000 | 0,3291 to 0,7553 | 1,000 to 1,000 |
| P value | <0,0001 | <0,0001 | <0,0001 | <0,0001 | <0,0001 | 0.0008 | <0,0001 | <0,0001 | 0.6936 | <0,0001 |
|  |  |  |  |  |  |  |  |  |  |  |
|  |  |  |  |  |  |  |  |  |  |  |
|  | CD19+CD200+ [%] | |  |  |  |  |  |  |  |  |
|  | HV vs. SID EBV+ | HV vs. SID EBV- | HV vs. CLL EBV+ | HV vs. CLL EBV- | SID EBV+ vs. SID EBV- | SID EBV+ vs. CLL EBV+ | SID EBV+ vs. CLL EBV- | SID EBV- vs. CLL EBV+ | SID EBV- vs. CLL EBV- | CLL EBV+ vs. CLL EBV- |
| Area | 1 | 1 | 1 | 1 | 1 | 0.8933 | 1 | 1 | 0.7956 | 1 |
| Std. Error | 0 | 0 | 0 | 0 | 0 | 0.06313 | 0 | 0 | 0.08578 | 0 |
| 95% confidence interval | 1,000 to 1,000 | 1,000 to 1,000 | 1,000 to 1,000 | 1,000 to 1,000 | 1,000 to 1,000 | 0,7696 to 1,000 | 1,000 to 1,000 | 1,000 to 1,000 | 0,6274 to 0,9637 | 1,000 to 1,000 |
| P value | <0,0001 | <0,0001 | <0,0001 | <0,0001 | <0,0001 | 0.0002 | <0,0001 | <0,0001 | 0.0058 | <0,0001 |
|  |  |  |  |  |  |  |  |  |  |  |
|  |  |  |  |  |  |  |  |  |  |  |
|  | sPD-1 [ng/ml] |  |  |  |  |  |  |  |  |  |
|  | HV vs. SID EBV+ | HV vs. SID EBV- | HV vs. CLL EBV+ | HV vs. CLL EBV- | SID EBV+ vs. SID EBV- | SID EBV+ vs. CLL EBV+ | SID EBV+ vs. CLL EBV- | SID EBV- vs. CLL EBV+ | SID EBV- vs. CLL EBV- | CLL EBV+ vs. CLL EBV- |
| Area | 1 | 1 | 1 | 1 | 1 | 0.8311 | 1 | 1 | 0.6311 | 1 |
| Std. Error | 0 | 0 | 0 | 0 | 0 | 0.07576 | 0 | 0 | 0.1096 | 0 |
| 95% confidence interval | 1,000 to 1,000 | 1,000 to 1,000 | 1,000 to 1,000 | 1,000 to 1,000 | 1,000 to 1,000 | 0,6826 to 0,9796 | 1,000 to 1,000 | 1,000 to 1,000 | 0,4163 to 0,8459 | 1,000 to 1,000 |
| P value | <0,0001 | <0,0001 | <0,0001 | <0,0001 | <0,0001 | 0.002 | <0,0001 | <0,0001 | 0.2211 | <0,0001 |
|  |  |  |  |  |  |  |  |  |  |  |
|  |  |  |  |  |  |  |  |  |  |  |
|  | sPD-L1 [ng/ml] |  |  |  |  |  |  |  |  |  |
|  | HV vs. SID EBV+ | HV vs. SID EBV- | HV vs. CLL EBV+ | HV vs. CLL EBV- | SID EBV+ vs. SID EBV- | SID EBV+ vs. CLL EBV+ | SID EBV+ vs. CLL EBV- | SID EBV- vs. CLL EBV+ | SID EBV- vs. CLL EBV- | CLL EBV+ vs. CLL EBV- |
| Area | 1 | 1 | 1 | 1 | 0.9333 | 0.7689 | 0.9333 | 1 | 0.7867 | 1 |
| Std. Error | 0 | 0 | 0 | 0 | 0.06441 | 0.08989 | 0.06441 | 0 | 0.08558 | 0 |
| 95% confidence interval | 1,000 to 1,000 | 1,000 to 1,000 | 1,000 to 1,000 | 1,000 to 1,000 | 0,8071 to 1,000 | 0,5927 to 0,9451 | 0,8071 to 1,000 | 1,000 to 1,000 | 0,6189 to 0,9544 | 1,000 to 1,000 |
| P value | <0,0001 | <0,0001 | <0,0001 | <0,0001 | <0,0001 | 0.0121 | <0,0001 | <0,0001 | 0.0075 | <0,0001 |
|  |  |  |  |  |  |  |  |  |  |  |
|  | sCTLA-4 [ng/ml] |  |  |  |  |  |  |  |  |  |
|  | HV vs. SID EBV+ | HV vs. SID EBV- | HV vs. CLL EBV+ | HV vs. CLL EBV- | SID EBV+ vs. SID EBV- | SID EBV+ vs. CLL EBV+ | SID EBV+ vs. CLL EBV- | SID EBV- vs. CLL EBV+ | SID EBV- vs. CLL EBV- | CLL EBV+ vs. CLL EBV- |
| Area | 1 | 1 | 1 | 1 | 0.9556 | 0.8222 | 0.9867 | 0.9022 | 0.6489 | 0.9333 |
| Std. Error | 0 | 0 | 0 | 0 | 0.03814 | 0.07485 | 0.01486 | 0.07013 | 0.1045 | 0.04839 |
| 95% confidence interval | 1,000 to 1,000 | 1,000 to 1,000 | 1,000 to 1,000 | 1,000 to 1,000 | 0,8808 to 1,000 | 0,6755 to 0,9689 | 0,9575 to 1,000 | 0,7648 to 1,000 | 0,4440 to 0,8538 | 0,8385 to 1,000 |
| P value | <0,0001 | <0,0001 | <0,0001 | <0,0001 | <0,0001 | 0.0026 | <0,0001 | 0.0002 | 0.1647 | <0,0001 |
|  |  |  |  |  |  |  |  |  |  |  |
|  |  |  |  |  |  |  |  |  |  |  |
|  | sCD86 [ng/ml] |  |  |  |  |  |  |  |  |  |
|  | HV vs. SID EBV+ | HV vs. SID EBV- | HV vs. CLL EBV+ | HV vs. CLL EBV- | SID EBV+ vs. SID EBV- | SID EBV+ vs. CLL EBV+ | SID EBV+ vs. CLL EBV- | SID EBV- vs. CLL EBV+ | SID EBV- vs. CLL EBV- | CLL EBV+ vs. CLL EBV- |
| Area | 1 | 1 | 1 | 1 | 1 | 0.9289 | 1 | 1 | 0.6711 | 1 |
| Std. Error | 0 | 0 | 0 | 0 | 0 | 0.04458 | 0 | 0 | 0.1047 | 0 |
| 95% confidence interval | 1,000 to 1,000 | 1,000 to 1,000 | 1,000 to 1,000 | 1,000 to 1,000 | 1,000 to 1,000 | 0,8415 to 1,000 | 1,000 to 1,000 | 1,000 to 1,000 | 0,4658 to 0,8764 | 1,000 to 1,000 |
| P value | <0,0001 | <0,0001 | <0,0001 | <0,0001 | <0,0001 | <0,0001 | <0,0001 | <0,0001 | 0.1103 | <0,0001 |
|  |  |  |  |  |  |  |  |  |  |  |
|  | sCD200R [ng/ml] |  |  |  |  |  |  |  |  |  |
|  | HV vs. SID EBV+ | HV vs. SID EBV- | HV vs. CLL EBV+ | HV vs. CLL EBV- | SID EBV+ vs. SID EBV- | SID EBV+ vs. CLL EBV+ | SID EBV+ vs. CLL EBV- | SID EBV- vs. CLL EBV+ | SID EBV- vs. CLL EBV- | CLL EBV+ vs. CLL EBV- |
| Area | 1 | 1 | 1 | 1 | 1 | 0.8578 | 1 | 1 | 0.6667 | 1 |
| Std. Error | 0 | 0 | 0 | 0 | 0 | 0.06692 | 0 | 0 | 0.103 | 0 |
| 95% confidence interval | 1,000 to 1,000 | 1,000 to 1,000 | 1,000 to 1,000 | 1,000 to 1,000 | 1,000 to 1,000 | 0,7266 to 0,9889 | 1,000 to 1,000 | 1,000 to 1,000 | 0,4648 to 0,8686 | 1,000 to 1,000 |
| P value | <0,0001 | <0,0001 | <0,0001 | <0,0001 | <0,0001 | 0.0008 | <0,0001 | <0,0001 | 0.1198 | <0,0001 |
|  |  |  |  |  |  |  |  |  |  |  |
|  | sCD200 [ng/ml] |  |  |  |  |  |  |  |  |  |
|  | HV vs. SID EBV+ | HV vs. SID EBV- | HV vs. CLL EBV+ | HV vs. CLL EBV- | SID EBV+ vs. SID EBV- | SID EBV+ vs. CLL EBV+ | SID EBV+ vs. CLL EBV- | SID EBV- vs. CLL EBV+ | SID EBV- vs. CLL EBV- | CLL EBV+ vs. CLL EBV- |
| Area | 1 | 1 | 1 | 1 | 1 | 0.7378 | 1 | 1 | 0.72 | 1 |
| Std. Error | 0 | 0 | 0 | 0 | 0 | 0.09134 | 0 | 0 | 0.09499 | 0 |
| 95% confidence interval | 1,000 to 1,000 | 1,000 to 1,000 | 1,000 to 1,000 | 1,000 to 1,000 | 1,000 to 1,000 | 0,5588 to 0,9168 | 1,000 to 1,000 | 1,000 to 1,000 | 0,5338 to 0,9062 | 1,000 to 1,000 |
| P value | <0,0001 | <0,0001 | <0,0001 | <0,0001 | <0,0001 | 0.0265 | <0,0001 | <0,0001 | 0.0401 | <0,0001 |
